# Light‐Enhanced Chloride Transport by Thiourea‐Appended Molecular Motor

**DOI:** 10.1002/anie.4755839

**Published:** 2026-08-22

**Authors:** Abhishek Mondal, Sander J. Wezenberg

**Affiliations:** ^1^ Leiden Institute of Chemistry Leiden University Leiden The Netherlands

**Keywords:** ion transport, lipid bilayers, molecular motors, overcrowded alkenes, photochromism

## Abstract

The regulation of solute transport across lipid bilayer membranes is central to many biological processes and is typically mediated by protein transporters that dynamically respond to stimuli. The development of artificial systems that emulate such responsive behavior remains a major challenge. Here, we show enhancement of chloride transport under light irradiation using a Feringa‐type molecular rotary motor connected to a thiourea anion‐binding motif. The photogenerated metastable state of the motor‐based transporter exhibits up to 85% higher activity relative to its dark state, where the transport process itself is gradient‐driven (passive transport). Continuous light energy input is required to sustain the more active metastable nonequilibrium state. This work demonstrates application of light‐driven molecular motor to facilitate and regulate transmembrane anion transport. Our studies will be of guidance in the development of molecular motor‐based strategies that will advance artificial transporters toward dynamic light‐responsive systems with spatiotemporally controllable activity.

## Graphical abstract

A molecular motor functionalized with a thiourea anion‐binding motif mediates transmembrane chloride transport. Light irradiation enhances transport efficiency by populating a more active metastable isomer, which slowly equilibrates to the initial stable isomer. The transport enhancement appears to be associated with a change in self‐aggregation and membrane alteration upon isomerization, rather than the motor's rotational motion.

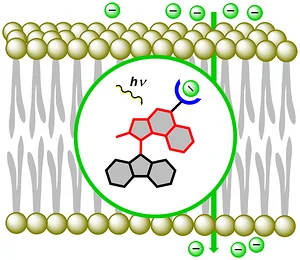

## 1 Introduction

Ion transport across biological membranes is fundamental to living systems, underpinning essential functions such as signal transduction, osmoregulation, and energy conversion [1]. This transport is tightly regulated by membrane‐embedded proteins acting as channels or carriers [2]. Inspired by these natural systems, various synthetic ion transporters have been developed over the past decades [3, 4, 5, 6, 7]. However, most synthetic systems lack the dynamic and responsive properties that are characteristic to proteins, leaving external control of ion transport a central challenge.

Light is an attractive stimulus to control transmembrane transport because of its high spatiotemporal precision, noninvasiveness, and tunable wavelength and intensity [8, 9]. Currently known light‐responsive bilayer transport systems are mostly based on bistable molecular photoswitches [10, 11, 12, 13, 14, 15, 16], requiring different wavelengths for toggling between active and inactive states, or on photocleavable protecting groups [17, 18, 19], only allowing activation. Furthermore, responsivity is usually achieved by targeting changes in binding affinity, while other factors such as the mobility in the membrane can be decisive in transport capability [20]. In contrast, biological regulation often relies on subtle membrane‐interaction and conformational changes sustained by continuous energy input, rather than the switching between stable resting states [21]. Adopting such features in artificial transport systems would provide a powerful strategy to precisely control activity.

Recent attempts toward this goal have led to transport systems based on molecular motors, [22, 23, 24] mechanically interlocked molecules [25, 26], and membrane‐anchored architectures [27, 28]. In particular, the groups of Giuseppone [22], Bao [23], and Zeng [24] have reported designs in which crown ethers were coupled with Feringa‐type light‐driven molecular motor [29, 30], giving modest enhancement in alkali metal cation transport efficiency upon continuous irradiation (Table S1). While these studies represent major conceptual advances, the underlying reasons for the measured increase in transport under illumination are not yet fully understood. Further, analogous motor‐based systems capable of mediating transport of anions have not been reported, despite the high importance of anionic species in biological systems [3].

Herein, we report light‐induced enhancement of chloride transport down a concentration gradient (i.e. passive transport) using Feringa‐type overcrowded alkene‐based molecular motor modified with a thiourea anion‐binding motif (Scheme 1). The earlier reported cation transport systems were based on a fast thioxanthene‐based molecular motor [22, 23, 24], having a theoretical rotation rate in the megaHertz regime [30]. In contrast, our design involves a much slower fluorene‐based motor with a maximum rotation rate of about 1.8 mHz [31]. We demonstrate an increase in transport efficiency under continuous 405 nm irradiation by generating a more active, metastable nonequilibrium state. Interestingly, a fast‐rotating analog of our transporter with a thioxanthene stator did not show increased transport activity. Our combined studies indicate that disaggregation effects and altered membrane properties are at the origin of the observed transport enhancement.

**SCHEME 1:**
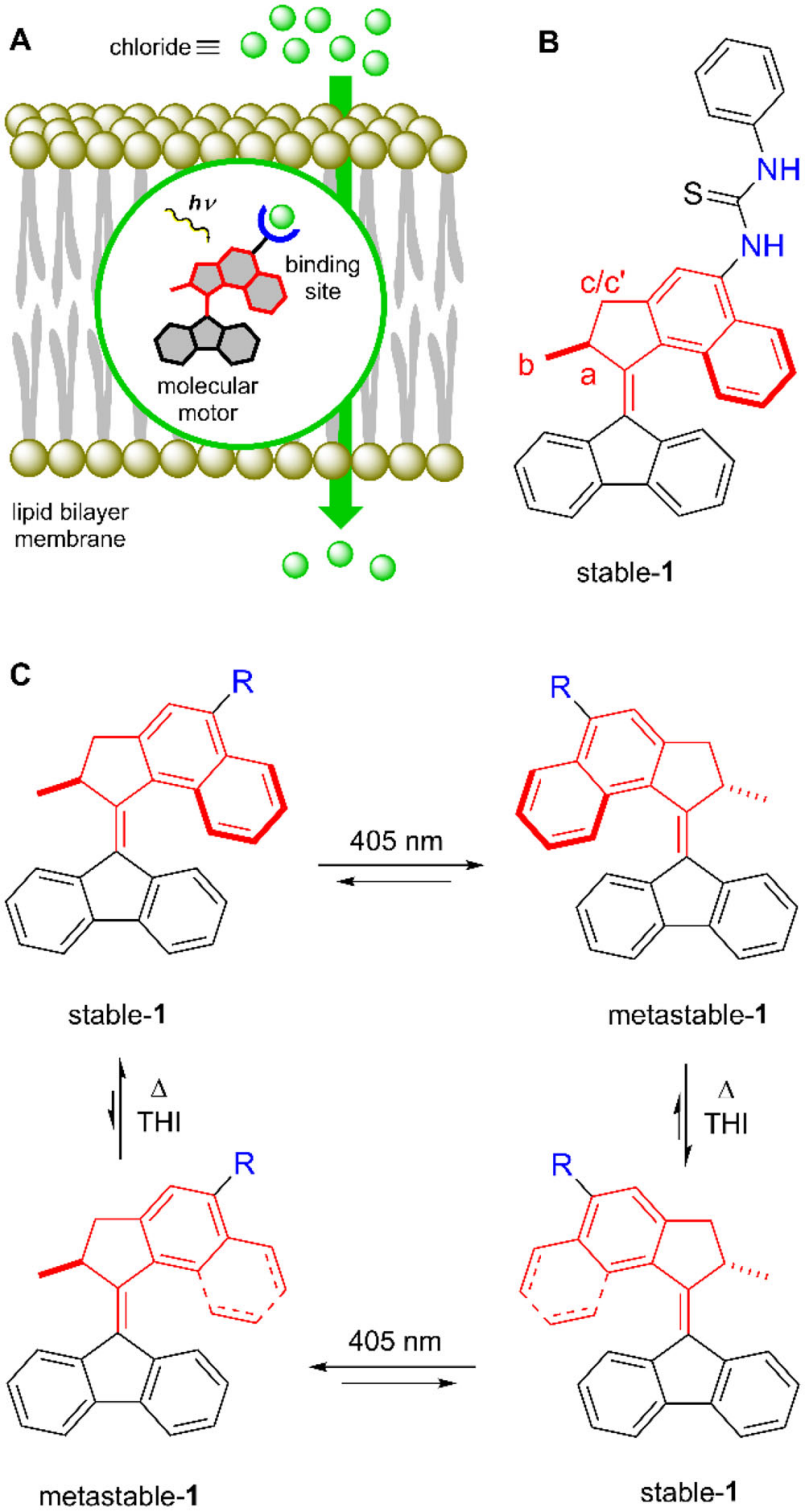
(A) Schematic representation of light‐enhanced chloride transport across a lipid bilayer membrane by thiourea‐appended molecular motor. (B) Chemical structure of molecular motor‐based transporter **1** and (C) its rotational cycle showing photoisomerization and thermal helix inversion (THI) steps. Please note that the drawings of stable‐**1** and of metastable‐**1** are identical structures shown from different viewpoints.

## 2 Results and Discussion

The desired molecular motor‐based transporter **1** was synthesized starting from a known bromo‐substituted derivative (Scheme S1) [32]. The phenyl thiourea substituent was introduced through a Buchwald–Hartwig amination, followed by reaction with phenyl isothiocyanate.

The photochemical and thermal isomerization behavior of **1** was first examined by UV–vis spectroscopy in MeCN, CHCl_3_, and MeOH. Different solvents were chosen to assess the influence of the medium on the half‐life of the photogenerated metastable state. Stable‐**1** displayed an absorption maximum at *λ* = 403 nm in all selected solvents. Irradiation near this maximum with 405 nm light at 20°C induced a bathochromic shift (*λ*_max_ = 403 → 430 nm; Figures 1A and S9–S11) with a clear isosbestic point at *λ* = 425 nm, consistent with unimolecular conversion to the metastable isomer. The samples were irradiated for 300 s, which was sufficient to reach the photostationary state (PSS). After irradiation, the decay in absorption at 480 nm was monitored over time and fitted to a first‐order kinetic model, yielding half‐lives for the metastable isomer (*t*_1/2_) of 98.8 ± 0.6 s in MeCN, 152.4 ± 2.5 s in CHCl_3_, and 266.1 ± 1.3 s in MeOH.

**FIGURE 1:**
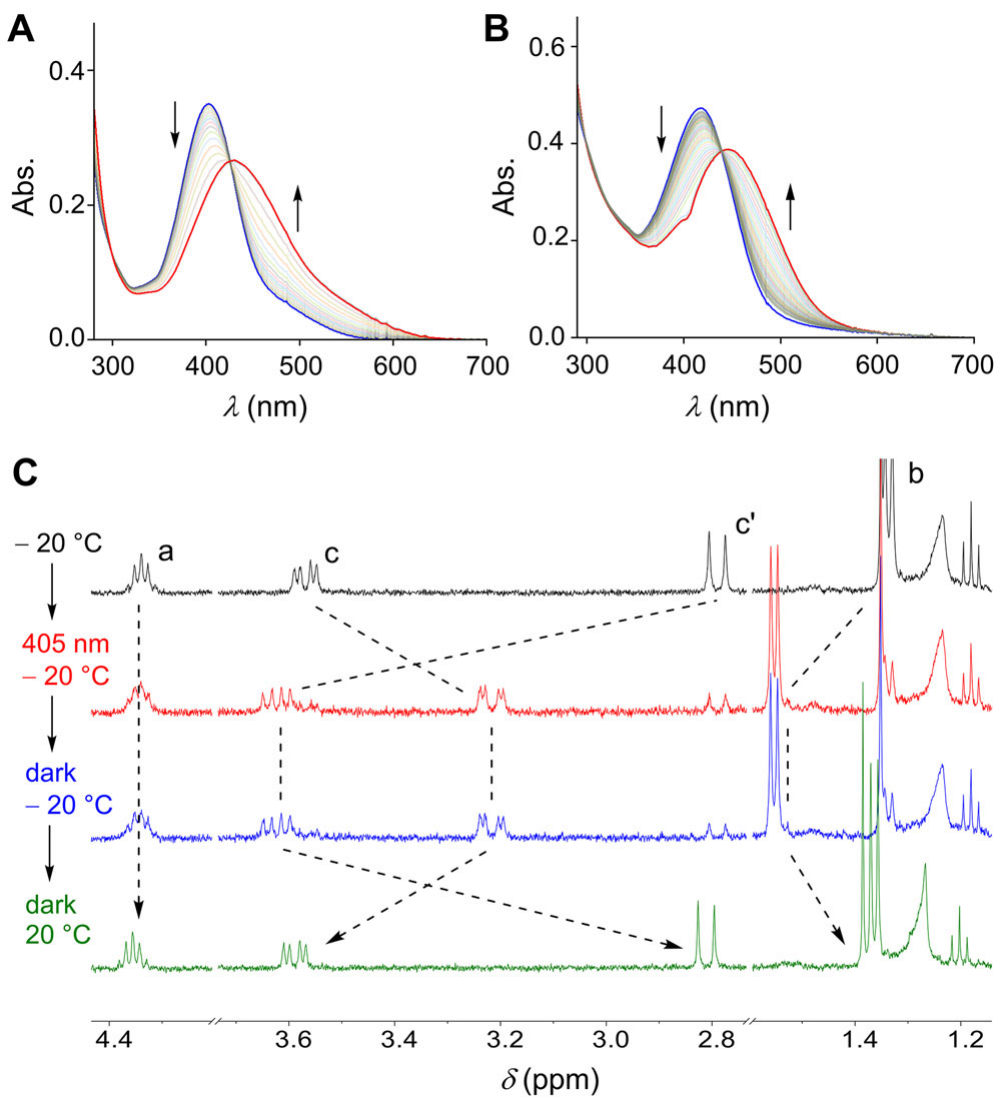
UV–vis spectra of **1** in acetonitrile (2.25 × 10^−5^ M) (A) and in POPC liposomes (5 mol%) (B) before and after irradiation with 405 nm light at 20°C. The samples were irradiated for 300 s and spectra were recorded at 30 s intervals. ^1^H NMR spectra of **1** (500 MHz, 1 mM in acetonitrile‐*d_3_*) before and after 405 nm irradiation at –20°C for 30 min., followed by standing in the dark, first at –20°C for 10 min, and subsequently at 20°C (C).

Upon incorporation into the bilayer of 1‐palmitoyl‐2‐oleoyl‐sn‐glycero‐3‐phosphocholine (POPC) liposomes (3, 5, and 7 mol% relative to lipid), stable‐**1** exhibited red‐shifted absorption compared to that in the organic solvents used (Figures 1B and S12–S14). Furthermore, this shift in absorption maximum became slightly larger with increased loading (*λ*_max_ = 416, 418, and 420 nm at 3, 5, and 7 mol%, respectively). This observation is consistent with previously reported bathochromic shifts for structurally related molecular motor conjugated with phospholipids, which was attributed to aggregate formation [33]. Irradiation of stable‐**1** in the POPC bilayer with 405 nm light produced a further bathochromic shift, where now the absorption maximum (*λ*_max_ = 446 nm) was essentially the same at the three loadings used. In all cases, an isosbestic point was maintained around *λ* = 440 nm and prolonged irradiation did not show any signs of degradation (Figure S15). After irradiation, the decay in absorbance was again followed over time, giving a half‐life for the metastable isomer of 212.2 ± 0.6, 232.9 ± 0.8, and 256.5 ± 1.2 s at 3, 5, and 7 mol% loading, respectively. This concentration dependence points to self‐aggregation within the membrane [34].

These photochemical and thermal isomerization processes were further monitored by ^1^H NMR spectroscopy in CD_3_CN. Because of higher concentration used compared to the UV–vis studies (i.e., Abs > 2), longer irradiation times were required to reach PSS_405_. Hence, to suppress thermal relaxation of the photogenerated metastable isomer during irradiation, the solution was cooled to −20°C. Upon irradiation with 405 nm light for 30 min, using an optical fiber guided into the NMR tube [35], a new set of signals corresponding to metastable‐**1** appeared (Figures 1C and S16). Relative integration of the two signal sets gave a PSS_405_ stable/metastable ratio of 36:64. Notably, after switching off the light source at −20°C, this ratio remained unchanged for at least 10 min, revealing negligible thermal isomerization under these conditions. Upon warming to 20°C, the metastable isomer thermally relaxed to the stable form quickly. The PSS ratio and thermal half‐life values are similar to those reported for molecular motors bearing a fluorenyl stator and a cyclopenta[a]naphthalene rotor [31, 36]. Thus, these results demonstrate that thiourea functionalization does not compromise the photochemical nor the thermal isomerization behavior of the molecular motor [37].

Next, the chloride binding properties of stable‐**1** were investigated by ^1^H NMR titration in CD_3_CN at 20°C. A prior dilution experiment (5.0–0.625 mM; Figure S17) showed concentration‐independent chemical shifts below 2.5 mM, indicating negligible self‐association. Incremental addition of [Bu_4_N]^+^[Cl]^−^ (0–31 equiv.) to stable‐**1** at 1.0 mM caused distinct downfield shifts of the thiourea NH and adjacent aromatic protons, indicative of chloride binding (Figure S18). The data were fitted to a 1:1 binding model using HypNMR [38], yielding an association constant (*K*_a_) of 1.9 × 10^3^ M^−^^1^ (Figure S19). Due to the short half‐life of metastable‐**1** at 20°C, its chloride binding affinity could not be quantified. The nearly identical thiourea NH chemical shift before and after irradiation, however, indicates that proton acidity is not significantly altered. Therefore, no major difference in binding affinity is expected between the isomers.

We then investigated the ability of compound **1** to mediate chloride transport across a lipid bilayer membrane. Fluorescent dye‐based assays (e.g., HPTS) [39] are frequently used to evaluate transmembrane ion transport properties, including those of molecular motors [22, 23, 24]. However, the results from such assays could potentially be influenced by the absorption of compound **1** at the dye excitation wavelengths (for HPTS: *λ*_ex_ = 403 and 454 nm), while in situ irradiation with 405 nm light to isomerize the molecular motor would excite (and possibly photobleach) the dye. We therefore conducted our transport studies using an ion‐selective electrode (ISE)‐based assay [40], and monitored transport under in situ irradiation by an osmotic response assay [41].

A cationophore‐coupled chloride efflux assay using a chloride‐selective electrode was first performed [42]. Here, large unilamellar POPC vesicles (200 nm) are used containing 300 mM of potassium chloride (KCl) internally and potassium gluconate (KGlu) solutions externally, both buffered at pH 7.2. Because the large, highly polar gluconate anion is membrane‐impermeable, chloride efflux under these conditions is limited by the buildup of either a membrane potential (in case of an electrogenic transport) or a pH gradient (in case of an electroneutral transport). Addition of the K^+^‐selective ionophore valinomycin dissipates membrane potentials [43], whereas addition of monensin eliminates pH gradients via K^+^/H^+^ antiport [44], thereby initiating chloride efflux in each case. The resulting increase in external chloride concentration is monitored in real time with the chloride‐selective electrode. Upon the addition of valinomycin (0.1 mol% relative to lipid) to liposomes containing stable‐**1** (5 mol% relative to lipid), significant chloride efflux was observed (Figures 2A and S20). Addition of monensin instead produced lower efflux, indicating that compound **1** operates preferentially via an electrogenic over an electroneutral mechanism. Therefore, all subsequent transport experiments were conducted in the presence of valinomycin.

**FIGURE 2:**
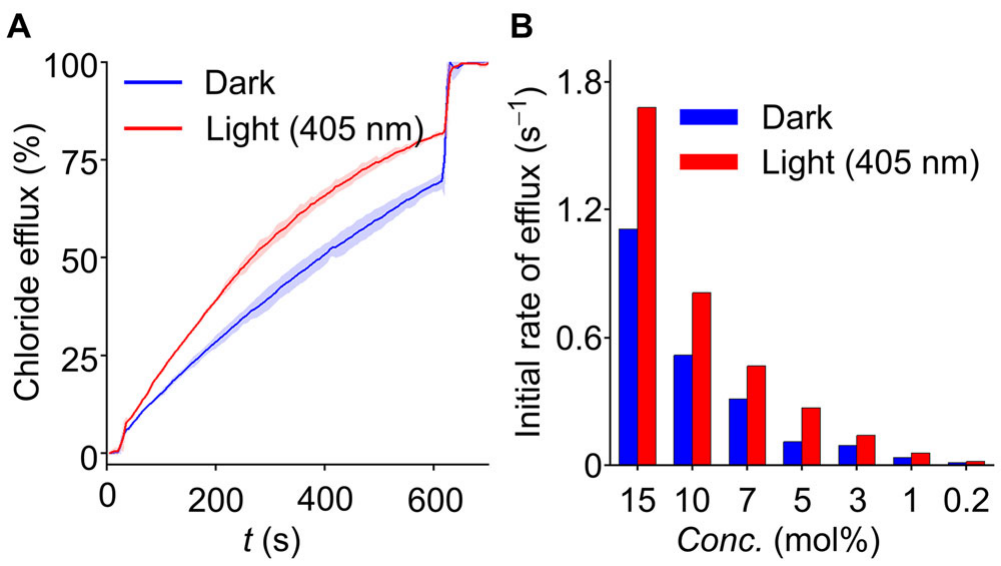
(A) Chloride efflux (5 mol%) against time facilitated by stable‐**1** (blue) and metastable‐**1** (red) (B) and initial rates of efflux by stable‐**1** (blue) and metastable‐**1** (red) at different loadings (0.2–15 mol%).

The effect of light on the activity of transporter **1** was then examined. To avoid optical interference at the electrode during 405 nm irradiation, liposomes containing stable‐**1** (5 mol%) were irradiated ex situ for 300 s to generate metastable‐**1**, after which transport measurements were initiated by adding valinomycin [42]. A fraction of the metastable form thermally relaxes back to the stable form during the measurement, given that the half‐life is approximately 4 min in the lipid bilayer. Nevertheless, significantly higher chloride efflux was observed for the irradiated sample compared to the initial sample containing stable‐**1** (Figure 2A), indicating that the photogenerated metastable‐**1** is a more active transporter. Control experiments without valinomycin showed no chloride efflux, either in the dark or after 405 nm irradiation, confirming that irradiation does not disrupt membrane integrity or induce leakage (Figure S21). Hill analysis of dose–response data gave half maximal effective concentrations (*EC*_50_) of 4.62 mol% for stable‐**1** and 2.59 mol% for the PSS_405_ mixture (Figures S22–S24). Hence, metastable‐**1** is at least 1.8 times more efficient than stable‐**1**. The difference was more pronounced in the initial rates of chloride efflux (*k*_ini_) as they are independent of the thermal isomerization occurring throughout the experiment (Figure 2B).

To monitor chloride transport in real time during irradiation, we used an osmotic response assay. Here, large unilamellar POPC vesicles (400 nm) with pre‐incorporated stable‐**1** (3–7 mol%) were also loaded with 300 mM KCl and contained external 300 mM KGlu solutions, buffered to pH 7.2. In this case, vesicle shrinkage caused by osmotic dehydration upon net KCl efflux—initiated by valinomycin addition—is detected by an increase in 90° light scattering intensity at 600 nm. During the experiments, the vesicles were irradiated with 405 nm light and kept in the dark at alternating 300 s intervals. Gratifyingly, irradiation resulted in an enhancement of chloride transport by up to 85% (at 3 mol% relative to lipid) relative to a sample kept in the dark (Figures 3B,C and S25). No transport was observed in the absence of compound **1** or valinomycin, both in the dark and upon 405 nm irradiation (Figures S27 and S28). Furthermore, a similar experiment using the aminated precursor to stable‐**1** did not show signs of transport (Figure S29). Finally, a nonswitchable, well‐known tren‐based tris(thiourea) transporter was used [45], which showed no light‐induced enhancement under otherwise identical conditions (Figure S30). These results show the necessity of the thiourea binding site for mediating chloride transport, and exclude leakage or increased permeation of the membrane by irradiation of the embedded compounds.

**FIGURE 3:**
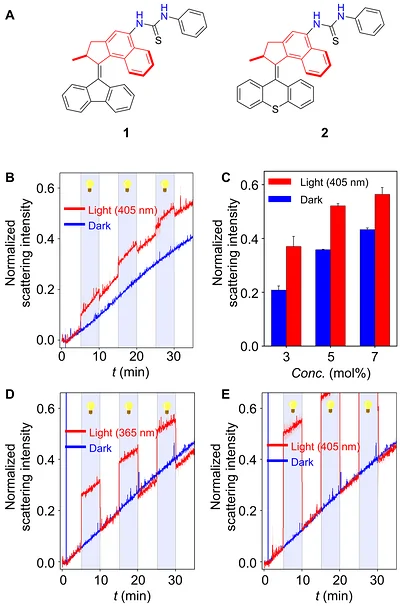
Structures of slow motor‐based transporter **1** and fast motor‐based transporter **2** (A). Normalized scattering intensity as a measure of chloride transport by **1** (5 mol%) in dark (blue) and under in situ irradiation using 405 nm light (red) (B). Column chart of normalized scattering intensities at 35 min for **1** at different loadings in the dark (blue) and upon in situ irradiation with 405 nm light (red) (C). Normalized scattering intensity as a measure of chloride transport by **2** (5 mol%) under in situ irradiation using 365 nm (D) and 405 nm light (E). Note that intensities are normalized to the values obtained after maximum efflux reached by prodigiosin and carbonyl cyanide 4‐(trifluoromethoxy)phenylhydrazone (FCCP) addition.

To assess whether the light‐induced enhancement of anion transport is associated with motor rotational motion, we synthesized a fast‐rotating analog (**2**; Figure 3A) featuring a thioxanthene instead of a fluorene stator. This motor type is known to complete a full rotation on a submicrosecond timescale [30]. Compound **2** was synthesized similarly to its slow‐rotating counterpart **1** (Scheme S2). The absorption of the thioxanthene‐based motor is slightly blue‐shifted compared to the fluorene‐based motor (e.g., *λ*_max_ (**2**) = 365 nm, see Figure S36). Therefore, 365 nm irradiation has typically been used to induce its photoisomerization [22, 23, 24]. In the osmotic response assay, stable‐**2** showed transport activity comparable to stable‐**1**. Interestingly, in situ irradiation, either at 365 or 405 nm light, gave large increases in scattering intensity (Figure 3D,E). However, these increases immediately disappeared upon cessation of light exposure and the scattering intensity of dark and irradiated samples (after the irradiation intervals) was virtually the same, meaning that transport by compound **2** was not enhanced by light irradiation. The observed increases during irradiation can mainly be attributed to emission by the molecular motor (Figures S31,S32, and S36). In addition, when this compound was tested using the HPTS assay [46], in situ irradiation at 365 or 405 nm did not enhance transport, while strongly increased fluorescence was observed during the irradiation intervals (Figures S33–S35). Notably, the fact that transport is not enhanced with analog **2** not only suggests that the increase we observed with the much slower‐rotating **1** does not relate to its rotational motion, but also strongly argues against possible photothermal effects.

Hence, we propose the following explanation for the light‐induced transport enhancement by slow‐rotating motor **1**. Structurally related molecular motors with a fluorenyl stator and cyclo‐penta[a]naphthalene rotor were reported to form small aggregates in lipid bilayers [33, 46]. The red‐shifted absorption of stable‐**1** in the bilayer compared to solution, as well as the concentration dependence of the absorption maximum and half‐life found in the liposome UV–vis studies (*vide supra*) point in the same direction. Population of the metastable isomer, characterized by a larger dihedral around the central double bond [36], could perturb the aggregation and therefore increase transporter mobility. Furthermore, this class of molecular motor was shown to induce membrane expansion upon irradiation [47], which is expected to also enhance transporter mobility due to increased membrane fluidity [48]. To investigate whether a change in membrane environment plays a role in the light‐induced enhancement of chloride transport by **1**, we repeated the osmotic response assay in POPC/cholesterol (85:15) vesicles, since cholesterol doping was previously shown to reduce photoinduced membrane remodeling by structurally‐related molecular motors [46]. Indeed, the light‐induced enhancement of chloride transport by **1** (5 mol%) was attenuated compared to POPC vesicles (Figure S26), which is consistent with a smaller effect on the membrane fluidity. Overall, isomerization of **1** may thus affect its own mobility as well as the surrounding membrane properties, together accounting for the observed light‐enhanced transport activity.

## 3 Conclusions

In summary, we functionalized a Feringa‐type molecular motor with a thiourea anion‐binding motif and demonstrated this compound to be capable of mediating (passive) chloride transport across the lipid bilayer. Its transport efficiency was enhanced by up to 85% under 405 nm irradiation, which populates the more active metastable state. Interestingly, a fast‐rotating structural analog did not show discernable enhancement of transport activity under continuous irradiation, indicating that the observed enhancement of transport activity—in our case—is not due to rotational motion of the molecular motor. It is rather ascribed to increased mobility by disaggregation and changes in the membrane properties. We anticipate that such mobility control will allow spatiotemporal immobilization of synthetic transporters in bilayer membranes, ultimately leading to a potential strategy for the development of light‐driven active transport systems. To conclude, this study extends the application of molecular motors to the transmembrane transport of anions and will guide the further design and analysis of molecular motor‐based transmembrane transport systems.

## Author Contributions

**Abhishek Mondal**: conceptualization, investigation, funding acquisition, writing – original draft. **Sander J. Wezenberg**: conceptualization, funding acquisition, writing – review and editing, supervision.

## Conflicts of Interest

The authors declare no conflicts of interest.

## Supporting information



The authors have cited additional references within the Supporting Information [49].**Supporting File**: anie74285‐sup‐0001‐SuppMat.pdf.

## Data Availability

The data that support the findings of this study are available in the Supporting Information of this article.

## References

[1] G. M. Cooper , The Cell: A Molecular Approach, 8th ed. (Oxford University Press, 2019), 45–80.

[2] G. R. Dubyak , “Ion Homeostasis, Channels, and Transporters: An Update on Cellular Mechanisms,” Advances in Physiology Education 28 (2004): 143–154, 10.1152/advan.00046.2004. 15545343

[3] J. T. Davis , P. A. Gale , and R. Quesada , “Advances in Anion Transport and Supramolecular Medicinal Chemistry,” Chemical Society Reviews 49 (2020): 6056–6086, 10.1039/C9CS00662A. 32692794

[4] S.‐P. Zheng , L.‐B. Huang , Z. Sun , and M. Barboiu , “Self‐Assembled Artificial Ion‐Channels Toward Natural Selection of Functions,” Angewandte Chemie International Edition 60 (2021): 566–597, 10.1002/anie.201915287. 32212308

[5] A. Mondal , M. Ahmad , D. Mondal , and P. Talukdar , “Progress and Prospects Toward Supramolecular Bioactive Ion Transporters,” Chemical Communications 59 (2023): 1917–1938, 10.1039/D2CC06761G. 36691926

[6] D. Zhang , W. Chang , J. Shen , and H. Zeng , “Aromatic Foldamer‐Derived Transmembrane Transporters,” Chemical Communications 60 (2024): 13468–13491, 10.1039/D4CC04388J. 39466066

[7] T. Yan and J. Liu , “Transmembrane Ion Channels: From Natural to Artificial Systems,” Angewandte Chemie International Edition 64 (2025): e202416200, 10.1002/anie.202416200. 39545394

[8] J. de Jong , J. E. Bos , and S. J. Wezenberg , “Stimulus‐Controlled Anion Binding and Transport by Synthetic Receptors” Chemical Reviews 123 (2023): 8530–8574. 10.1021/acs.chemrev.3c00039 PMC10347431 37342028

[9] M. Ahmad , S. A. Gartland , and M. J. Langton , “Photo‐ and Redox‐Regulated Transmembrane Ion Transporters,” Angewandte Chemie International Edition 62 (2023): e202308842, 10.1002/anie.202308842. 37478126

[10] Y. R. Choi , G. C. Kim , H.‐G. Jeon , J. Park , W. Namkung , and K.‐S. Jeong , “Azobenzene‐Based Chloride Transporters With Light‐Controllable Activities,” Chemical Communications 50 (2014): 15305–15308, 10.1039/C4CC07560A. 25350406

[11] A. Kerckhoffs and M. J. Langton , “Reversible Photo‐Control Over Transmembrane Anion Transport Using Visible‐Light Responsive Supramolecular Carriers,” Chemical Science 11 (2020): 6325–6331, 10.1039/D0SC02745F. PMC7472928 32953027

[12] M. Ahmad , S. Metya , A. Das , and P. Talukdar , “A Sandwich Azobenzene–Diamide Dimer for Photoregulated Chloride Transport,” Chemistry‐A European Journal 26 (2020): 8703–8708, 10.1002/chem.202000400. 32129531

[13] S. J. Wezenberg , L.‐J. Chen , J. E. Bos , et al., “Photomodulation of Transmembrane Transport and Potential by Stiff‐Stilbene Based Bis(thio)Ureas,” Journal of the American Chemical Society 144 (2022): 331–338, 10.1021/jacs.1c10034. PMC8759083 34932344

[14] T. G. Johnson , A. Sadeghi‐Kelishadi , and M. J. Langton , “A Photo‐Responsive Transmembrane Anion Transporter Relay,” Journal of the American Chemical Society 144 (2022): 10455–10461, 10.1021/jacs.2c02612. PMC9204766 35652660

[15] N. Duindam , J. E. Bos , F. van Nifterik , and S. J. Wezenberg , “Hemi‐Indigiosin: A pH and Red‐Light Responsive Transmembrane HCl Transporter,” Angewandte Chemie International Edition 64 (2025): e15930, 10.1002/anie.202515930. PMC12707358 41169028

[16] M. Ahmad , S. Sarkar , R. Bhogade , et al., “Dynamic Regulation of Ion Transport Through a Bis(1,3‐propanediol)‐Based Channel via Allosteric Azobenzene Photoswitching,” Nanoscale 17 (2025): 3145–3151, 10.1039/D4NR01711K. 39688448

[17] S. B. Salunke , J. A. Malla , and P. Talukdar , “Phototriggered Release of a Transmembrane Chloride Carrier From an *o*‐Nitrobenzyl‐Linked Procarrier,” Angewandte Chemie International Edition 58 (2019): 5354–5358, 10.1002/anie.201900869. 30758120

[18] M. Ahmad , N. J. Roy , A. Singh , et al., “Photocontrolled Activation of Doubly *o*‐nitrobenzyl‐Protected Small Molecule Benzimidazoles Leads to Cancer Cell Death,” Chemical Science 14 (2023): 8897–8904, 10.1039/D3SC01786A. PMC10445434 37621434

[19] M. Ahmad , R. M. Devereux , A. J. Russell , and M. J. Langton , “Near‐Infrared Triggered Anion Transport Induces Cancer Cell Death,” Angewandte Chemie International Edition 65 (2026): e23734, 10.1002/anie.202523734. PMC12851001 41388719

[20] J. E. Bos , M. A. Siegler , and S. J. Wezenberg , “Activity Control of a Synthetic Transporter by Photodynamic Modulation of Membrane Mobility and Incorporation,” Journal of the American Chemical Society 146 (2024): 31085–31093, 10.1021/jacs.4c10952. PMC11565646 39485737

[21] T. S. Tillman and M. Cascio , “Effects of Membrane Lipids on Ion Channel Structure and Function,” Cell Biochemistry and Biophysics 38 (2003): 161–190, 10.1385/CBB:38:2:161. 12777713

[22] W.‐Z. Wang , L.‐B. Huang , S.‐P. Zheng , et al., “Light‐Driven Molecular Motors Boost the Selective Transport of Alkali Metal Ions Through Phospholipid Bilayers,” Journal of the American Chemical Society 143 (2021): 15653–15660, 10.1021/jacs.1c05750. 34520204

[23] H. Yang , J. Yi , S. Pang , et al., “A Light‐Driven Molecular Machine Controls K^+^ Channel Transport and Induces Cancer Cell Apoptosis,” Angewandte Chemie International Edition 61 (2022): e202204605, 10.1002/anie.202204605. 35442566

[24] S. Deng , Z. Li , L. Yuan , J. Shen , and H. Zeng , “Light‐Powered Propeller‐Like Transporter for Boosted Transmembrane Ion Transport,” Nano Letters 24 (2024): 10750–10758, 10.1021/acs.nanolett.4c01884. 39177063

[25] S. Chen , Y. Wang , T. Nie , et al., “An Artificial Molecular Shuttle Operates in Lipid Bilayers for Ion Transport,” Journal of the American Chemical Society 140 (2018): 17992–17998, 10.1021/jacs.8b09580. 30445811

[26] C. Wang , S. Wang , H. Yang , et al., “A Light‐Operated Molecular Cable Car for Gated Ion Transport,” Angewandte Chemie International Edition 60 (2021): 14836–14840, 10.1002/anie.202102838. 33843130

[27] R. Ye , C. Ren , J. Shen , et al., “Molecular Ion Fishers as Highly Active and Exceptionally Selective K^+^ Transporters,” Journal of the American Chemical Society 141 (2019): 9788–9792, 10.1021/jacs.9b04096. 31184884

[28] C. Ren , F. Chen , R. Ye , et al., “Molecular Swings as Highly Active Ion Transporters,” Angewandte Chemie International Edition 58 (2019): 8034–8038, 10.1002/anie.201901833. 30983075

[29] N. Koumura , E. M. Geertsema , M. B. van Gelder , A. Meetsma , and B. L. Feringa , “Second Generation Light‐Driven Molecular Motors. Unidirectional Rotation Controlled by a Single Stereogenic Center With Near‐Perfect Photoequilibria and Acceleration of the Speed of Rotation by Structural Modification,” Journal of the American Chemical Society 124 (2002): 5037–5051, 10.1021/ja012499i. 11982368

[30] G. M. Klok , N. Boyle , M. T. Pryce , A. Meetsma , W. R. Browne , and B. L. Feringa , “MHz Unidirectional Rotation of Molecular Rotary Motors,” Journal of the American Chemical Society 130 (2008): 10484–10485, 10.1021/ja8037245. 18636709

[31] J. Vicario , A. Meetsma , and B. L. Feringa , “Controlling the Speed of Rotation in Molecular Motors. Dramatic Acceleration of the Rotary Motion by Structural Modification,” Chemical Communications 41 (2005): 5910–5912, 10.1039/b507264f. 16317472

[32] J. Kaleta , J. Chen , G. Bastien , et al., “Surface Inclusion of Unidirectional Molecular Motors in Hexagonal Tris(*o*‐phenylene)Cyclotriphosphazene,” Journal of the American Chemical Society 139 (2017): 10486–10498, 10.1021/jacs.7b05404. 28654753

[33] A. Guinart , D. Doellerer , Y. Qutbuddin , et al., “Photoswitchable Molecular Motor Phospholipid: Synthesis, Characterization, and Integration Into Lipid Vesicles,” Langmuir 41 (2025): 3961–3970, 10.1021/acs.langmuir.4c04173. PMC11841041 39900533

[34] H. Qian , Y.‐Y. Wang , D.‐S. Guo , and I. Aprahamian , “Controlling the Isomerization Rate of an Azo‐BF_2_ Switch Using Aggregation,” Journal of the American Chemical Society 139 (2017): 1037–1040, 10.1021/jacs.6b10982. 28072532

[35] C. Feldmeier , H. Bartling , E. Riedle , and R. M. Gschwind , “LED Based NMR Illumination Device for Mechanistic Studies on Photochemical Reactions—Versatile and Simple, yet Surprisingly Powerful,” Journal of Magnetic Resonance 232 (2013): 39–44, 10.1016/j.jmr.2013.04.011. 23685874

[36] J. Vicario , M. Walko , A. Meetsma , and B. L. Feringa , “Fine Tuning of the Rotary Motion by Structural Modification in Light‐Driven Unidirectional Molecular Motors,” Journal of the American Chemical Society 128 (2006): 5127–5135, 10.1021/ja058303m. 16608348

[37] As the UV–vis spectral changes observed upon irradiation of stable‐**1** in the bilayer closely resemble those in organic solvent (MeCN), a similar PSS ratio is expected.

[38] C. Frassineti , S. Ghelli , P. Gans , A. Sabatini , M. S. Moruzzi , and A. Vacca , “Nuclear Magnetic Resonance as a Tool for Determining Protonation Constants of Natural Polyprotic Bases in Solution,” Analytical Biochemistry 231 (1995): 374–382, 10.1006/abio.1995.9984. 8594988

[39] A. M. Gilchrist , P. Wang , I. Carreira‐Barral , et al., “Supramolecular Methods: The 8‐Hydroxypyrene‐1,3,6‐trisulfonic Acid (HPTS) Transport Assay,” Supramolecular Chemistry 33 (2021): 325–344, 10.1080/10610278.2021.1999956.

[40] A. V. Koulov , T. N. Lambert , R. Shukla , et al., “Chloride Transport across Vesicle and Cell Membranes by Steroid‐Based Receptors,” Angewandte Chemie International Edition 42 (2003): 4931–4933, 10.1002/anie.200351957. 14579442

[41] R. B. Stockbridge , H.‐H. Lim , R. Otten , et al., “Fluoride Resistance and Transport by Riboswitch‐Controlled CLC Antiporters,” Proceedings National Academy of Science USA 109 (2012): 15289–15294, 10.1073/pnas.1210896109. PMC3458365 22949689

[42] X. Wu , L. W. Judd , E. N. W. Howe , et al., “Nonprotonophoric Electrogenic Cl^−^ Transport Mediated by Valinomycin‐Like Carriers,” Chem 1 (2016): 127–146, 10.1016/j.chempr.2016.04.002.

[43] B. C. Pressman , “Biological Applications of Ionophores,” Annual Review of Biochemistry 45 (1976): 501–530, 10.1146/annurev.bi.45.070176.002441. 786156

[44] H. H. Mollenhauer , D. James Morré , and L. D. Rowe , “Alteration of Intracellular Traffic by Monensin; Mechanism, Specificity and Relationship to Toxicity,” Biochim Biophys Acta Rev Biomembr 1031 (1990): 225–246, 10.1016/0304-4157(90)90008-Z. PMC7148783 2160275

[45] L. A. Jowett , E. N. W. Howe , X. Wu , N. Busschaert , and P. A. Gale , “New Insights Into the Anion Transport Selectivity and Mechanism of Tren‐Based Tris‐(thio)ureas,” Chemistry‐A European Journal 24 (2018): 10475–10487, 10.1002/chem.201801463. 29786913

[46] The HPTS assay was not used for compound **1** due to its strong absorption at 403 and 450 nm (Figure S36), directly overlapping with the HPTS excitation wavelengths (403/454 nm). For compound **2**, the HPTS assay showed pronounced fluorescence increases during irradiation (Figures S33–S35), likely caused by direct excitation and emission of this compound, interfering with the HPTS readout.

[47] Y. Qutbuddin , A. Guinart , S. Gavrilović , K. Al Nahas , B. L. Feringa , and P. Schwille , “Light‐Activated Synthetic Rotary Motors in Lipid Membranes Induce Shape Changes through Membrane Expansion,” Advanced Materials 36 (2024): 2311176, 10.1002/adma.202311176. 38215457

[48] J. E. Bos , N. Duindam , T. J. F. Kock , M. A. Siegler , and S. J. Wezenberg , “Control of Bilayer Transport Through a Photoswitchable Membrane‐Stiffening Agent,” Angewandte Chemie International Edition 64 (2025): e202420232, 10.1002/anie.202420232. 39661481

[49] J. Sheng , C. L. F. van Beek , C. N. Stindt , et al., “General Strategy for Boosting the Performance of Speed‐Tunable Rotary Molecular Motors With Visible Light,” Science Advances 11 (2025): eadr9326, 10.1126/sciadv.adr9326. PMC11838004 39970219

