# Supplementary material for: Light‐Enhanced Chloride Transport by Thiourea‐Appended Molecular Motor
Source: Angew Chem Int Ed Engl. 2026 Aug 22;65(41):e4755839. doi: 10.1002/anie.4755839 (PMC13638100; doi:10.1002/anie.4755839)
Supplement: Supplementary file 1 [file ANIE-65-e4755839-s001.pdf]

SUPPORTING INFORMATION

Light-Enhanced Chloride Transport by Thiourea-Appended Molecular Motor

Abhishek Mondal and Sander J. Wezenberg*

Leiden Institute of Chemistry, Leiden University,

Einsteinweg 55, 2333 CC Leiden, The Netherlands

Email: s.j.wezenberg@lic.leidenuniv.nl

Table of Contents

Experimental section S2

^{1}H and ^{13}C NMR spectra of new compounds S8

UV-Vis photoisomerization studies S16

^{1}H NMR titrations and data fitting S20

Transmembrane transport experiments S25

References S45

Experimental section

General methods and materials: THF, toluene, and acetonitrile were dried using MBraun and Innovative Technology solvent purification systems. The degassing of solvents was carried out by purging with N_2 for 30 min. Compounds **3** and **5** were prepared according to procedures reported in the literature.[1] All other chemicals were commercial products and were used as received. Melting points (m.p.) were determined using a Büchi-M560 melting point apparatus. ^{1}H and ^{13}C NMR spectra were recorded on Varian Mercury-Plus 400, Bruker AV 400, and Bruker 500 Ultra Shield instruments at 298 K unless indicated otherwise. Chemical shifts (δ) are denoted in parts per million (ppm) relative to residual protiated solvent (DMSO-*d₆*: for ^{1}H detection, δ = 2.50 ppm; for ^{13}C detection, δ = 39.52 ppm; $CDCl_3$: for ^{1}H detection, δ = 7.26 ppm; for ^{13}C detection, δ = 77.16 ppm; acetonitrile-d_3: for ^{1}H detection, δ = 1.94 ppm; for ^{13}C detection, δ = 118.26 ppm). The splitting pattern of peaks is designated as follows: s (singlet), d (doublet), dd (doublet of doublets), t (triplet), q (quartet), p (quintet), h (septet), m (multiplet), and br (broad). High-resolution mass spectrometry (ESI-MS) was performed on a LTQ Orbitrap XL spectrometer with ESI ionization. IR spectra were recorded on a Perkin-Elmer Spectrum Two FT-IR spectrometer. The intensity of bands (ν = cm^{-1}) is assigned as follows: s (strong), m (medium), w (weak), br (broad), and sh (shoulder). UV-Vis spectra were recorded on an Agilent Cary 8454 spectrometer in a 1 cm or 1 mm quartz cuvette. Fluorescence measurements were performed using a JASCO FP-8500 spectrofluorometer equipped with a stirrer plate and a temperature controller. Irradiation of NMR and UV-Vis samples was carried out at 20 °C using a Thorlab model M365L2 high-power LED (360 mW) and a Thorlab model M405LP1 high-power LED (1200 mW) positioned at a distance of 2 cm from the sample, except noted otherwise. Polycarbonate membrane (200 nm and 400 nm pore size), and mini extruder used for vesicle preparation were obtained from Avanti Polar Lipids.

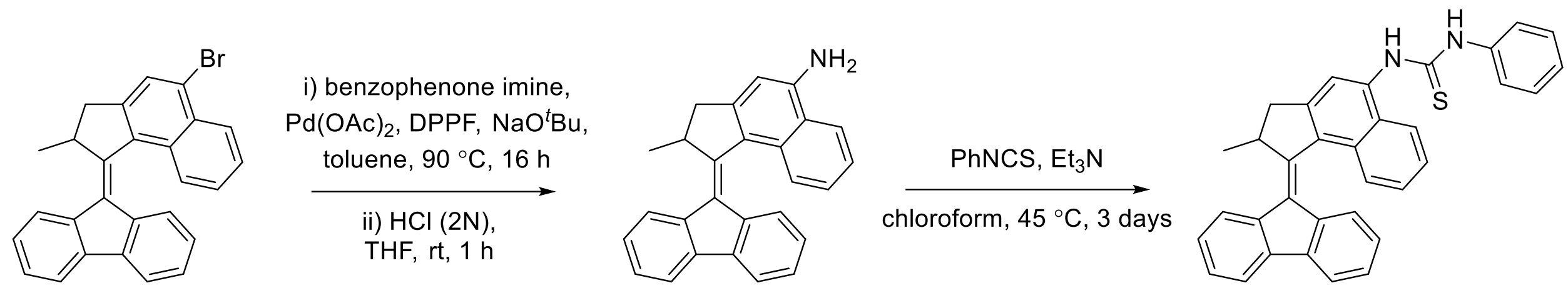

3 **4** (68%) **1** (60%)

Scheme S1. Synthesis of compound **1**.

1-(9*H*-fluoren-9-ylidene)-2-methyl-2,3-dihydro-1*H*-cyclopentanaphthalen-5-amine [4]: Compound **3** (0.11 g, 0.26 mmol), palladium (II) acetate (0.0059 g, 0.026 mmol), DPPF (0.018 g, 0.033 mmol), and sodium *tert*-butoxide (0.027 g, 0.29 mmol) were placed in a Schlenk tube and brought under N_2 via three vacuum/N_2 cycles. Then, dry and degassed toluene (1 mL) was added to the reaction mixture, followed by benzophenone imine (0.052 mL, 0.33 mmol). The mixture was stirred at 90 °C for 16 h using an oil bath, cooled to rt, and diluted with water (5 mL). The aqueous layer was extracted with $CHCl_3$ (3 × 15 mL). The combined organic layers were dried over Na_2SO_4 and concentrated. Purification by column chromatography (SiO_2, CH_2Cl_2) afforded the imine intermediate as a yellow solid. Subsequently, this imine intermediate was redissolved in THF (2 mL), and a 2 N aqueous HCl solution (0.5 mL) was added. The mixture was stirred for 1 h at rt, diluted with sat. aq. $NaHCO_3$ (5 mL) and extracted with CH_2Cl_2 (3 × 15 mL). The combined organic layers were dried over Na_2SO_4 and concentrated. Purification by column chromatography (SiO_2; Et_2O/Pentane 1:2) afforded **4** (0.064 g, 68%) as an orange solid. R_f = 0.15 (SiO_2; Et_2O/Pentane 1:3); **m.p.** 182 – 185 °C; **^{1}H NMR (400 MHz, $CDCl_3$):** δ 8.04 – 7.95 (m, 1H), 7.92 – 7.83 (m, 2H), 7.79 (dt, *J* = 7.6, 1.0 Hz, 1H), 7.74 (dt, *J* = 8.3, 0.9 Hz, 1H), 7.44 (ddd, *J* = 8.5, 6.8, 1.4 Hz, 1H), 7.41 – 7.36 (m, 2H), 7.32 (ddd, *J* = 8.2, 6.8, 1.2 Hz, 1H), 7.20 (td, *J* = 7.4, 1.1 Hz, 1H), 6.87 (s, 1H), 6.86 – 6.81 (m, 1H), 6.74 (dt, *J* = 7.9, 0.9 Hz, 1H), 4.54 (s, 2H), 4.35 – 4.24

(m, 1H), 3.48 (dd, J = 15.0, 5.7 Hz, 1H), 2.66 (d, J = 15.1 Hz, 1H), 1.40 (d, J = 6.7 Hz, 3H) ppm; **$^{13}C\{^1 H\}$ NMR (101 MHz, $CDCl_3$):** δ 152.6, 149.8, 145.4, 140.1, 139.5, 139.0, 137.4, 130.8, 128.4, 127.3, 127.2, 126.9, 126.7, 126.1, 126.0, 125.8, 125.6, 124.4, 123.7, 122.2, 121.3, 119.7, 118.9, 107.4, 45.0, 42.1, 21.1 ppm; **IR (ATR):** ν = 3387 (br, m), 2956 (m), 2922 (m), 2852 (m), 2360 (w), 1615 (m), 1587 (m), 1570 (m), 1552 (m), 1519 (m), 1488 (m), 1445 (m), 1349 (m), 1338 (m), 1307 (m), 1271 (m), 1242 (m), 1224 (m), 1171 (m), 1024 (m), 946 (w), 804 (w), 765 (w), 730 (m), 706 (m), 691 (m), 669 (m), 649 (m), 605 (w) cm^{-1}; **HR-ESI-MS:** ***m/z* (%):** 360.1714 ([M + H]$^+$, calcd for $C_{27}H_{22}N^+$: 360.1747).

1-(1-(9*H*-fluoren-9-ylidene)-2-methyl-2,3-dihydro-1*H*-cyclopentanaphthalen-5-yl)-3-phenylthiourea [1]: Compound **4** (0.023 g, 0.064 mmol) was dissolved in dry and degassed $CHCl_3$ (1 mL) under N_2 atmosphere and Et_3N (0.01 mL, 0.07 mmol) was added to the solution. After 30 min, phenyl isothiocyanate (0.030 mL, 0.26 mmol) was added whilst stirring the solution. The mixture was then stirred for 3 days at 45 °C. The solvent was evaporated and purification by column chromatography (SiO_2; Et_2O/Pentane 1:4) afforded compound **1** (0.019 g, 60%) as a yellow solid. R_f = 0.10 (SiO_2; Et_2O/Pentane 1:3); **m.p.** 150 – 152 °C; **^{1}H NMR (400 MHz, DMSO-*d*$_6$):** δ 9.80 (s, 1H), 9.66 (s, 1H), 8.68 (dt, J = 8.5, 0.9 Hz, 1H), 8.52 – 8.45 (m, 1H), 8.43 – 8.36 (m, 1H), 8.31 (d, J = 7.5 Hz, 2H), 8.25 – 8.16 (m, 1H), 8.07 – 7.94 (m, 3H), 7.90 – 7.86 (m, 2H), 7.85 – 7.75 (m, 3H), 7.69 (td, J = 7.4, 1.1 Hz, 1H), 7.61 (t, J = 7.4 Hz, 1H), 7.23 (dd, J = 7.2, 1.1 Hz, 1H), 7.16 (d, J = 7.9 Hz, 1H), 4.86 – 4.75 (m, 1H), 4.07 (dd, J = 15.3, 5.7 Hz, 1H), 3.30 – 3.27 (m, 1H), 1.83 (d, J = 6.7 Hz, 3H) ppm; **$^{13}C\{^1 H\}$ NMR (101 MHz, DMSO-*d*$_6$):** δ 215.8, 192.4, 161.2, 158.1, 150.5, 150.2, 150.1, 150.0, 147.6, 147.4, 145.5, 144.7, 141.0, 140.8, 139.6, 139.1, 139.0, 138.1, 137.7, 137.7, 137.6, 137.3, 136.3, 136.3, 136.2, 135.8, 135.4, 134.7, 134.5, 133.7,

130.2, 129.6, 55.9, 52.1, 29.2 ppm; **IR (ATR):** ν = 3151 (w), 2954 (w), 2922 (w), 2360 (w), 1586 (w), 1527 (m), 1488 (m), 1447 (w), 1435 (w), 1353 (w), 1307 (w), 1241 (m), 1205 (w), 1025 (w), 946 (w), 878 (w), 783 (w), 764 (m), 753 (m), 742 (m), 732 (m), 712 (w), 693 (m), 668 (w), 649 (w), 605 (w); **HR-ESI-MS:** ***m/z* (%):** 495.1885 ($[M + H]^+$, calcd for $C_{34}H_{27}N_2S^+$: 495.1889).

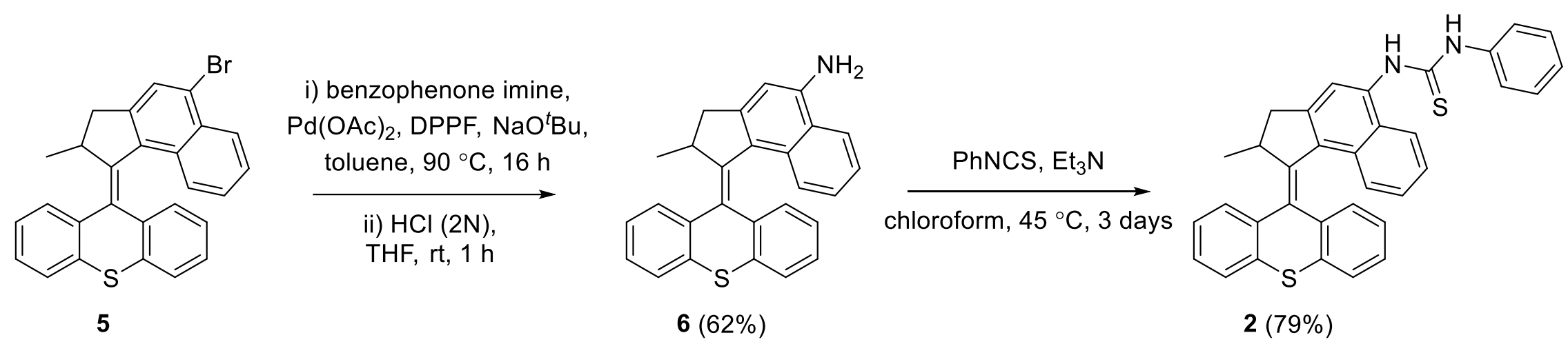

Scheme S2. Synthesis of compound **2**.

2-methyl-1-(9*H*-thioxanthen-9-ylidene)-2,3-dihydro-1*H*-cyclopentanaphthalen-5-amine [6]: Compound **5** (0.050 g, 0.11 mmol), palladium (II) acetate (0.0025 g, 0.011 mmol), DPPF (0.0077 g, 0.014 mmol), and sodium *tert*-butoxide (0.012 g, 0.12 mmol) were placed in a Schlenk tube and brought under N_2 via three vacuum/N_2 cycles. Then, dry and degassed toluene (1 mL) was added to the reaction mixture followed by benzophenone imine (0.023 mL, 0.14 mmol). The mixture was stirred at 90 °C for 16 h using an oil bath, cooled to rt, and diluted with water (5 mL). The aqueous layer was extracted with $CHCl_3$ (3 × 15 mL). The combined organic layers were dried over Na_2SO_4 and concentrated. Purification by column chromatography (SiO_2, CH_2Cl_2) afforded the imine intermediate as a yellow solid. Subsequently, this imine intermediate was redissolved in THF (2 mL), and a 2 N aqueous HCl solution (0.5 mL) was added. The mixture was stirred for 1 h at rt, diluted with sat. aq. $NaHCO_3$ (5 mL) and extracted with CH_2Cl_2 (3 × 15 mL). The combined organic layers were dried over Na_2SO_4 and concentrated. Purification by column chromatography (SiO_2; Et_2O/Pentane 1:4) afforded **6** (0.027 g, 62%) as an orange solid. R_f = 0.20 (SiO_2;

Et_2O/Pentane 1:3); **m.p.** 201 – 203 °C; **^{1}H NMR (500 MHz, $CDCl_3$):** δ 7.79 (dd, *J* = 7.8, 1.4 Hz, 1H), 7.65 (dt, *J* = 8.4, 0.9 Hz, 1H), 7.58 (ddd, *J* = 14.7, 7.7, 1.3 Hz, 2H), 7.31 (td, *J* = 7.5, 1.3 Hz, 1H), 7.23 – 7.12 (m, 2H), 6.98 (td, *J* = 7.6, 1.4 Hz, 1H), 6.89 (dd, *J* = 8.5, 1.3 Hz, 1H), 6.82 – 6.76 (m, 2H), 6.74 (s, 1H), 6.63 (td, *J* = 7.5, 1.3 Hz, 1H), 4.30 – 4.19 (m, 3H), 3.56 (dd, *J* = 15.4, 6.3 Hz, 1H), 2.52 (d, *J* = 15.4 Hz, 1H), 0.77 (d, *J* = 6.9 Hz, 3H) ppm; **^{13}C{^{1} H} NMR (126 MHz, $CDCl_3$):** δ 147.4, 146.5, 144.1, 141.1, 138.6, 135.9, 135.6, 129.7, 128.8, 127.9, 127.8, 127.5, 127.1, 126.3, 126.3, 126.2, 126.0, 125.9, 125.7, 124.9, 123.3, 123.0, 120.4, 107.3, 39.9, 37.7, 19.8. ppm; **IR (ATR):** ν = 3448 (w), 3366 (w), 3158 (w), 2954 (m), 2921 (m), 2860 (w), 2360 (w), 1622 (w), 1581 (m), 1563 (m), 1521 (m), 1487 (m), 1452 (m), 1435 (m), 1412 (w), 1356 (w), 1307 (w), 1260 (m), 1241 (m), 1163 (w), 1029 (m), 946 (w), 904 (w), 863 (w), 802 (w), 764 (m), 754 (m), 743 (m), 712 (m), 693 (m), 649 (m), 605 (m); **HR-ESI-MS: *m/z* (%):** 392.1436 ($[M + H]^+$, calcd for $C_{27}H_{22}NS^+$: 392.1467).

1-(2-methyl-1-(9*H*-thioxanthen-9-ylidene)-2,3-dihydro-1*H*-cyclopentanaphthalen-5-yl)-3-phenylthiourea [2]: Compound **6** (0.024 g, 0.061 mmol) was dissolved in dry and degassed $CHCl_3$ (2 mL) under N_2 atmosphere, and Et_3N (0.010 mL, 0.067 mmol) was added to the solution. After 30 min, phenyl isothiocyanate (0.030 mL, 0.25 mmol) was added whilst stirring the solution. The mixture was then stirred for 3 days at 45 °C. The solvent was evaporated, and purification by column chromatography (SiO_2; Et_2O/Pentane 1:4) afforded compound **2** (0.025 g, 79%) as a yellow solid. R_f = 0.10 (SiO_2; Et_2O/Pentane 1:3); **m.p.** 199 – 202 °C; **^{1}H NMR (500 MHz, DMSO-*d*$_6$):** δ 9.91 (s, 1H), 9.81 (s, 1H), 7.97 (d, *J* = 9.3 Hz, 1H), 7.87 (d, *J* = 8.5 Hz, 1H), 7.68 (d, *J* = 7.8 Hz, 2H), 7.63 (s, 1H), 7.47 (d, *J* = 7.3 Hz, 2H), 7.45 – 7.40 (m, 1H), 7.34 – 7.28 (m, 3H), 7.26 (m, 1H), 7.16 – 7.06 (m, 2H), 6.79 (s, 2H), 6.70 (s, 2H), 4.30 (m, 1H), 3.68 (dd, *J* = 15.8, 6.3 Hz, 1H),

2.60 (d, J = 15.9 Hz, 1H), 0.68 (d, J = 6.8 Hz, 3H). ppm; **$^{13}C\{^{1}H\}$ NMR (126 MHz, DMSO-d_6):** δ 180.8, 145.8, 145.4, 139.8, 139.5, 137.3, 136.4, 134.7, 134.6, 132.6, 128.8, 128.7, 128.4, 128.3, 128.1, 127.8, 127.7, 127.6, 127.6, 126.8, 126.6, 126.5, 126.3, 125.6, 124.6, 124.6, 124.3, 124.2, 123.6, 123.0, 122.7, 39.9, 37.2, 19.2. ppm; **IR (ATR):** ν = 3327 (w), 3158 (w), 2955 (w), 2922 (w), 1587 (w), 1526 (m), 1487 (m), 1454 (w), 1435 (w), 1424 (w), 1355 (w), 1307 (w), 1257 (w), 1241 (m), 1229 (m), 1205 (w), 1170 (w), 1070 (w), 1024 (w), 946 (w), 905 (w), 879 (w), 801 (w), 765 (m), 755 (m), 741 (m), 713 (w), 692 (m), 649 (w), 634 (w), 605 (w); **HR-ESI-MS: *m/z* (%):** 527.1608 ($[M + H]^+$, calcd for $C_{34}H_{27}N_2S_2^+$: 527.1610).

^{1}H and ^{13}C NMR spectra of new compounds

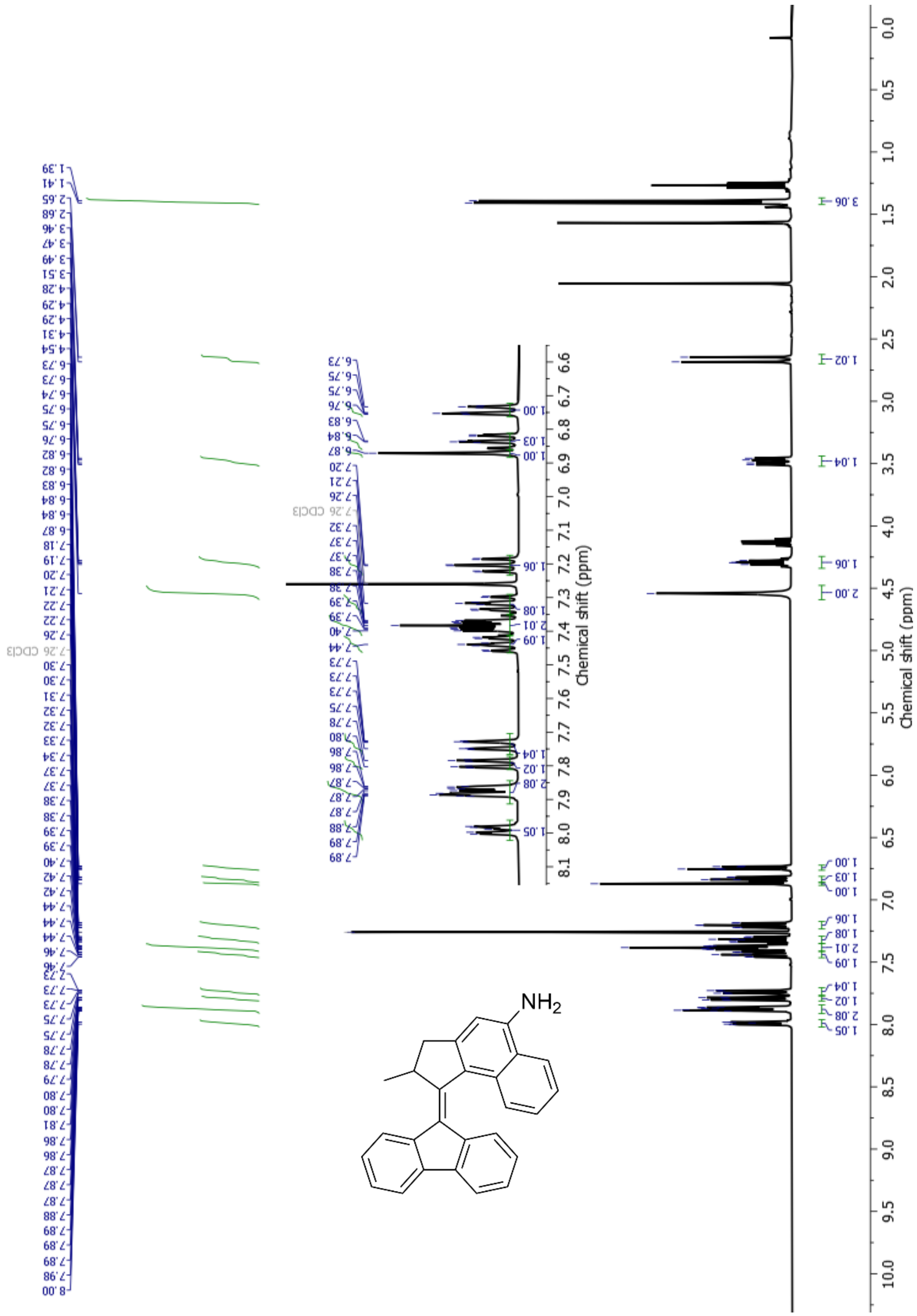

Figure S1. 400 MHz ^{1}H NMR spectrum of compound **4** measured at 298 K in $CDCl_3$.

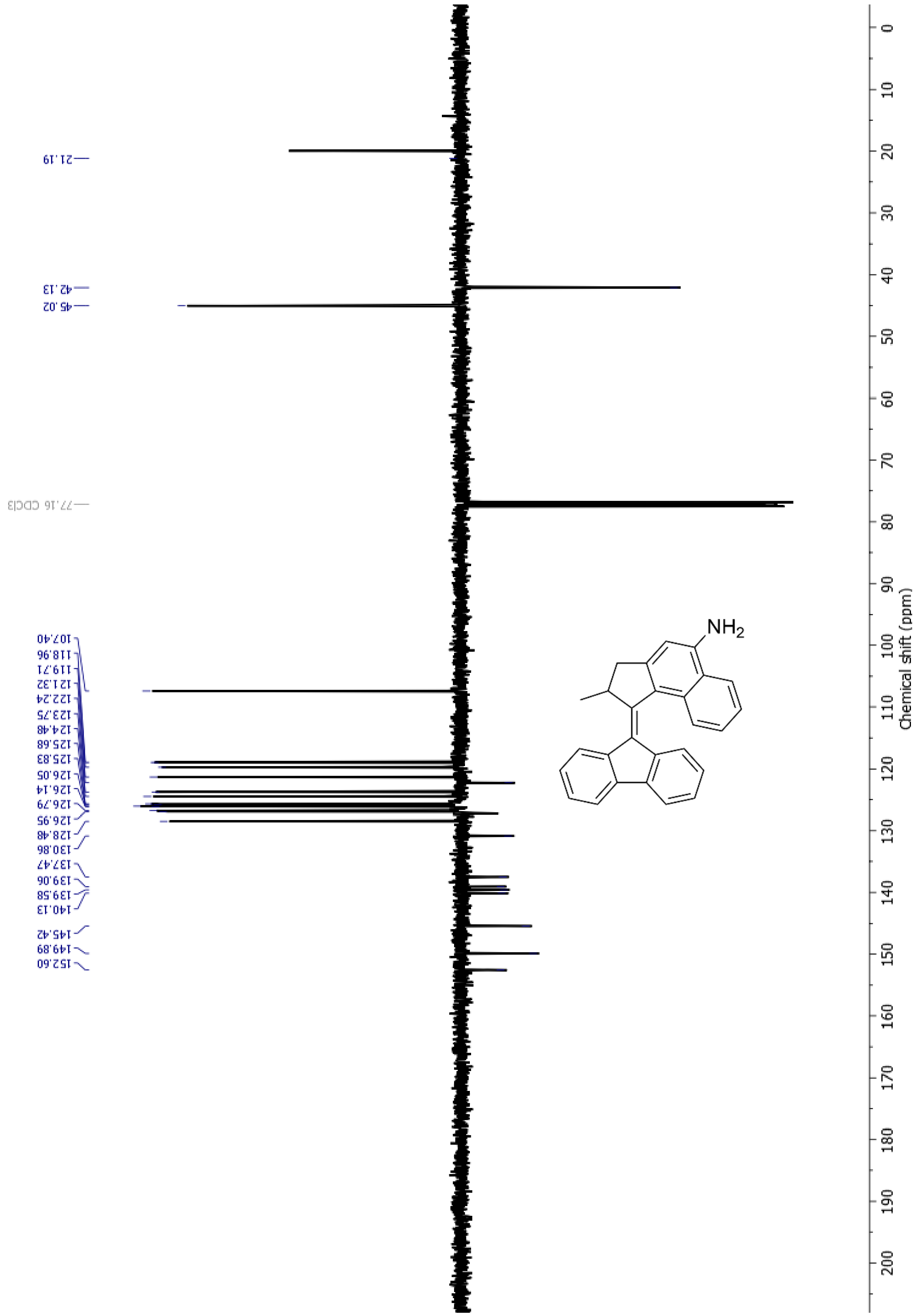

Figure S2. 101 MHz ^{13}C {^{1} H} NMR (APT) spectrum of **4** measured at 298 K in $CDCl_3$.

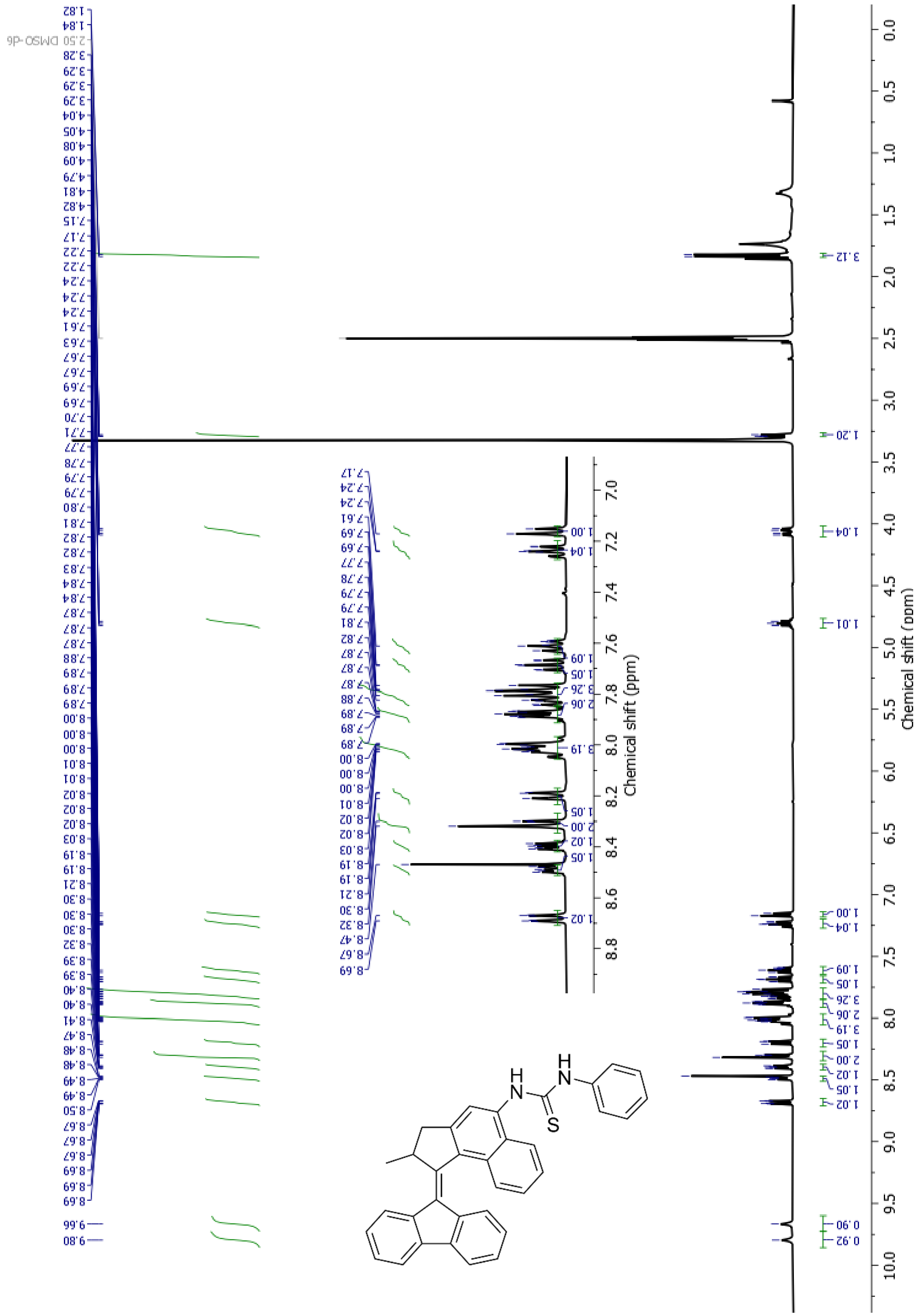

Figure S3. 400 MHz ^{1}H NMR spectrum of compound **1** measured at 298 K in DMSO-d_6.

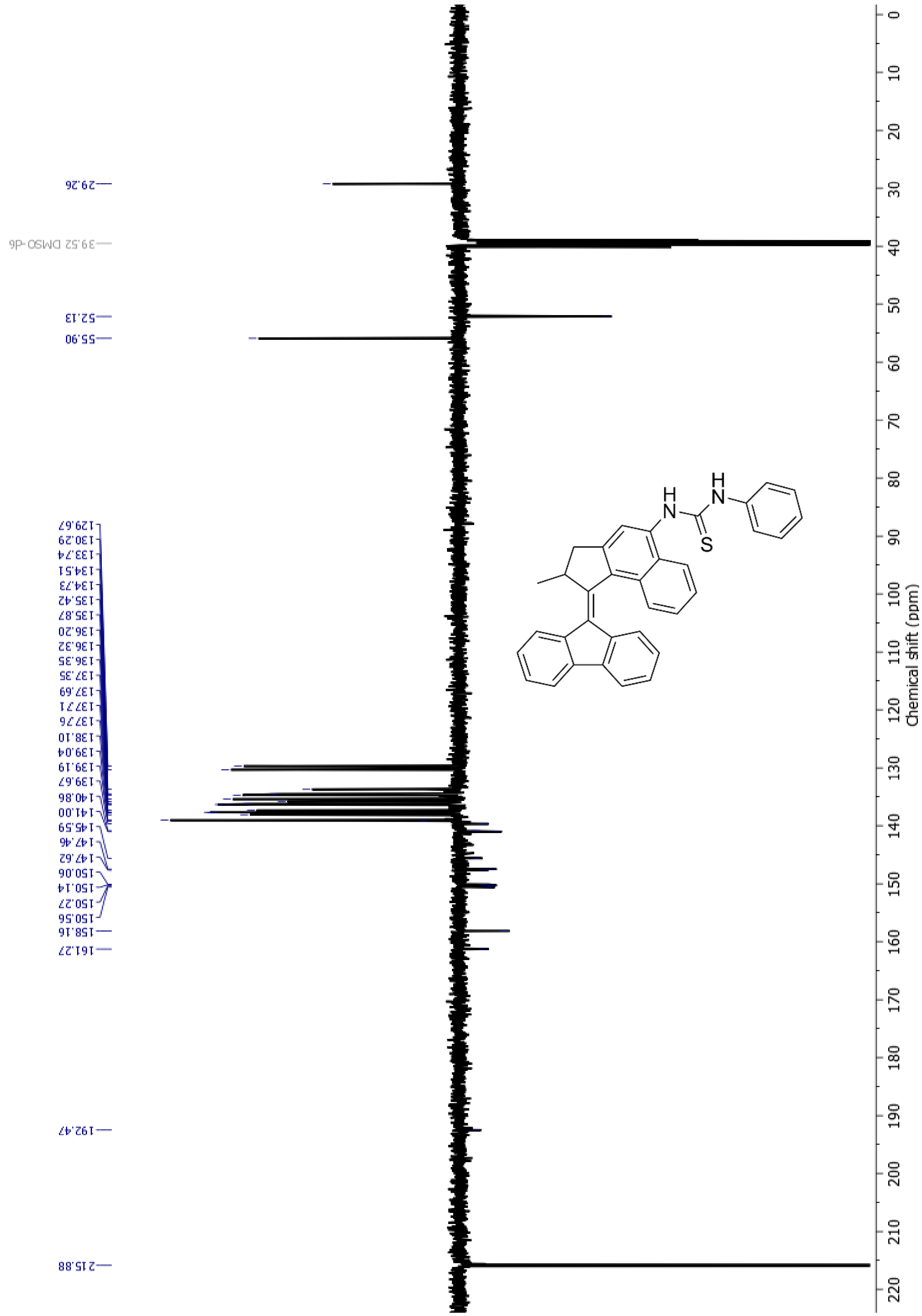

Figure S4. 101 MHz ^{13}C {^{1} H} NMR (APT) spectrum of **1** measured at 298 K in DMSO-d_6.

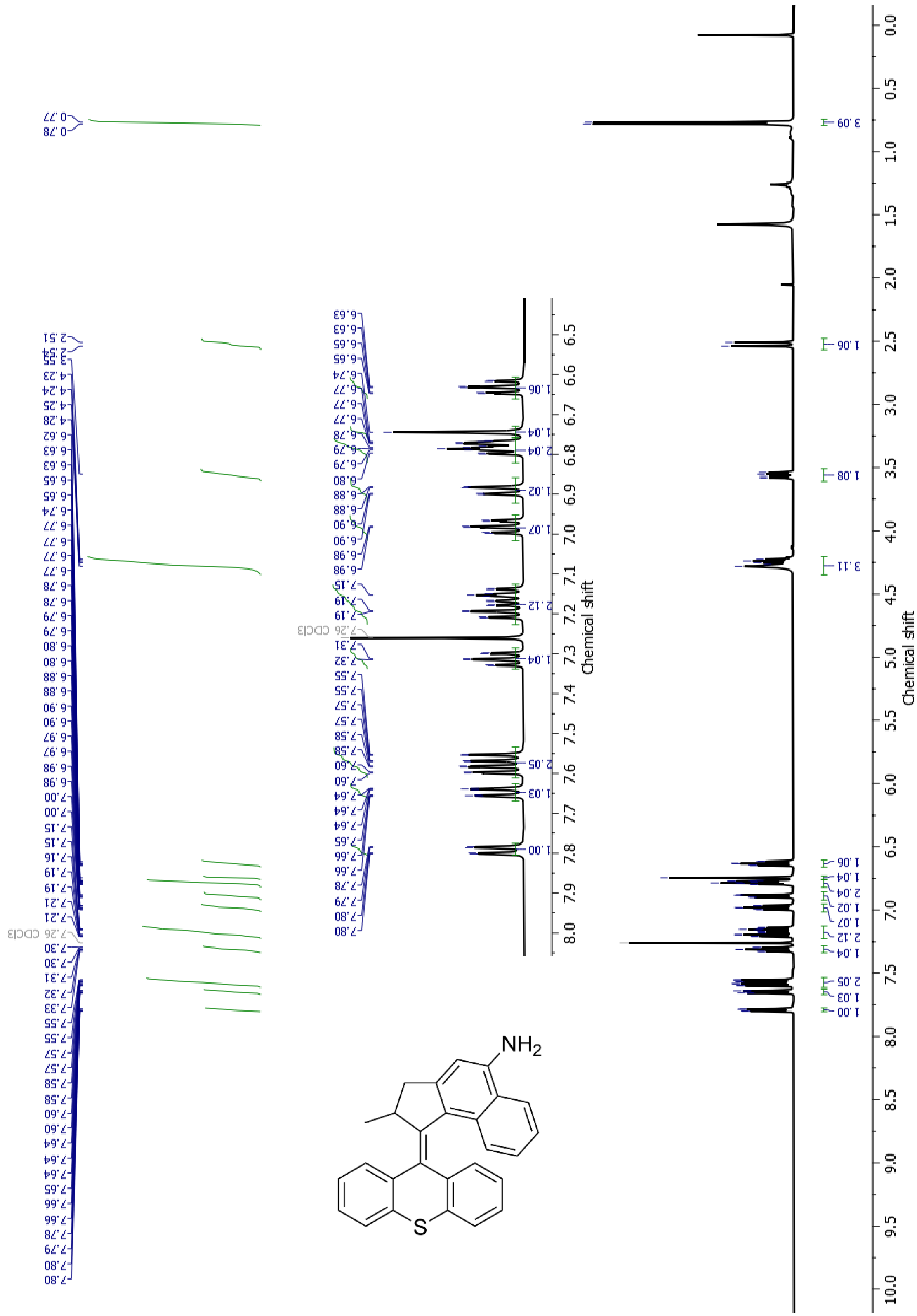

Figure S5. 500 MHz ^{1}H NMR spectrum of compound **6** measured at 298 K in $CDCl_3$.

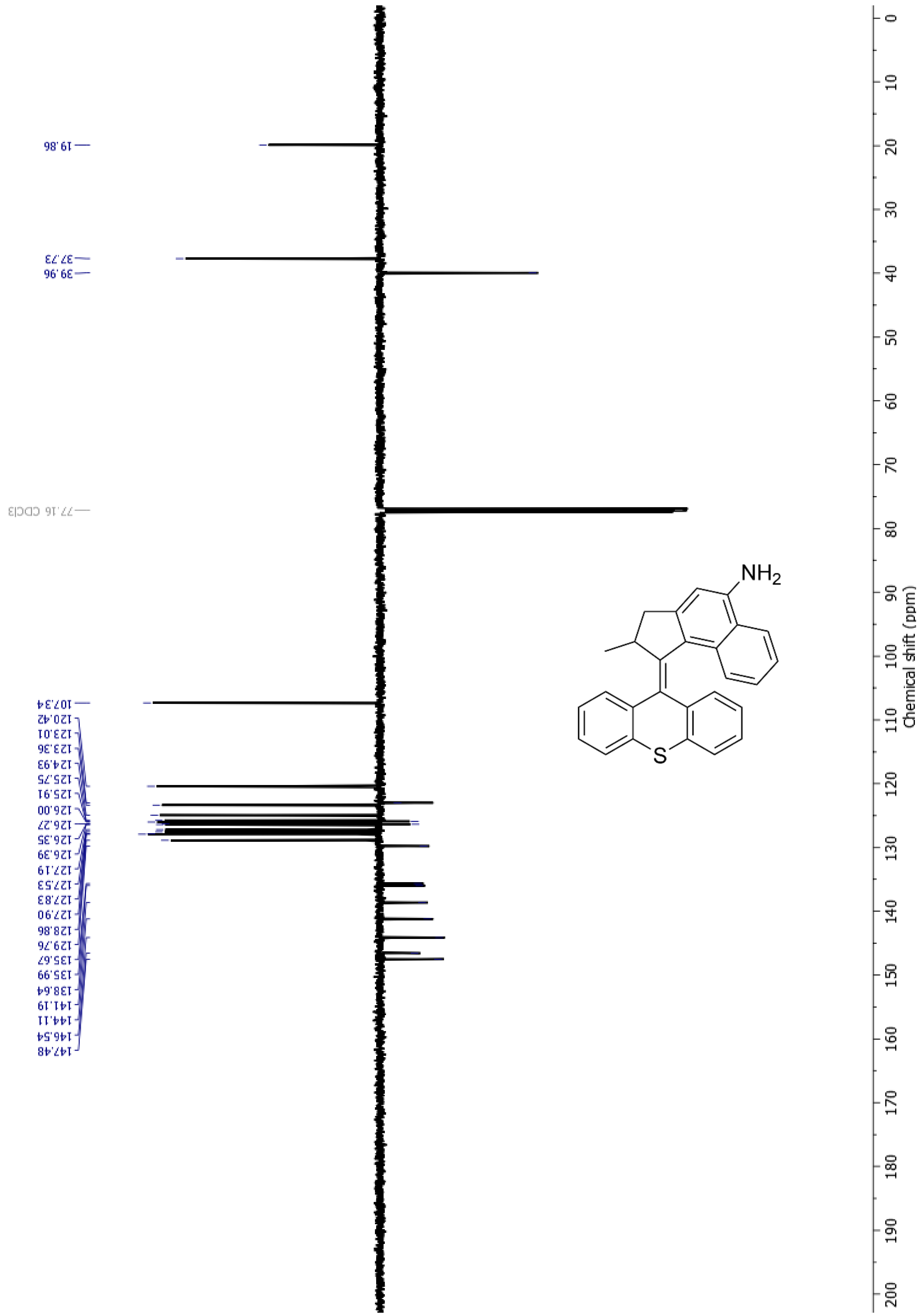

Figure S6. 126 MHz ^{13}C {^{1} H} NMR (APT) spectrum of **6** measured at 298 K in $CDCl_3$.

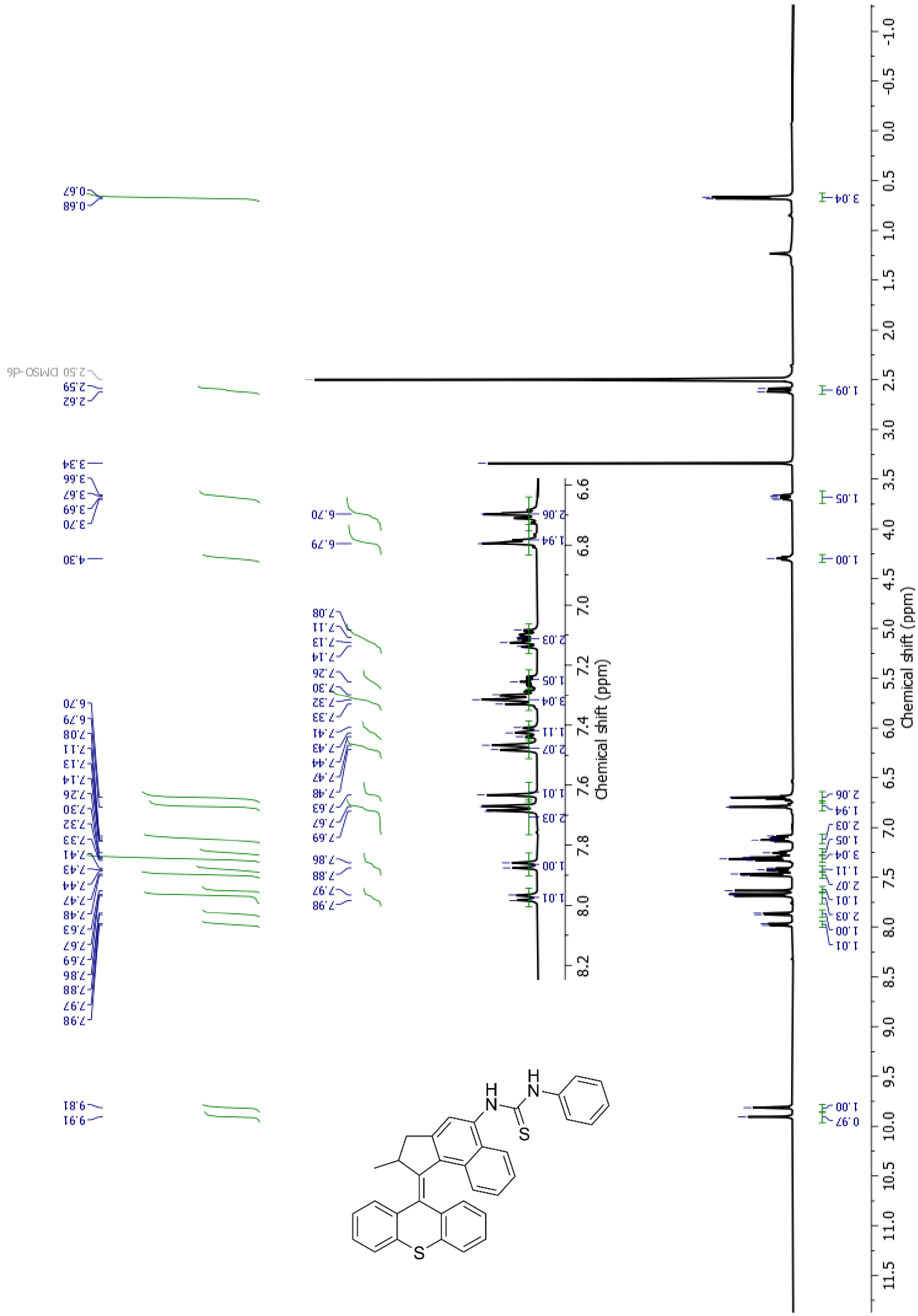

Figure S7. 500 MHz ^{1}H NMR spectrum of compound **2** measured at 298 K in DMSO-d_6.

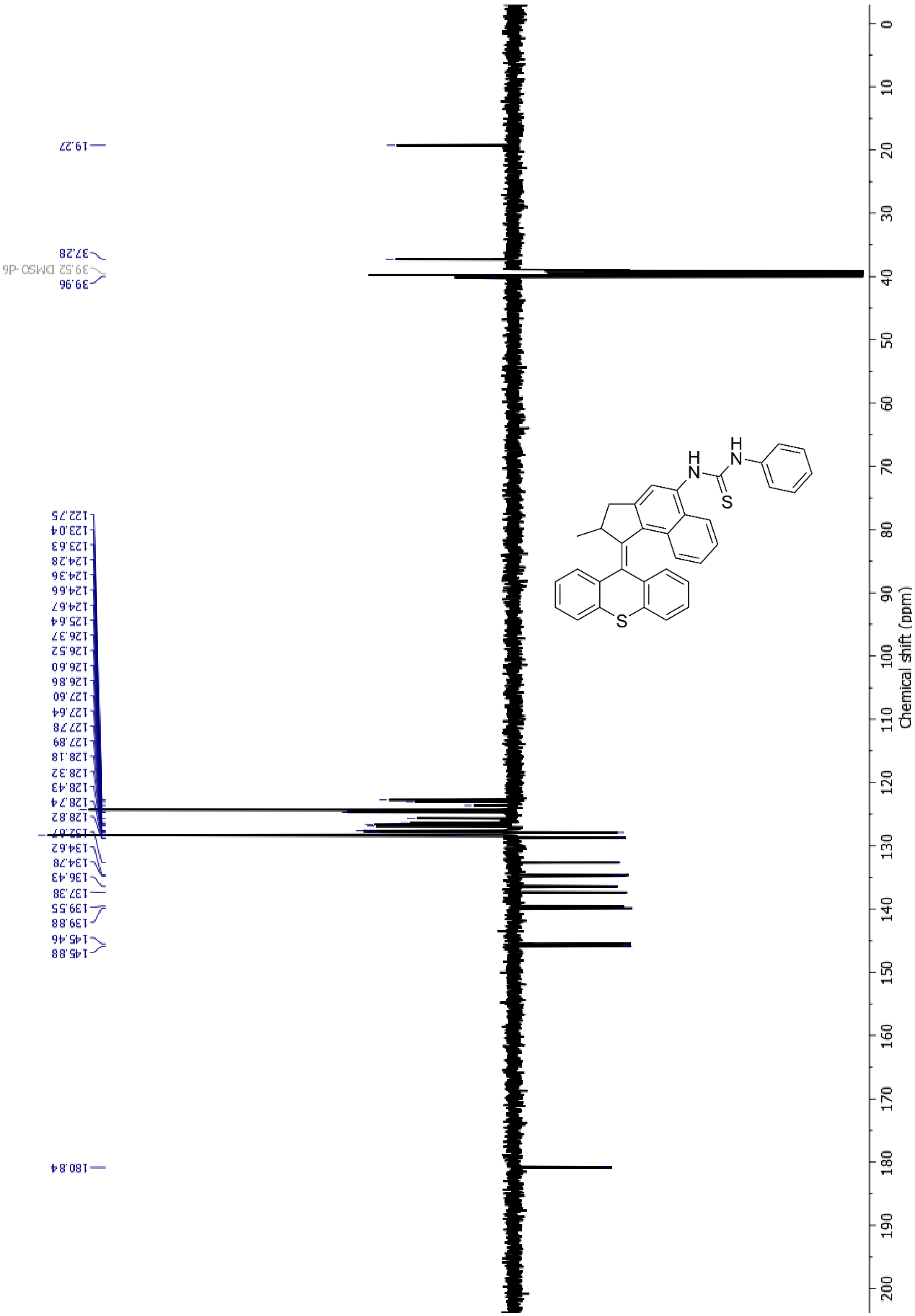

Figure S8. 126 MHz ^{13}C {^{1} H} NMR (APT) spectrum of **2** measured at 298 K in DMSO-d_6.

UV-Vis and ^{1}H NMR photoisomerization studies

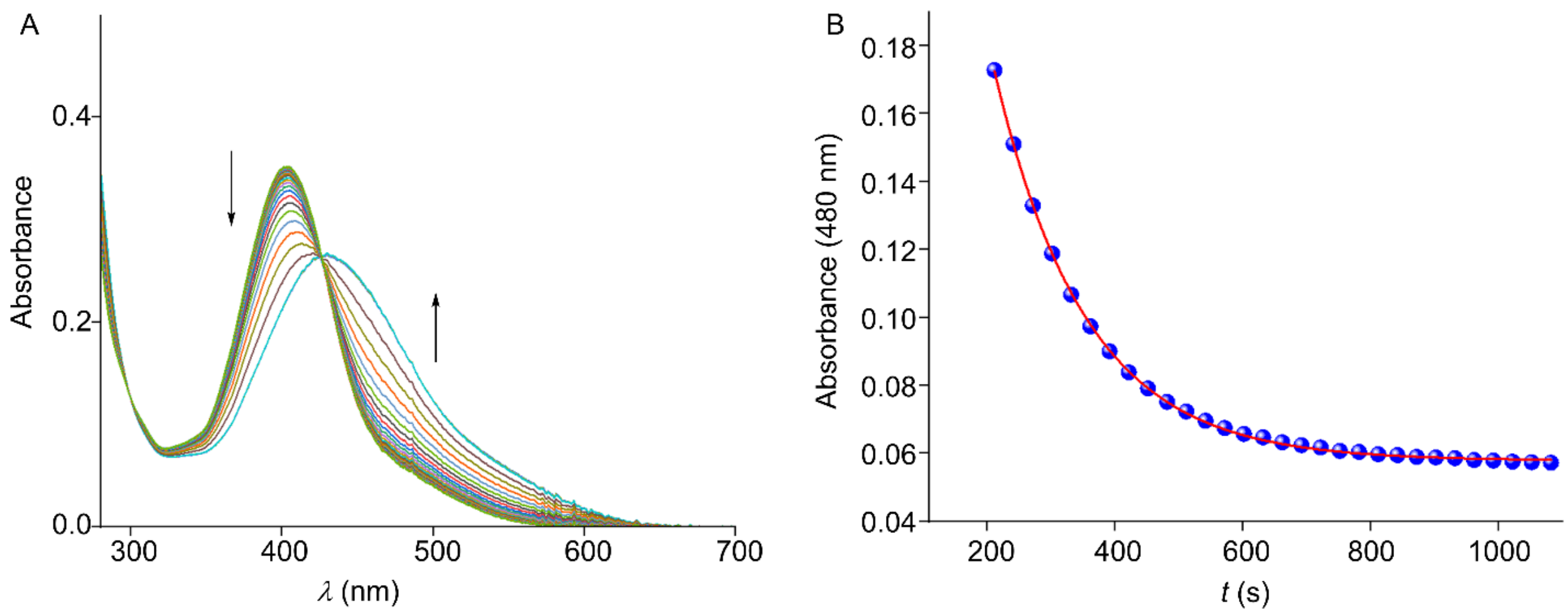

Figure S9. (A) UV-Vis spectra of compound **1** in degassed acetonitrile (2.25×10^{-5} M, 1 cm quartz cuvette) before and after irradiation with 405 nm light at 20 °C (spectra recorded at 30 s intervals). (B) First-order kinetic analysis of the decay in absorbance at 480 nm upon thermal equilibration of the irradiated sample, fitted in Origin software using equation $A(t) = A_1 \exp(-t/t_1) + A_0$, where t_1 is the time constant. The corresponding half-life was calculated as $t_{1/2} = t_1 \ln(2) = 98.8 \pm 0.6$ s at 20 °C.

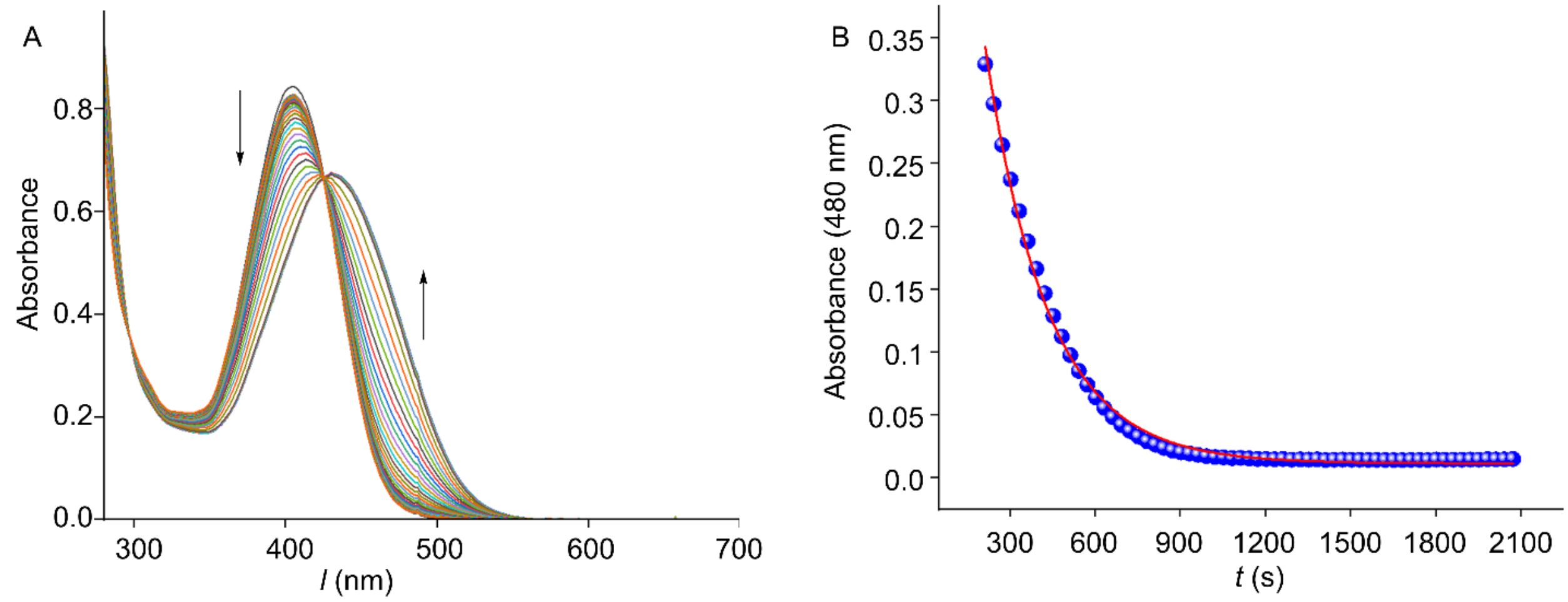

Figure S10. (A) UV-Vis spectra of compound **1** in degassed chloroform (2.50×10^{-5} M, 1 cm quartz cuvette) before and after irradiation with 405 nm light at 20 °C (spectra recorded at 30 s intervals). (B) First-order kinetic analysis of the decay in absorbance at 480 nm upon thermal equilibration of the irradiated sample, fitted in Origin software using equation $A(t) = A_1 \exp(-t/t_1) + A_0$, where t_1 is the time constant. The corresponding half-life was calculated as $t_{1/2} = t_1 \ln(2) = 152.4 \pm 2.5$ s at 20 °C.

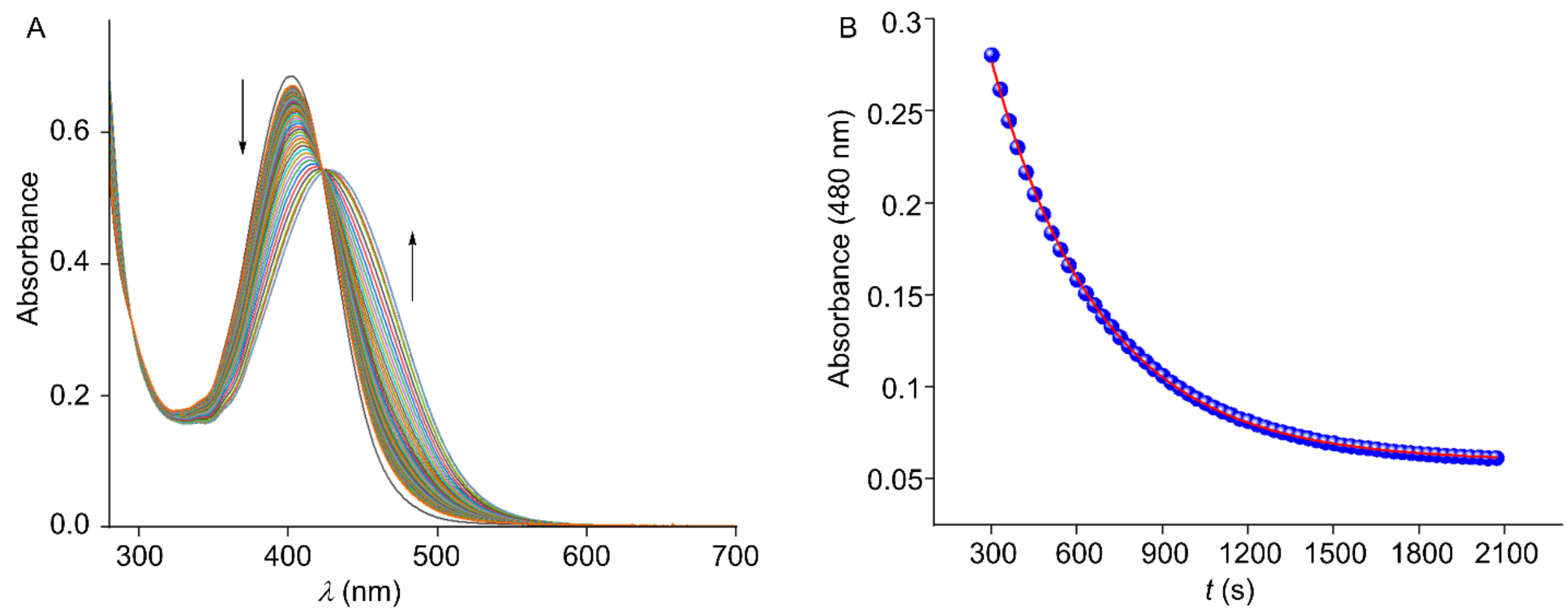

Figure S11. (A) UV-Vis spectra of compound **1** in degassed methanol (2.50×10^{-5} M, 1 cm quartz cuvette) before and after irradiation with 405 nm light at 20 °C (spectra recorded at 30 s intervals). (B) First-order kinetic analysis of the decay in absorbance at 480 nm upon thermal equilibration of the irradiated sample, fitted in Origin software using equation $A(t) = A_1 \exp(-t/t_1) + A_0$, where t_1 is the time constant. The corresponding half-life was calculated as $t_{1/2} = t_1 \ln(2) = 266.1 \pm 1.3$ s at 20 °C.

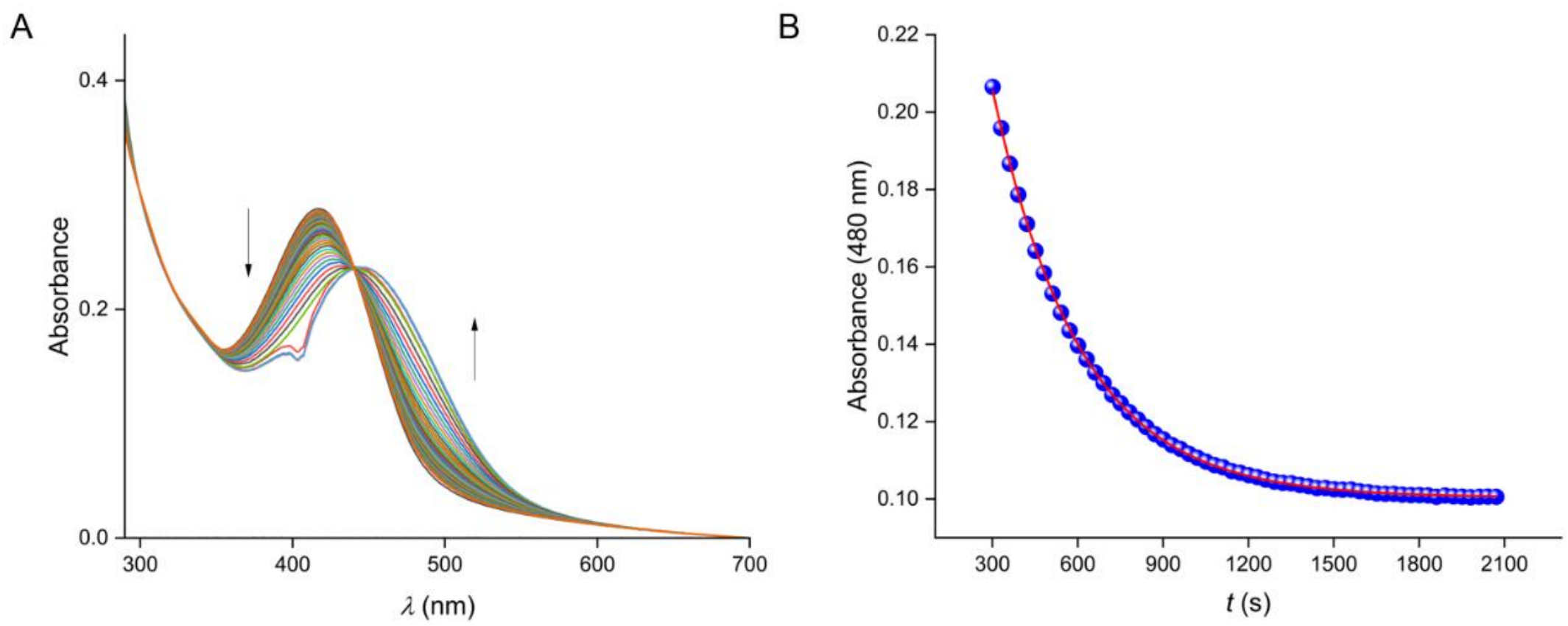

Figure S12. (A) UV-Vis spectra of compound **1** (3 mol%, 1 cm quartz cuvette) in POPC liposome (1.2×10^{-5} M) before and after irradiation with 405 nm light at 20 °C (spectra recorded at 30 s intervals). (B) First-order kinetic analysis of the decay in absorbance at 480 nm upon thermal equilibration of the irradiated sample, fitted in Origin software using equation $A(t) = A_1 \exp(-t/t_1) + A_0$, where t_1 is the time constant. The corresponding half-life was calculated as $t_{1/2} = t_1 \ln(2) = 212.2 \pm 0.6$ s at 20 °C.

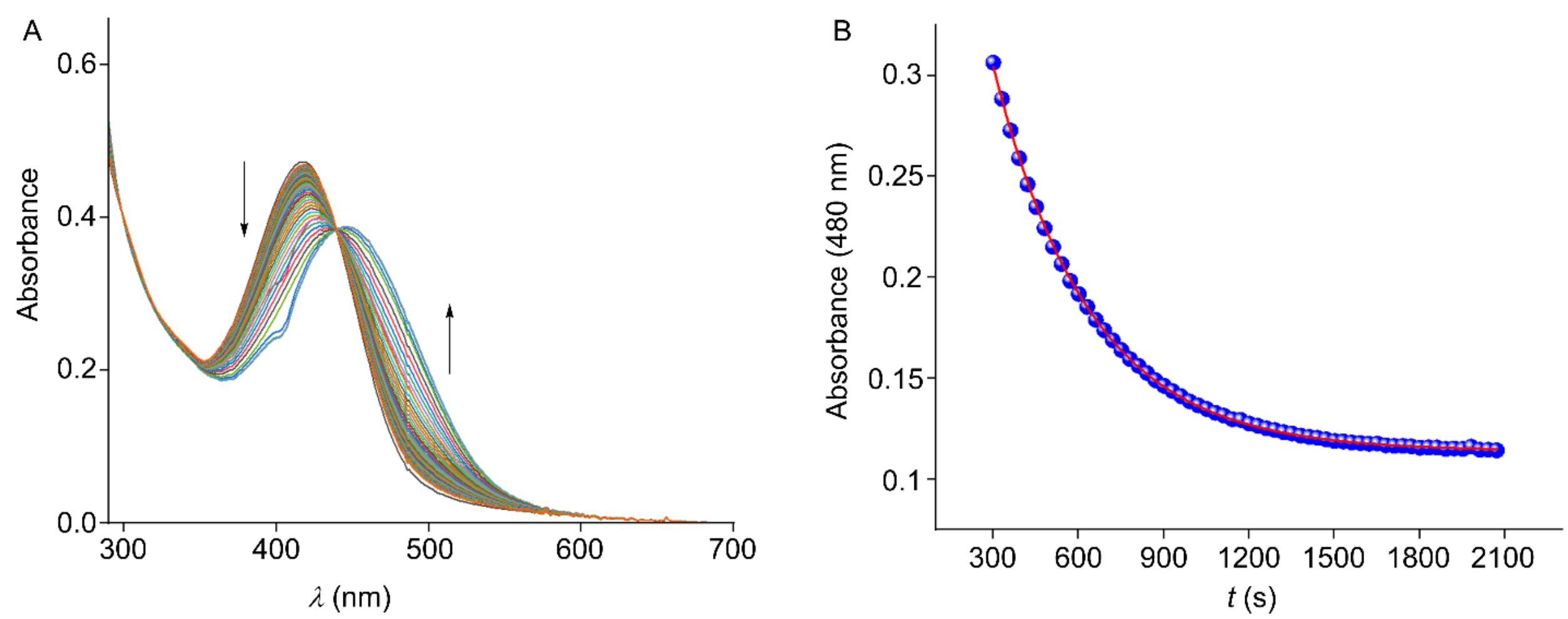

Figure S13. (A) UV-Vis spectra of compound **1** (5 mol%, 1 cm quartz cuvette) in POPC liposome (2.0×10^{-5} M) before and after irradiation with 405 nm light at 20 °C (spectra recorded at 30 s intervals). (B) First-order kinetic analysis of the decay in absorbance at 480 nm upon thermal equilibration of the irradiated sample, fitted in Origin software using equation $A(t) = A_1 \exp(-t/t_1) + A_0$, where t_1 is the time constant. The corresponding half-life was calculated as $t_{1/2} = t_1 \ln(2) = 232.9 \pm 0.8$ s at 20 °C.

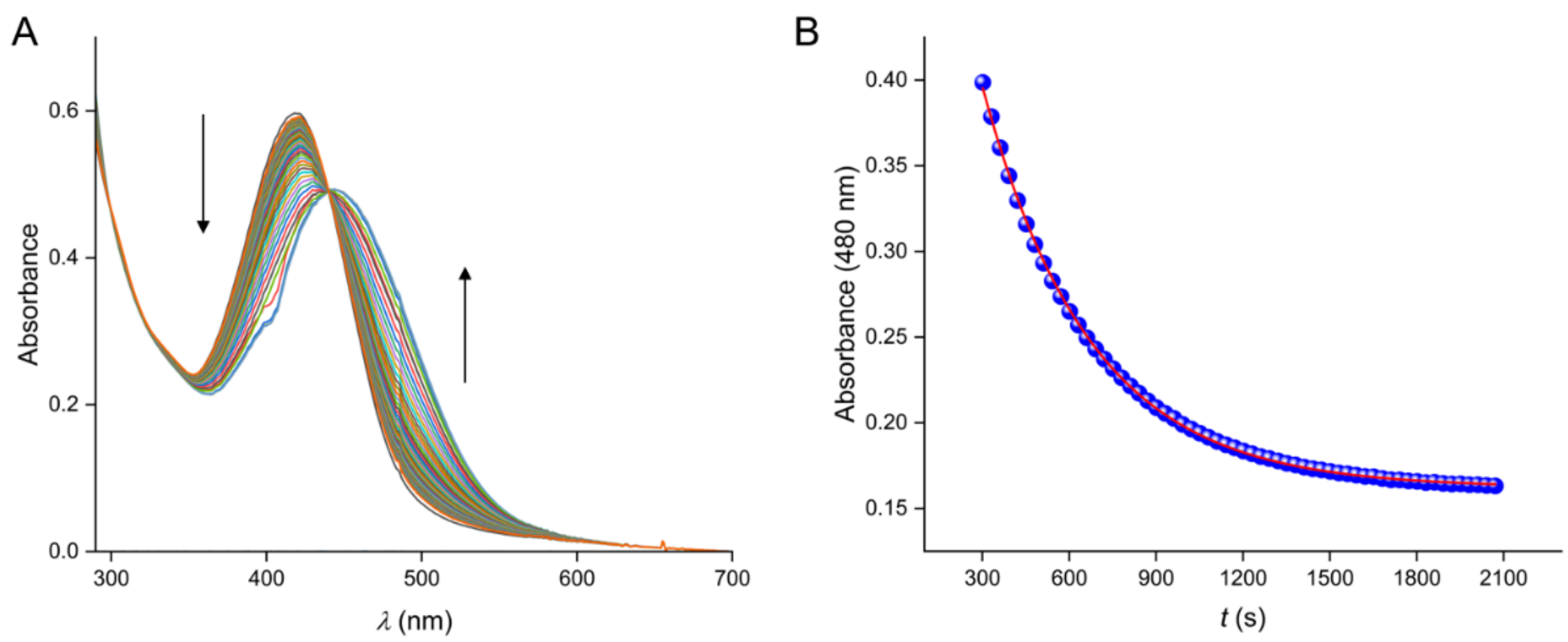

Figure S14. (A) UV-Vis spectra of compound **1** (7 mol%, 1 cm quartz cuvette) in POPC liposome (2.8×10^{-5} M) before and after irradiation with 405 nm light at 20 °C (spectra recorded at 30 s intervals). (B) First-order kinetic analysis of the decay in absorbance at 480 nm upon thermal equilibration of the irradiated sample, fitted in Origin software using equation $A(t) = A_1 \exp(-t/t_1) + A_0$, where t_1 is the time constant. The corresponding half-life was calculated as $t_{1/2} = t_1 \ln(2) = 256.5 \pm 1.2$ s at 20 °C.

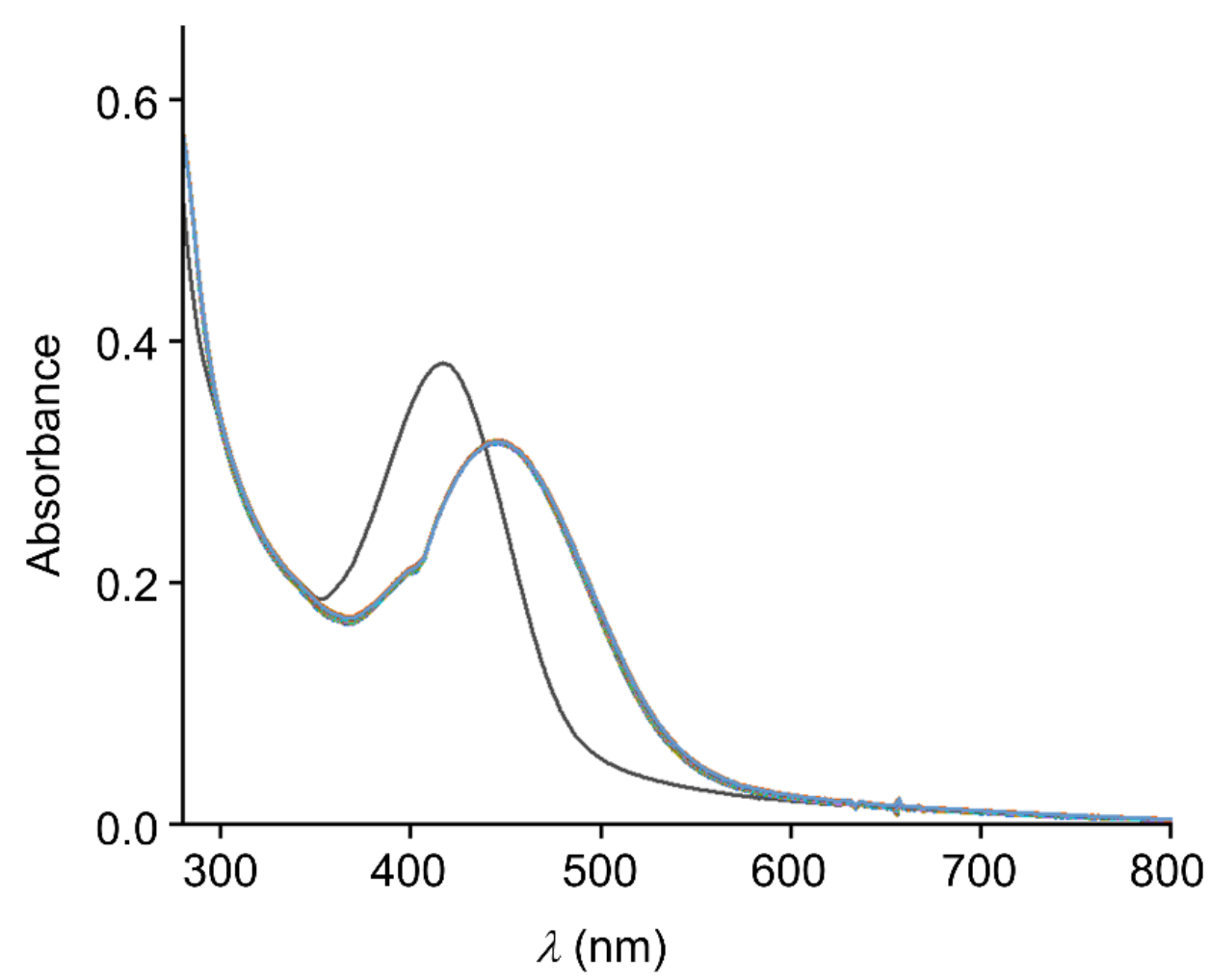

Figure S15. Changes in the UV–vis absorption spectra of compound **1** (5 mol% relative to lipid, 1 cm quartz cuvette) incorporated into POPC liposomes (2.0×10^{-5} M) upon continuous irradiation with 405 nm light at 20 °C for 1 h (spectra recorded at 1 min intervals). The absence of any significant change in the absorbance of the metastable isomer over prolonged irradiation confirms high photostability of compound **1** in the liposomal bilayer, i.e., compound **1** exhibits excellent fatigue resistance.

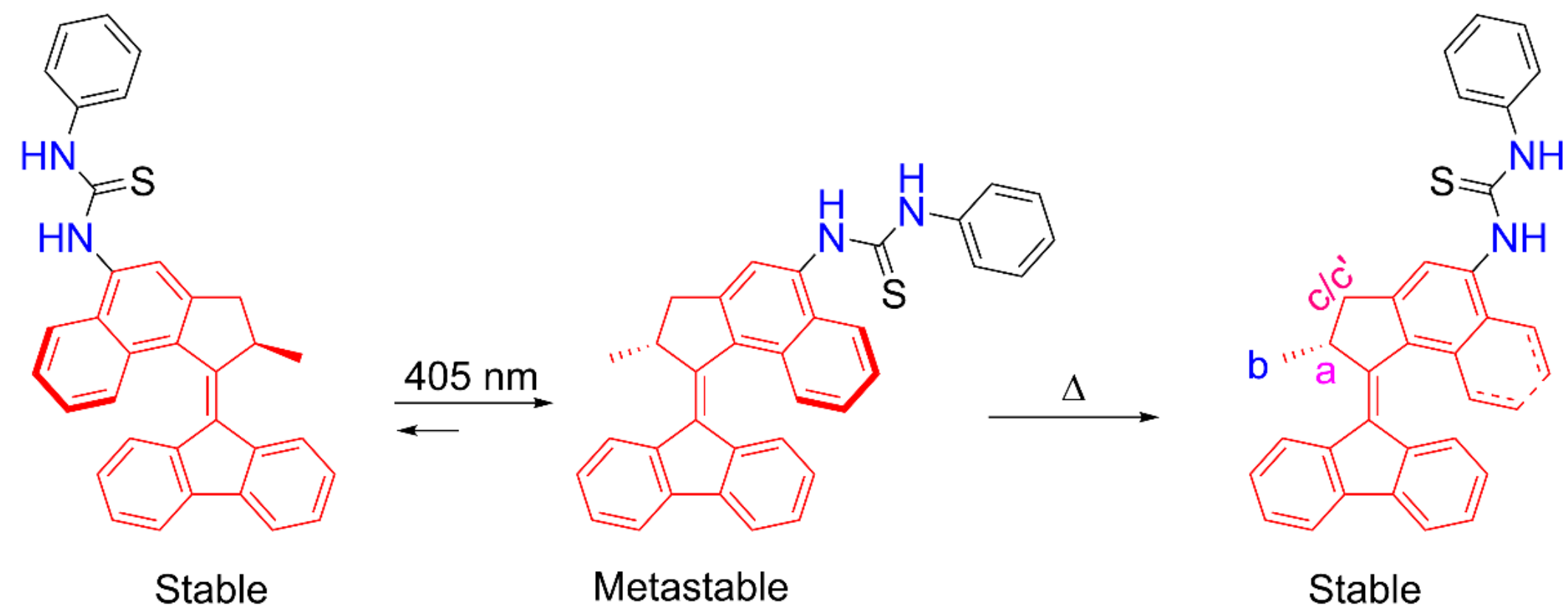

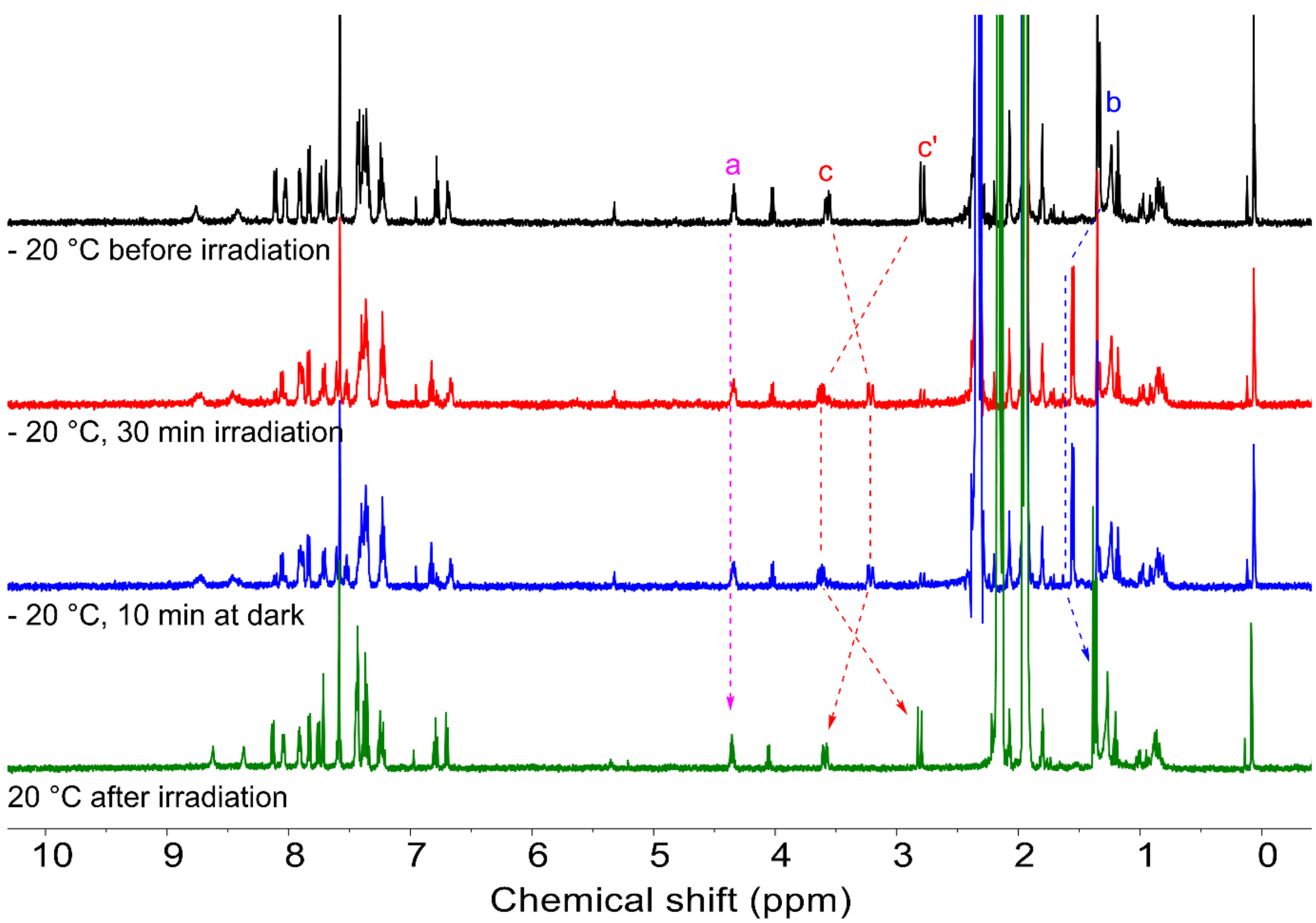

Figure S16. ^{1}H NMR spectra (500 MHz, 1 mM in acetonitrile-*d*$_3$) of compound **1** before and after 405 nm irradiation at –20 °C, followed by standing in the dark at –20 °C and 20 °C to allow thermal helix inversion (THI). Irradiation was performed using a Thorlab model M405F3 LED coupled to a 600 mm optical fiber, which guided the light into the NMR tube inside the spectrometer.[2]

^{1}H NMR titrations and data fitting

Prior to the binding studies, a ^{1}H NMR dilution experiment was conducted to identify the concentration regime in which compound **1** does not self-aggregate. Spectra were recorded in acetonitrile-d_3 at 293 K over a concentration range of 5.0 – 0.625 mM. A very minor chemical shift change ($\Delta\delta$ = 0.007 ppm) was observed for the NH protons upon dilution from 5.0 to 2.5 mM; however, no further shifting was detected by diluting further, indicating that compound **1** does not self-aggregate below 2.5 mM (see Figure S15). On this basis, ^{1}H NMR binding titrations were performed by dissolving compound **1** in acetonitrile-d_3 to give a 1.0 mM solution, from which an initial spectrum was recorded (500 MHz, 293 K). Chloride was introduced by stepwise addition of a tetrabutylammonium chloride ($[Bu_4N]^+[Cl]^-$) solution prepared in acetonitrile-d_3. After each addition (0.00–31.25 equivalents), the sample was mixed thoroughly, and a spectrum was acquired. Analysis of the titration data was performed using dilution-corrected concentrations.

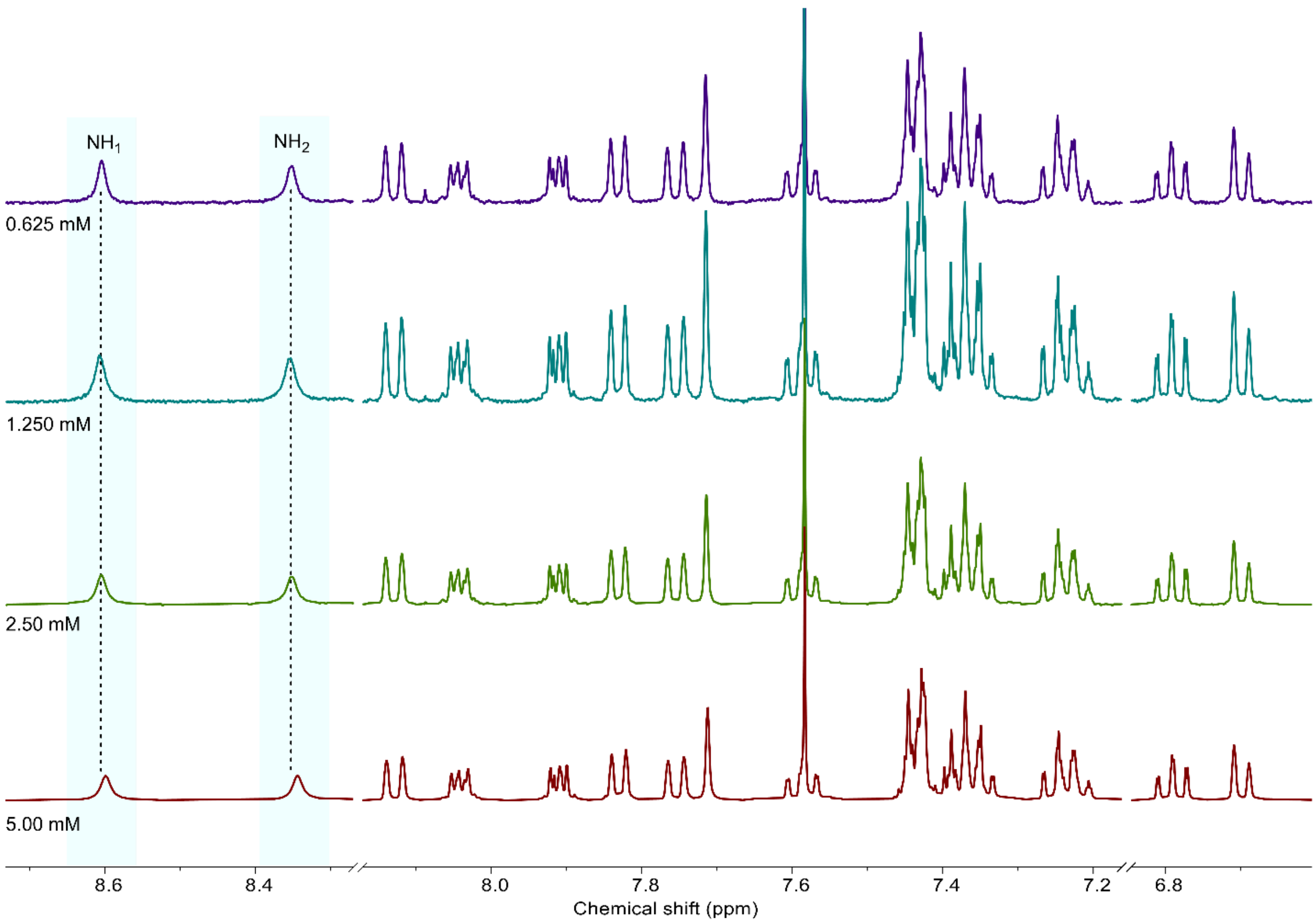

Figure S17. ^{1}H NMR spectral changes (500 MHz, 293 K) of compound **1** in acetonitrile-*d₃* upon stepwise dilution (from bottom to top: 5.00 mM, 2.50 mM, 1.250 mM, and 0.625 mM).

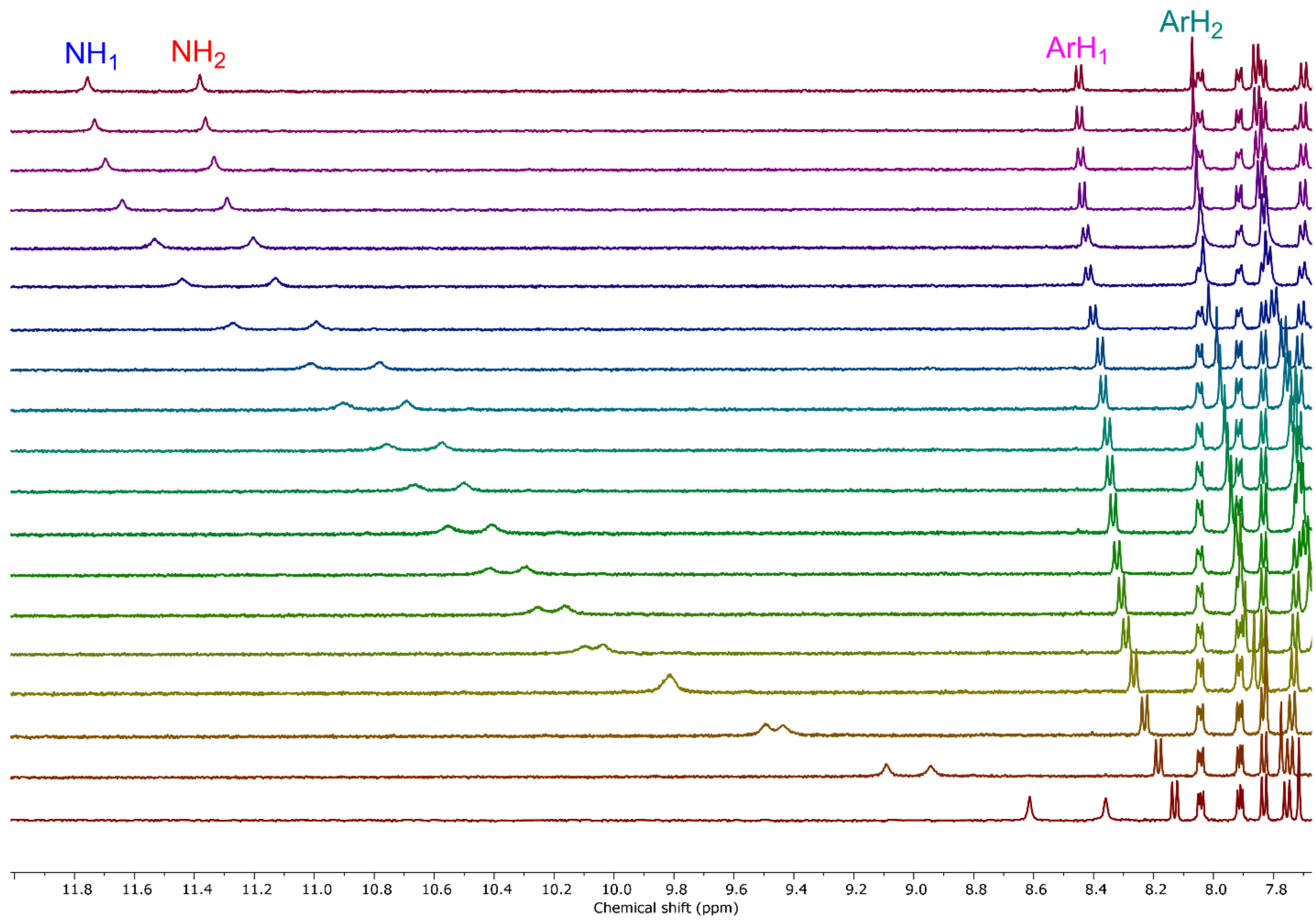

Figure S18. ^{1}H NMR spectral changes (500 MHz, 293 K) of compound **1** in acetonitrile-d_3 (1 mM) upon the stepwise addition of $[Bu_4N]^+[Cl]^-$ (from bottom to top: 0.00, 0.20, 0.41, 0.62, 0.83, 1.04, 1.30, 1.56, 1.82, 2.08, 2.60, 3.12, 5.20, 7.81, 10.41, 15.62, 20.83, 26.04, 31.25 equivalents).

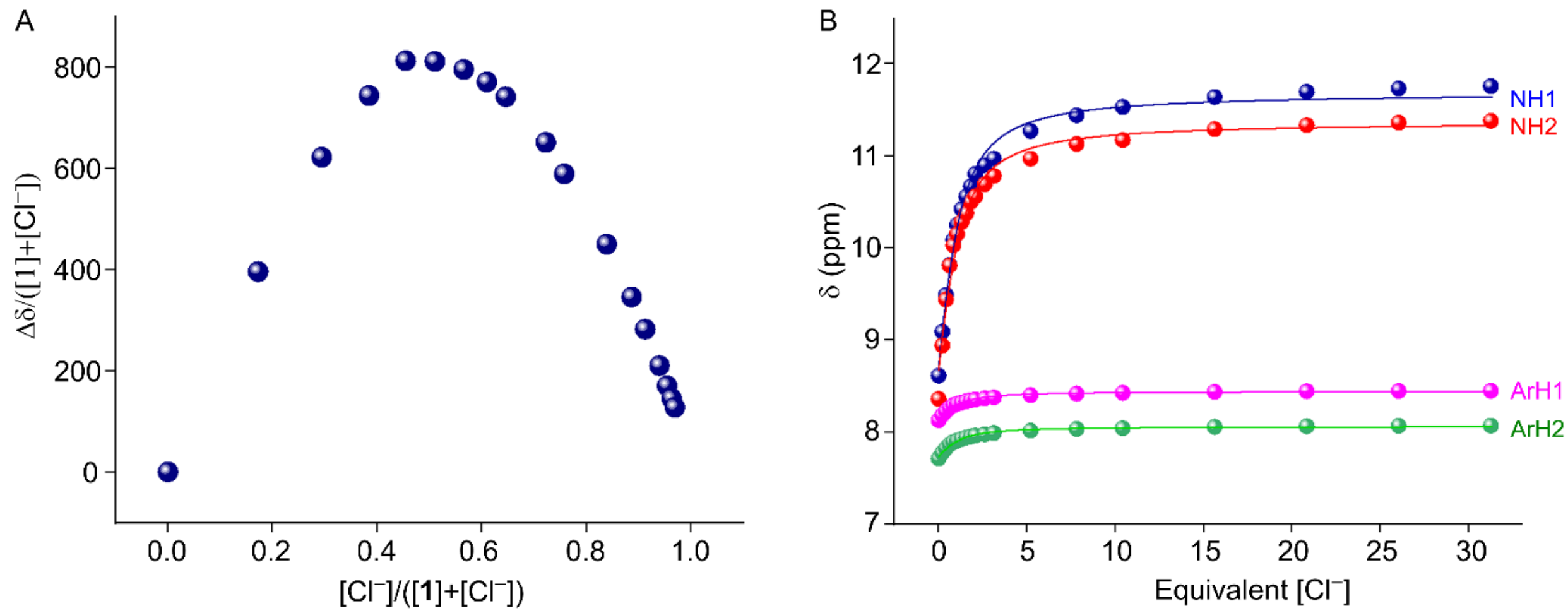

Figure S19. (A) Modified Job plot analysis indicating a 1:1 binding stoichiometry. (B) Titration curve for the addition of $[Bu_4N]^+[Cl]^-$ to receptor **1** and data fit obtained by using the chemical shifts of multiple ^{1}H NMR signals and a 1:1 binding model. $K_{11} = 1.9 \times 10^3$ M^{-1}. Data fits were performed with HypNMR.[3]

Transmembrane transport experiments

Chloride Selective Electrode Assay: A thin lipid film was acquired by drying a chloroform solution of a mixture of POPC and compound **1** (0.2 to 15 mol% with respect to POPC) in a round-bottom flask. This film was dried under vacuum for 16 h. The dried lipid film was hydrated by vortexing with KCl solution (300 mM buffered to pH 7.2 using HEPES 10 mM). The lipid suspension was then subjected to nine freeze/thaw cycles and was extruded 21 times through a 200 nm polycarbonate membrane (NucleoporeTM) using an extruder set (Avanti Polar Lipids, Inc) to obtain the vesicle stock solution. The vesicles were then passed through a Sephadex® G-25 column saturated with HEPES buffered external potassium gluconate (KGlu) at pH 7.2, which allowed the exchange of any unencapsulated KCl for KGlu.

Chloride concentrations during the cationophore-coupled assay were determined using an Accumet chloride-selective electrode. The electrode was calibrated against sodium chloride solutions of known concentrations prior to each experiment according to the supplier's manual. For each measurement, the lipid stock solution was diluted with the external buffered solution to a standard volume (5.0 mL) with a lipid concentration of 1.0 mM. A cationophore (valinomycin or monensin) was added as a DMSO solution (1 µM, 0.1 mol%) to start the experiment and the chloride efflux was monitored using the chloride selective electrode. After 10 minutes detergent (50 µL of Triton X-100 (11 w%) in H_2O: DMSO (7:1 v/v)) was added to lyse the vesicles and the 100% chloride efflux reading was taken at 12 minutes. The electrode readings were converted to chloride concentrations using a standard calibration and subsequently converted to percentages. The initial rates of Cl^- transport (k_{ini}), observed under each condition, were calculated by fitting the obtained chloride efflux with the asymptotic function $y = a - b \cdot c^x$ using Origin 9, where y is

the chloride efflux (%), x is time (s) and k_{ini} is then given by $k_{ini} = -\, b \cdot \ln(c)$ (obtained in % s^{-1}), or by fitting the initial linear range of the obtained chloride efflux to $y = a + b \cdot x$, where y is the chloride efflux (%), x is time (s) and k_{ini} is given by the slope b.

For the regulation of anion transport, the liposomes containing compound **1** were irradiated *ex situ* with 405 nm light (using a Thorlab model M405LP1 high-power LED, 1200 mW, positioned at a distance of 2 cm from the sample) for 300 s. After the cessation of light, the electrode readings were immediately taken following the aforementioned protocol.

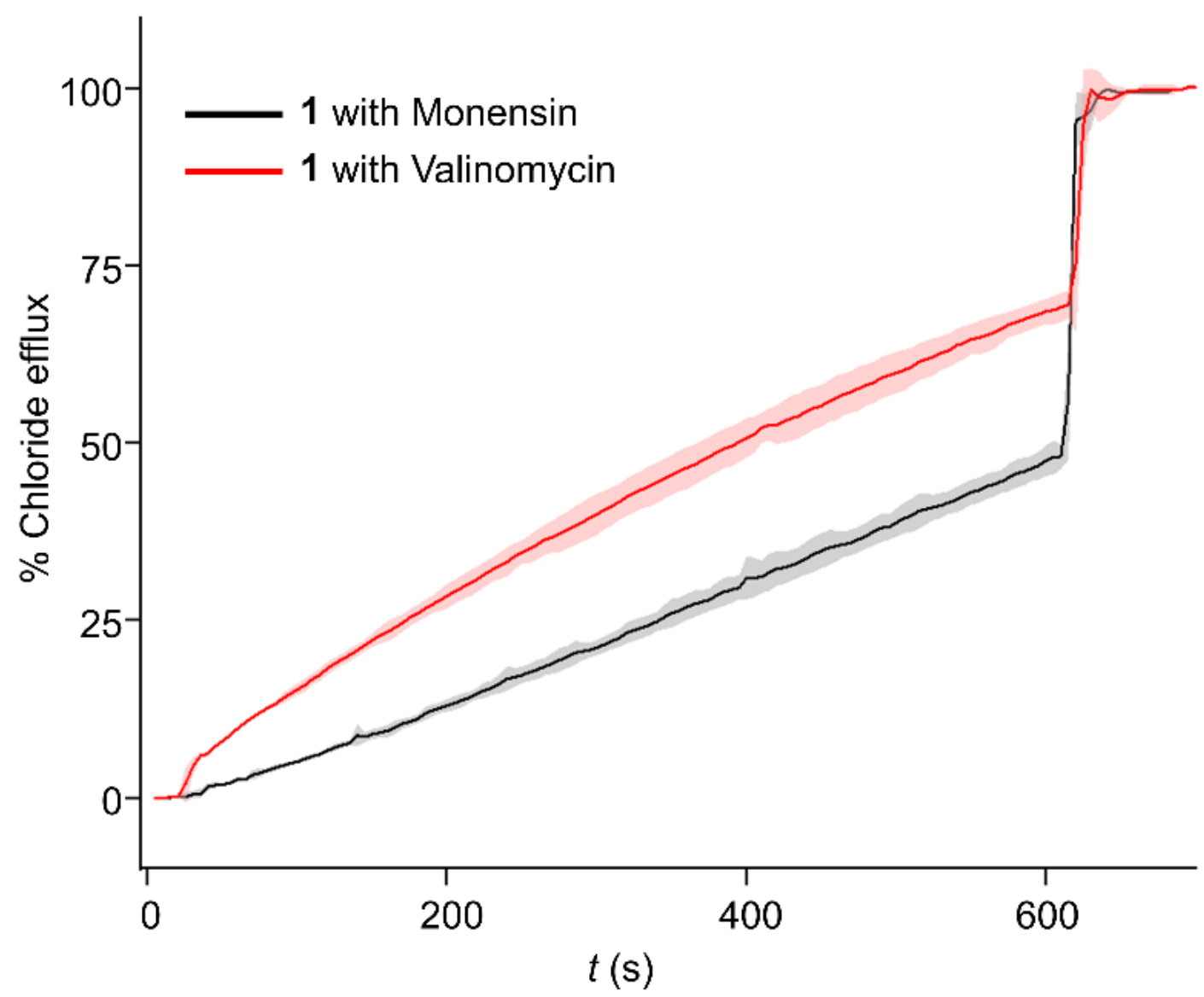

Figure S20. Chloride efflux from liposomes containing 5 mol% of compound **1** in the presence of monensin (0.1 mol%, black trace) or valinomycin (0.1 mol%, red trace). The higher chloride efflux observed in the presence of valinomycin as compared to monensin indicates that compound **1** operates with preference for an electrogenic Cl^- uniport over an electroneutral HCl symport or Cl^-/OH^- antiport transport mechanism.

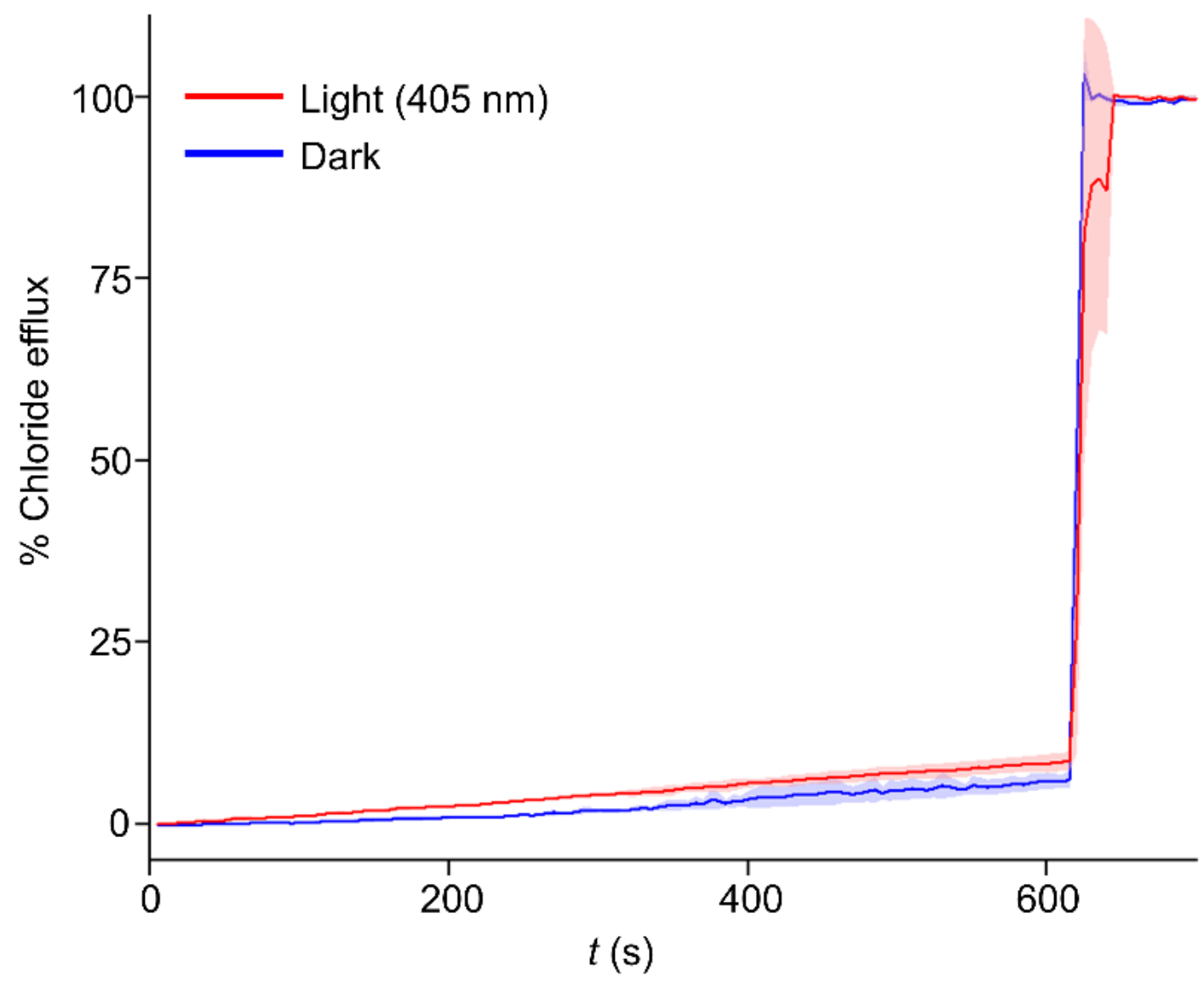

Figure S21. Chloride efflux from liposomes containing 5 mol% of compound **1** without the addition of valinomycin. Two sets of experiments were conducted under identical conditions: (i) in the dark (blue) and (ii) after *ex situ* irradiation at 405 nm for 5 min (red). In both cases, the chloride concentration remained unchanged until Triton X-100 addition, indicating that light irradiation neither compromises membrane integrity nor causes chloride leakage.

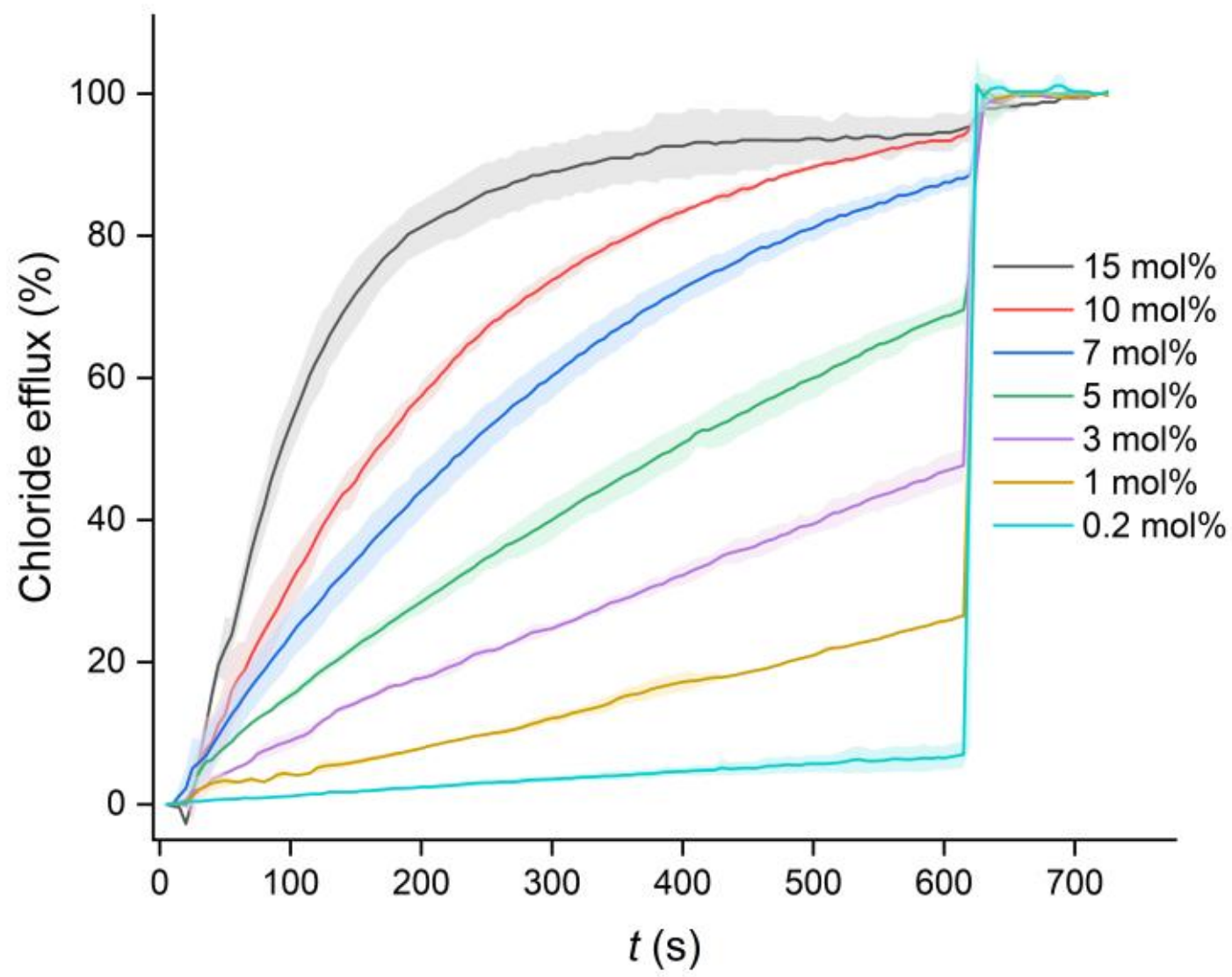

Figure S22. Dose-dependent chloride efflux (0.2 – 15 mol%) against time facilitated by stable-**1** monitored using a chloride selective electrode.

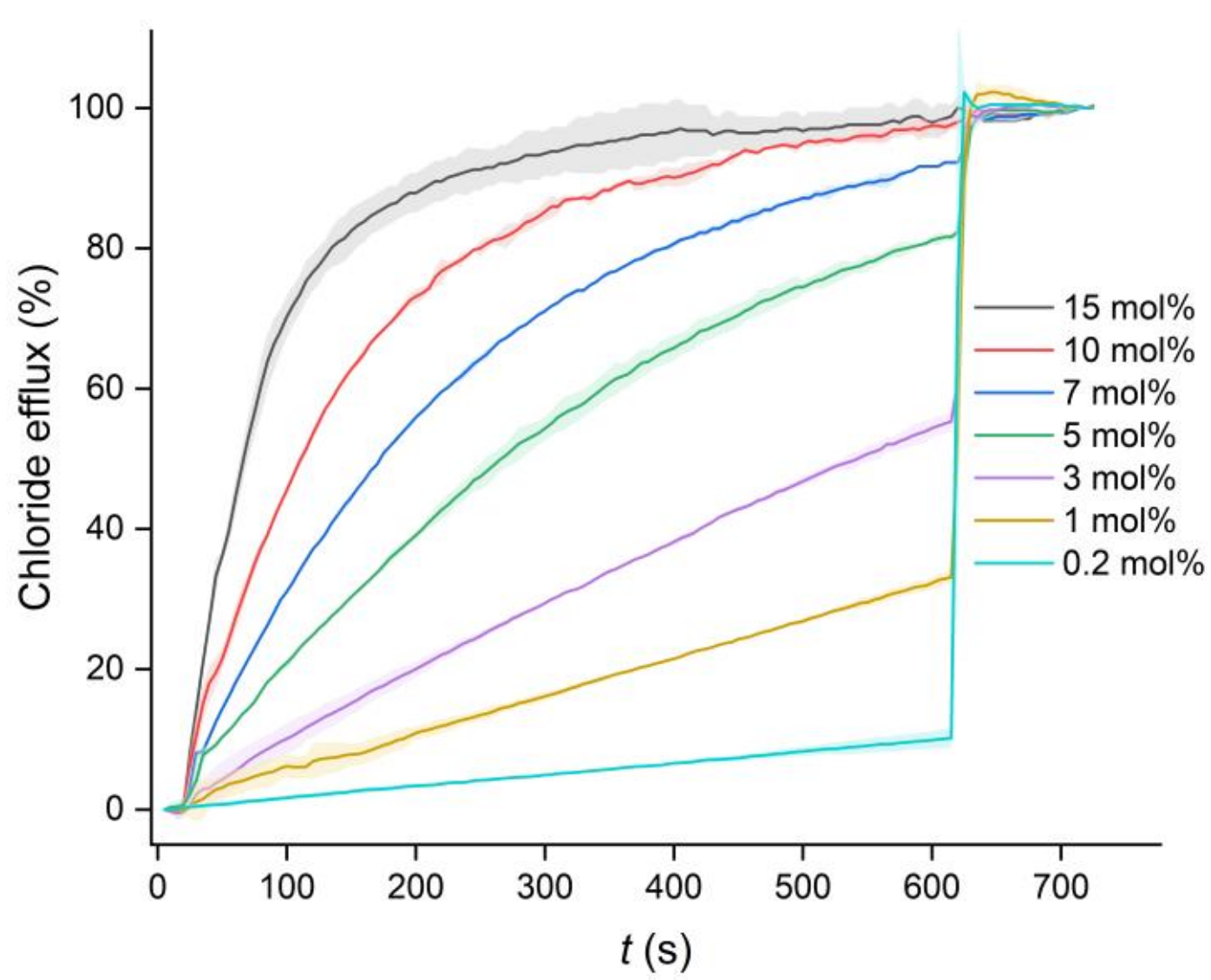

Figure S23. Dose-dependent chloride efflux (0.2 – 15 mol%) against time facilitated by **1** after *ex situ* irradiation at 405 nm for 5 min, monitored using a chloride selective electrode.

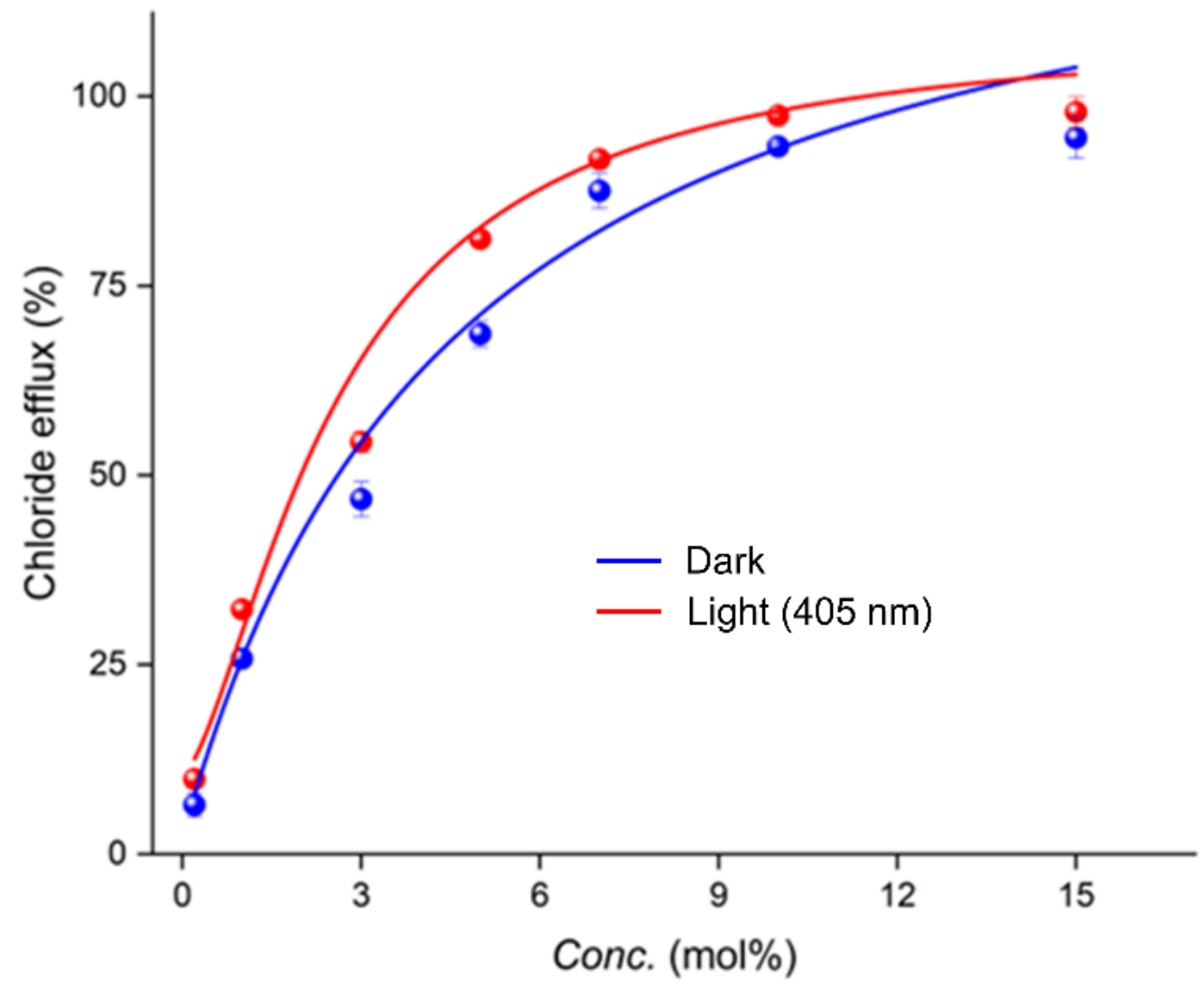

Figure S24. Hill plots of activity versus concentration of the stable isomer in dark (blue) and after *ex situ* irradiation at 405 nm for 5 min (red).

Osmotic Response Assay: A thin lipid film was acquired by drying a chloroform solution of a mixture of POPC and compound **1** (3, 5, and 7 mol% with respect to POPC) or compound **2** (5 mol% with respect to POPC) in a round-bottom flask. This film was dried under vacuum for 16 h. The dried lipid film was hydrated by vortexing with KCl solution (300 mM buffered to pH 7.2 using HEPES 10 mM). The lipid suspension was then subjected to nine freeze/thaw cycles and was extruded 21 times through a 400 nm polycarbonate membrane (NucleoporeTM) using an extruder set (Avanti Polar Lipids, Inc) to obtain the vesicle stock solution. The vesicles were then passed through a Sephadex® G-25 column saturated with HEPES buffered external potassium gluconate (KGlu) at pH 7.2, which allowed the exchange of any unencapsulated KCl for KGlu.

Vesicles containing KCl (300 mM, buffered to pH 7.2 using 10 mM HEPES) were suspended in KGlu (300 mM buffered to pH 7.2 using 10 mM HEPES) to make a test solution of 0.4 mM lipid

in 2.2 mL external solution. The sample was stirred at 293 K, and a DMSO solution of valinomycin (0.4 μM, 0.1 mol%) was added to commence the experiment. The 90° light scattering intensity at 600 nm was monitored. At 35 min, prodigiosin (0.04 μM, 0.01 mol%) and FCCP (4 μM, 1.0 mol%) were added to calibrate 100% Cl^- efflux. The fractional light scattering intensity (I_f) was calculated using the following equation:

$$I_f = \frac{I_t - I_0}{I_c - I_0}$$

Where, I_t is the light scattering intensity at a given time, I_0 is the light scattering intensity at 0 s, and I_c is the light scattering intensity after the addition of prodigiosin and FCCP.

For the regulation of anion transport, the liposomes containing either compound **1** or **2** were irradiated *in situ* with 405 nm light (using a Thorlab model M405LP1 high-power LED, 1200 mW, positioned at a distance of 2 cm from the sample). The system was subjected to alternating light and dark periods of 300 seconds to assess the effect of light modulation on transport performance.

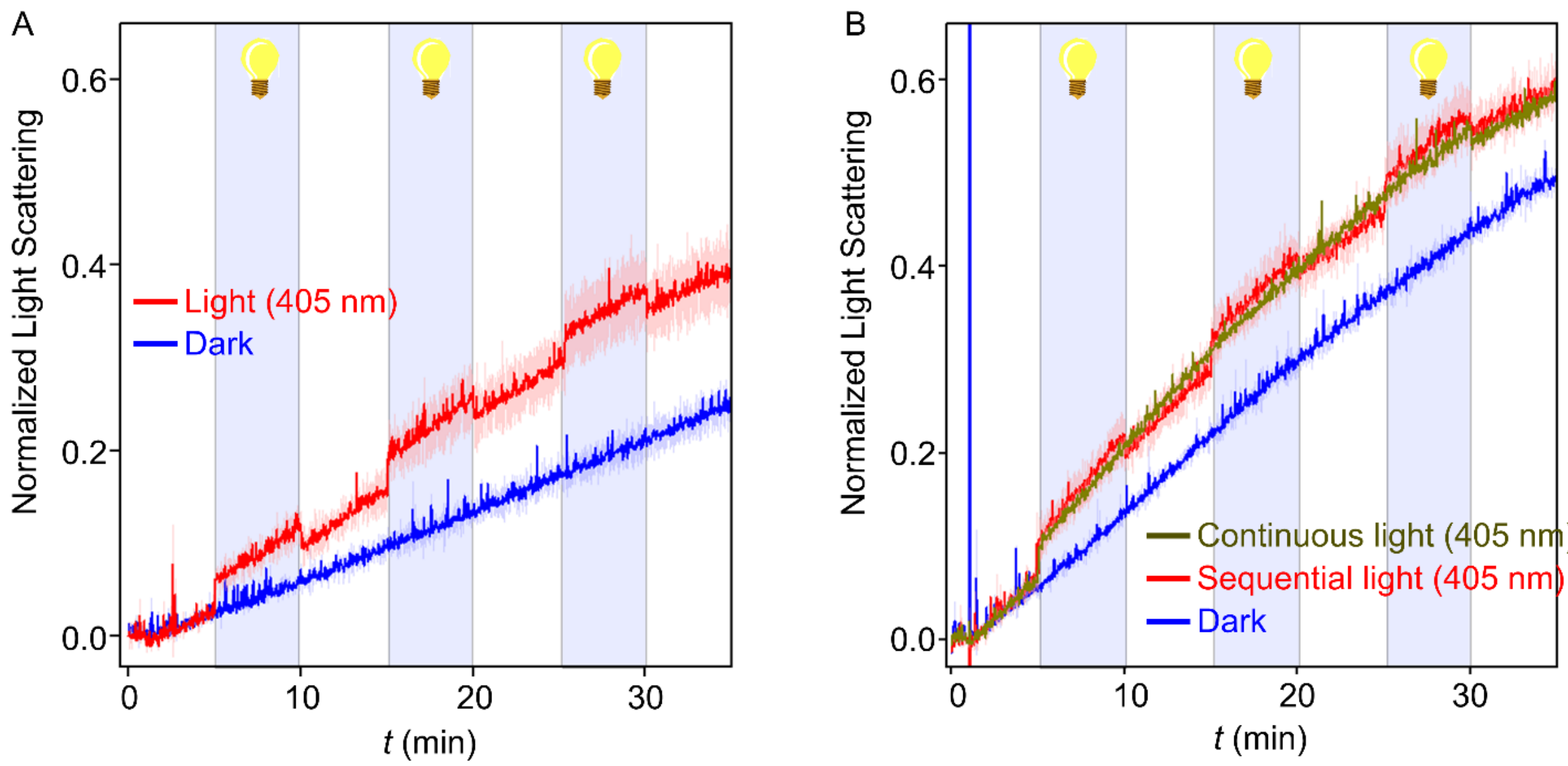

Figure S25. Normalized scattering intensity for compound **1** at 3 mol% (A) and at 7 mol% (B) in dark (blue) and under *in situ* irradiation (red). The dark yellow trace in Figure S27B shows the effect under continuous irradiation for 30 minutes. The minor additional increase in scattering intensity under irradiation is likely caused by stray light from the 405 nm irradiation source reaching the detector while the instrument is set to monitor scattering at 600 nm.

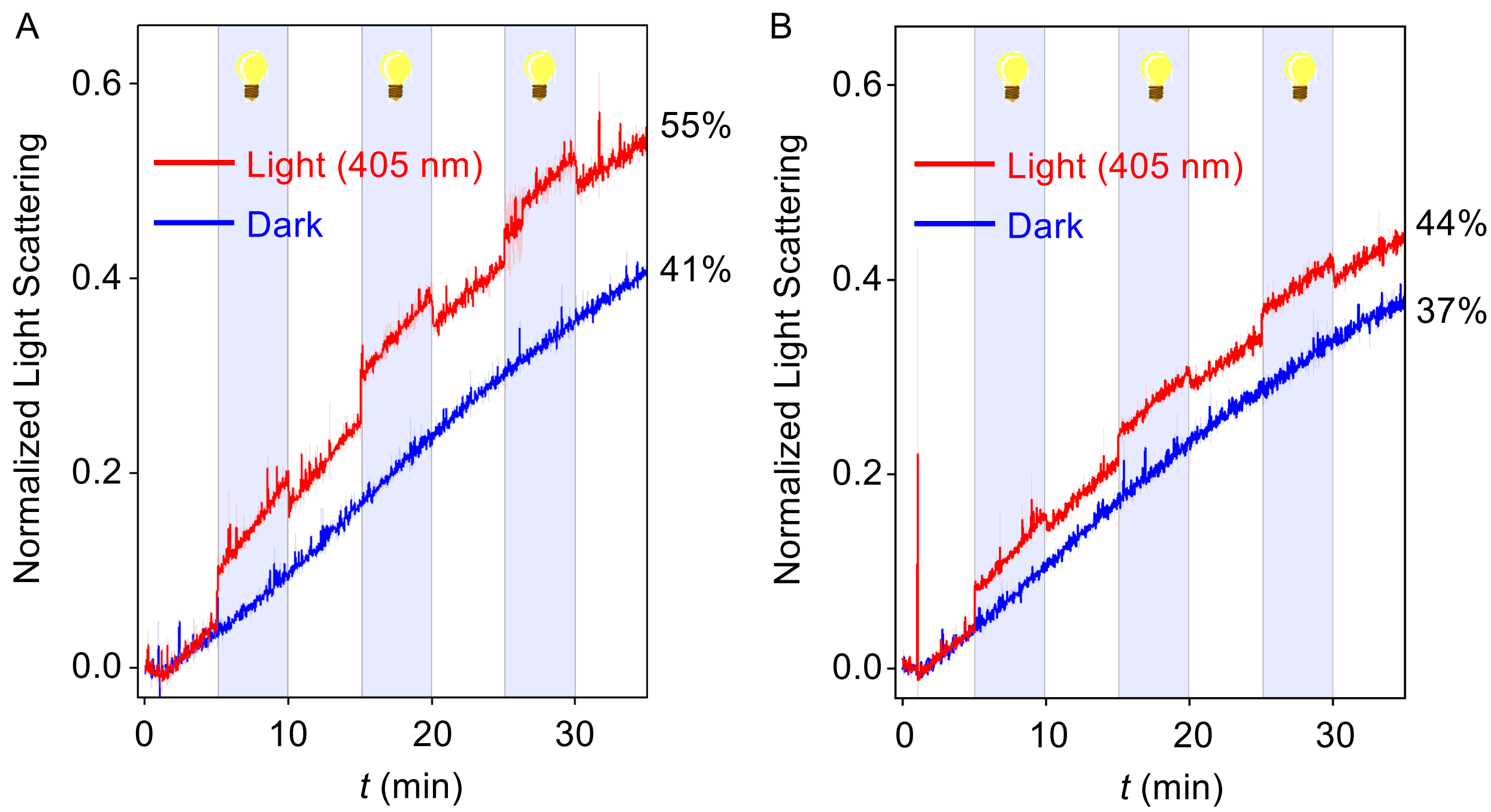

Figure S26. Normalized light scattering intensity as a measure of chloride transport across POPC (A) and POPC/cholesterol (85:15 mol%) (B) liposomes for compound **1** (5 mol% relative to lipid) under dark (blue) and during *in situ* irradiation with 405 nm light (red). The minor additional increase in scattering intensity under irradiation is likely caused by stray light from the 405 nm irradiation source reaching the detector while the instrument is set to monitor scattering at 600 nm.

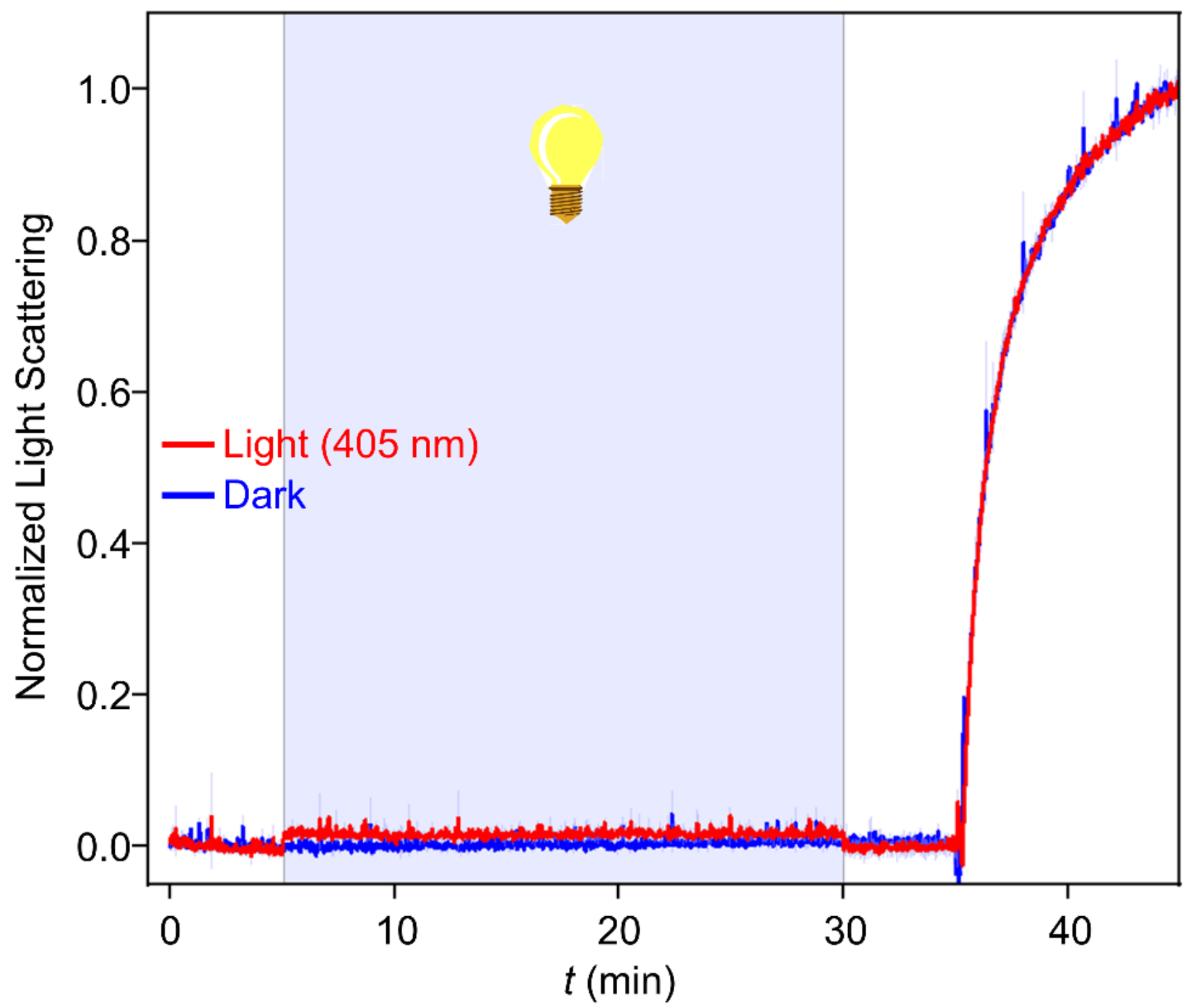

Figure S27. Normalized scattering intensity as a measure of chloride efflux from liposomes (without transporter) subjected to continuous *in situ* irradiation at 405 nm for 30 min. Prodigiosin and FCCP were added after 35 min to achieve the maximum chloride efflux. The absence of any response prior to their addition confirms that prolonged light exposure neither compromises the liposomal membrane nor induces chloride leakage.

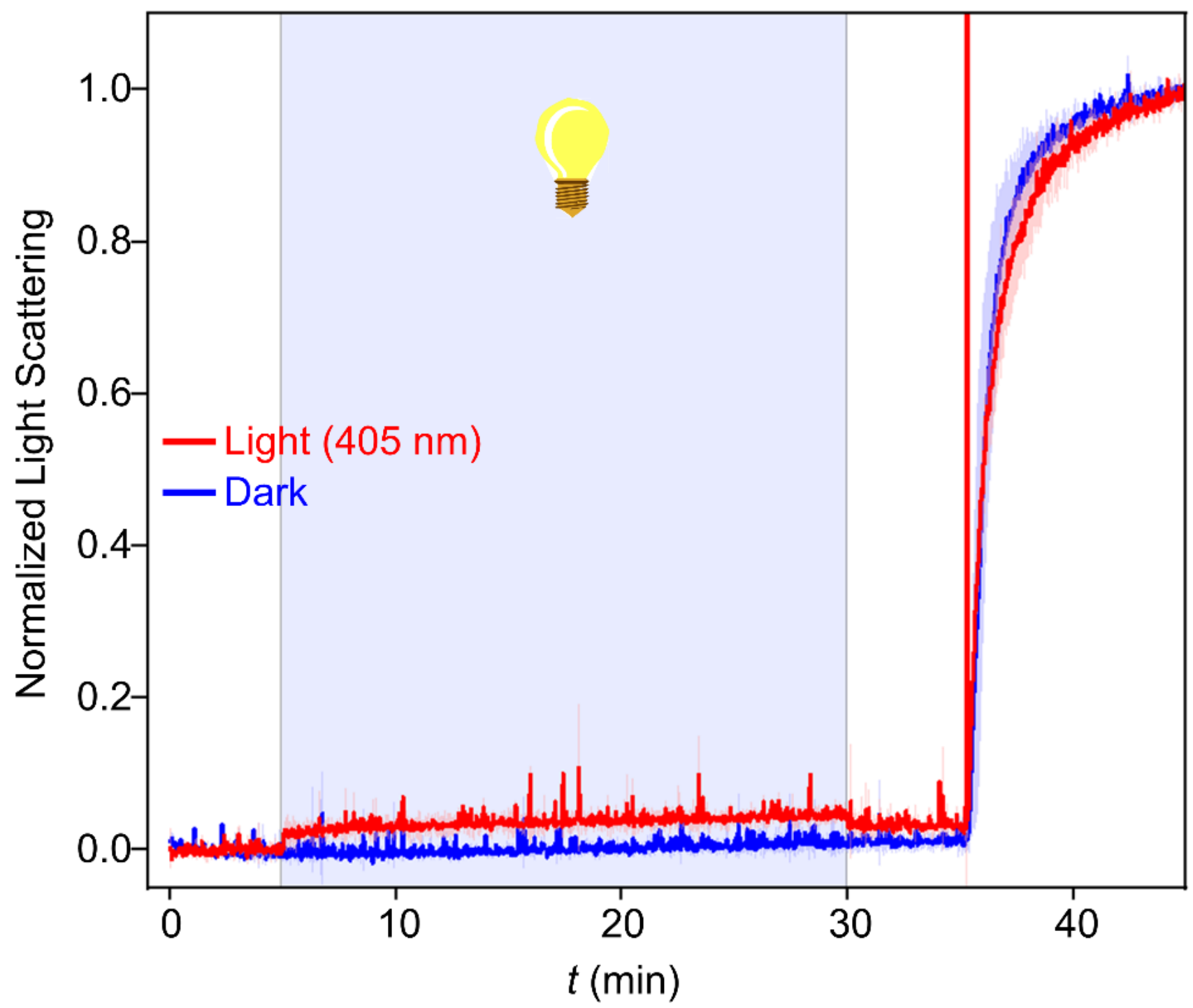

Figure S28. Normalized scattering intensity as a measure of chloride efflux from liposomes containing 7 mol% of compound **1** subjected to *in situ* irradiation at 405 nm for 30 min in the absence of valinomycin. Prodigiosin and FCCP were added after 35 min to achieve the maximum chloride efflux. The absence of a significant response during irradiation and before the addition of Prodigiosin and FCCP confirms that light exposure neither compromises membrane integrity nor causes chloride leakage. The minor increase in scattering intensity under irradiation is likely caused by stray light from the 405 nm irradiation source reaching the detector while the instrument is set to monitor scattering at 600 nm.

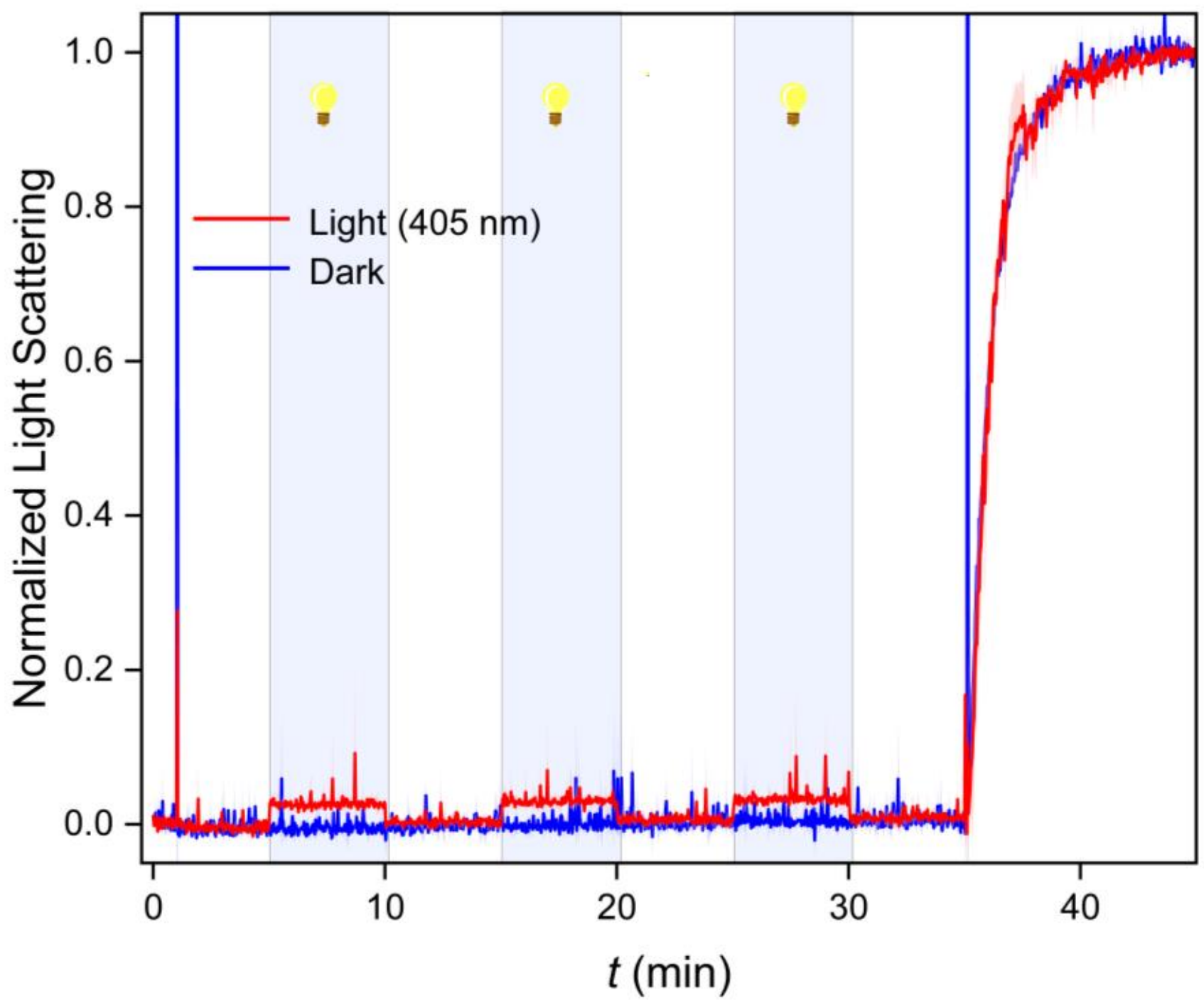

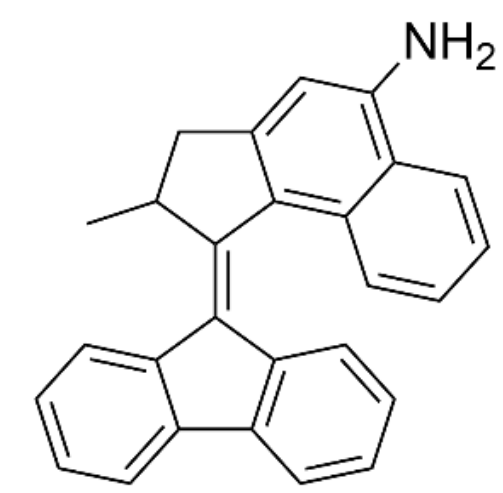

4

Figure S29. Normalized light scattering intensity as a measure of chloride transport for compound **4** (5 mol% relative to lipid) under dark (blue) and during *in situ* irradiation with 405 nm light (red). Prodigiosin and FCCP were added after 35 min to achieve the maximum chloride efflux. The absence of a significant response during irradiation and before the addition of Prodigiosin and FCCP confirms that light exposure neither compromises membrane integrity nor causes chloride leakage. The minor increase in scattering intensity under irradiation is likely caused by stray light from the 405 nm irradiation source reaching the detector while the instrument is set to monitor scattering at 600 nm.

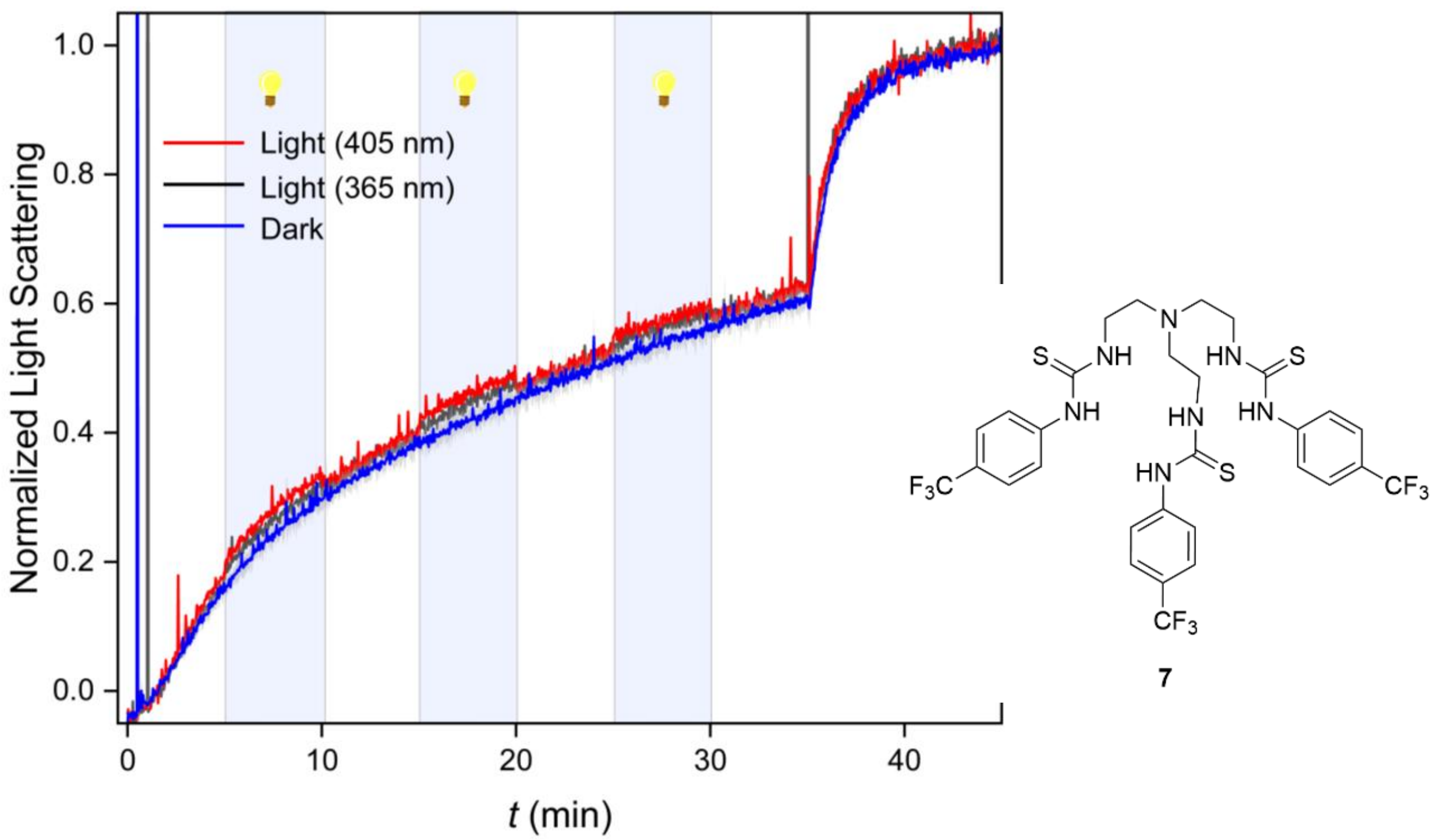

Figure S30. Normalized light scattering intensity as a measure of chloride transport for compound **7** (0.5 mol% relative to lipid) under dark (blue) and during *in situ* irradiation with 365 nm light (black) and 405 nm light (red). Prodigiosin and FCCP were added after 35 min to achieve the maximum chloride efflux. The absence of a significant response during irradiation and before the addition of Prodigiosin and FCCP confirms that light exposure neither compromises membrane integrity nor causes chloride leakage. The minor increase in scattering intensity under irradiation is likely caused by stray light from the 405 nm irradiation source reaching the detector while the instrument is set to monitor scattering at 600 nm.

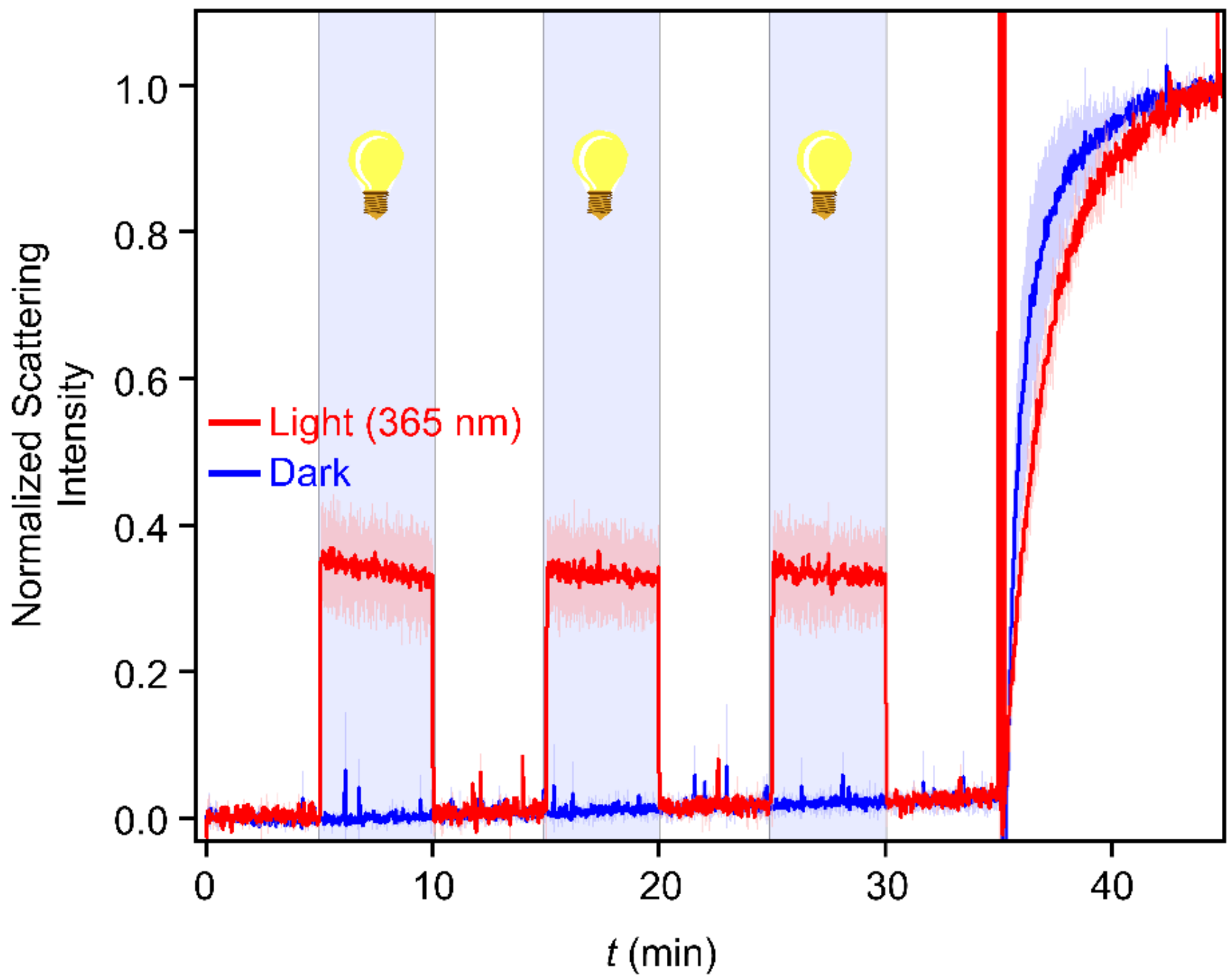

Figure S31. Normalized scattering intensity as a measure of chloride efflux from liposomes containing 5 mol% of compound **2** subjected to alternating dark and *in situ* 365 nm irradiation periods in the absence of valinomycin. Prodigiosin and FCCP were added after 35 min to achieve the maximum chloride efflux. The marked increase in scattering intensity during the irradiation period can be ascribed mostly to emission by compound **2** upon 365 nm irradiation (Figure S36). It does not reflect genuine transport since the scattering intensity overlaps with the one from the experiment performed in the dark after cessation of light. The absence of a genuine response before the addition of prodigiosin and FCCP confirms that light exposure neither compromises membrane integrity nor causes chloride leakage.

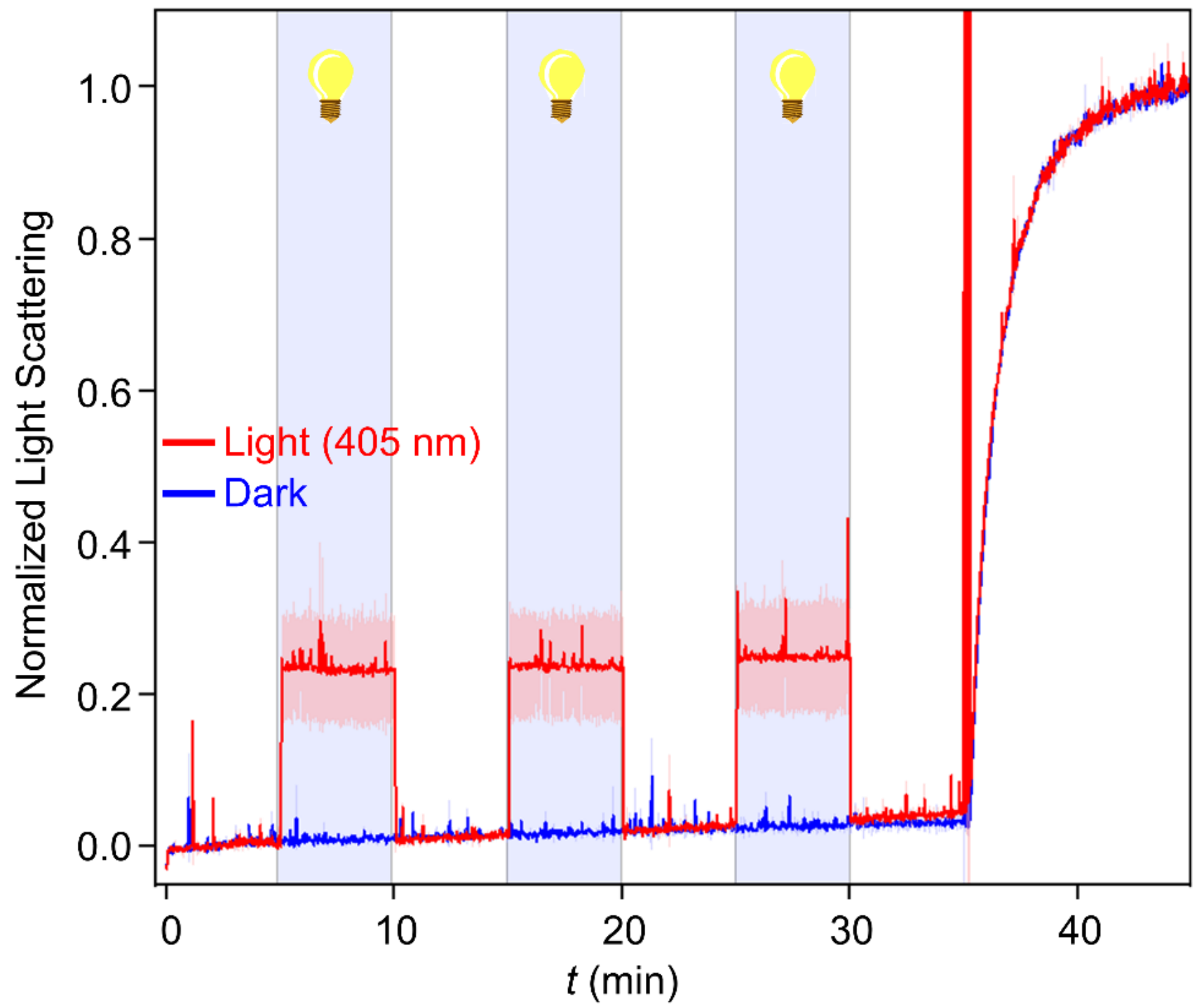

Figure S32. Normalized scattering intensity as a measure of chloride efflux from liposomes containing 5 mol% of compound **2** subjected to alternating dark and *in situ* 405 nm irradiation periods in the absence of valinomycin. Prodigiosin and FCCP were added after 35 min to achieve the maximum chloride efflux. The marked increase in scattering intensity during the irradiation period is most likely the result of emission by compound **2** upon 405 nm irradiation. It does not reflect genuine transport since the scattering intensity overlaps with the one from the experiment performed in the dark after cessation of light. The absence of a genuine response before the addition of prodigiosin and FCCP confirms that light exposure neither compromises membrane integrity nor causes chloride leakage.

HPTS Assay: A thin lipid film was acquired by drying a chloroform solution of a mixture of POPC and compound **2** (0.2 mol% with respect to POPC) in a round-bottom flask. This film was dried under vacuum for 16 h. The dried lipid film was hydrated by vortexing with a solution containing HPTS (1 mM) and NaCl (100 mM) buffered at pH 7.0 with 10 mM HEPES. The lipid suspension was then subjected to nine freeze/thaw cycles and was extruded 21 times through a 200 nm

polycarbonate membrane (NucleoporeTM) using an extruder set (Avanti Polar Lipids, Inc) to obtain the vesicle stock solution. The unentrapped HPTS was removed by size exclusion chromatography on a Sephadex® G-25 column using HEPES-buffered external NaCl (100 mM).

Vesicles containing NaCl (100 mM, buffered to pH 7.0 using 10 mM HEPES) and HPTS (1 mM) were suspended in NaCl (100 mM buffered to pH 7.0 using 10 mM HEPES) to make a test solution of 0.1 mM lipid in 2.5 mL external solution. The sample was stirred at 293 K and the fluorescence ratio of HPTS (λ_{ex} = 454 nm, λ_{em} = 510 nm, base form divided by λ_{ex} = 403 nm, λ_{em} = 510 nm, acid form) was recorded over time. In a different experiment, fluorescence intensity of only the base form of HPTS (λ_{ex} = 454 nm, λ_{em} = 510 nm) was recorded. A pulse of NaOH (25 µL of 0.5 M solution, final concentration 5 mM) was added to the vesicle suspension to generate a pH gradient of pH 7 inside and pH 8 outside at 30 s. After 370 s, detergent (25 µL of Triton X-100 (11 w%) in H_2O:DMSO (7:1 v/v)) was added to lyse the vesicles and after 430 s a final reading was taken. The fractional fluorescence intensity (I_f) was calculated using:

$$I_f = \frac{R_t - R_0}{R_d - R_0}$$

where R_t is the fluorescence ratio at time t, R_0 is the fluorescence ratio at time 0 s and R_d are the fluorescence ratio at the end of the experiment, after the addition of detergent at time t = 370 s.

For the single wavelength excitation, the fractional fluorescence intensity (I_f) was calculated using:

$$I_f = \frac{I_t - I_0}{I_d - I_0}$$

where I_t is the fluorescence intensity at time t, I_0 is the fluorescence intensity at time t = 0 s, and I_d is the fluorescence intensity at the end of the experiment, after the addition of detergent at time t = 370 s.

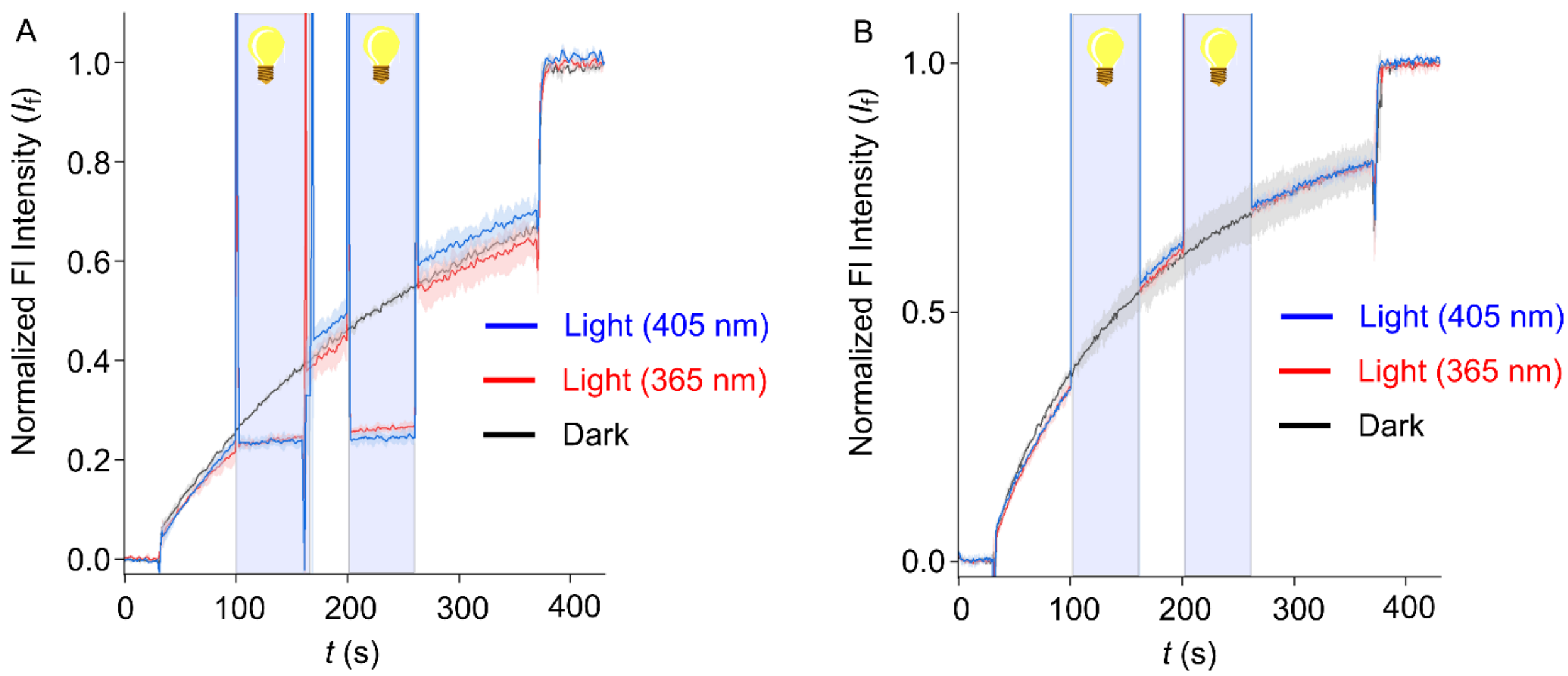

Figure S33. Effect of light irradiation on the normalized fluorescence intensity in the HPTS assay using POPC liposomes containing compound **2** (0.2 mol% relative to lipid). Shown are the normalized fluorescence response of the HPTS dye when excited using both 403 and 454 nm pulses from the instrument, plotted as the intensity ratio (I_{454}/I_{403}) (A), and when excited using 454 nm only (B).

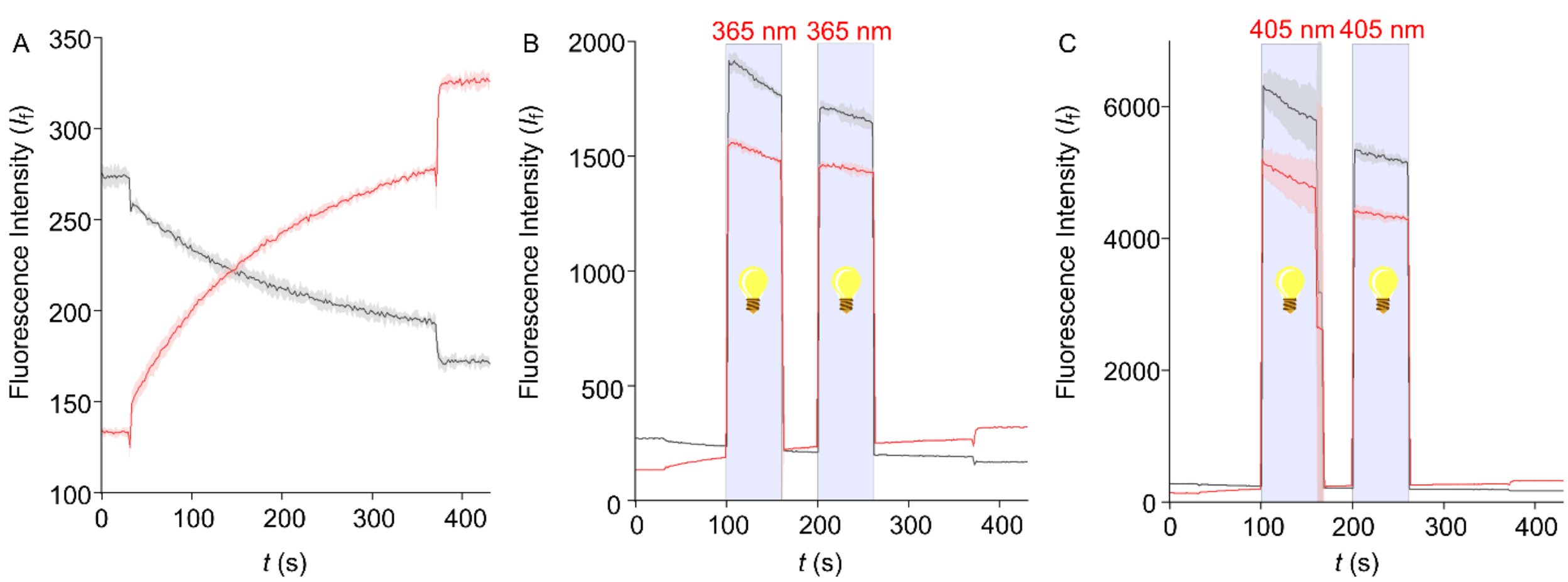

Figure S34. Raw traces of the fluorescence emission during the transport mediated by compound **2** in dark conditions (0.2 mol% relative to lipid). The red trace originates from the 454 nm excitation of HPTS dye, and the black trace corresponds to the 403 nm excitation (A). Effect of light irradiation using 365 nm LED (B) and 405 nm (C) in the same assay (raw traces).

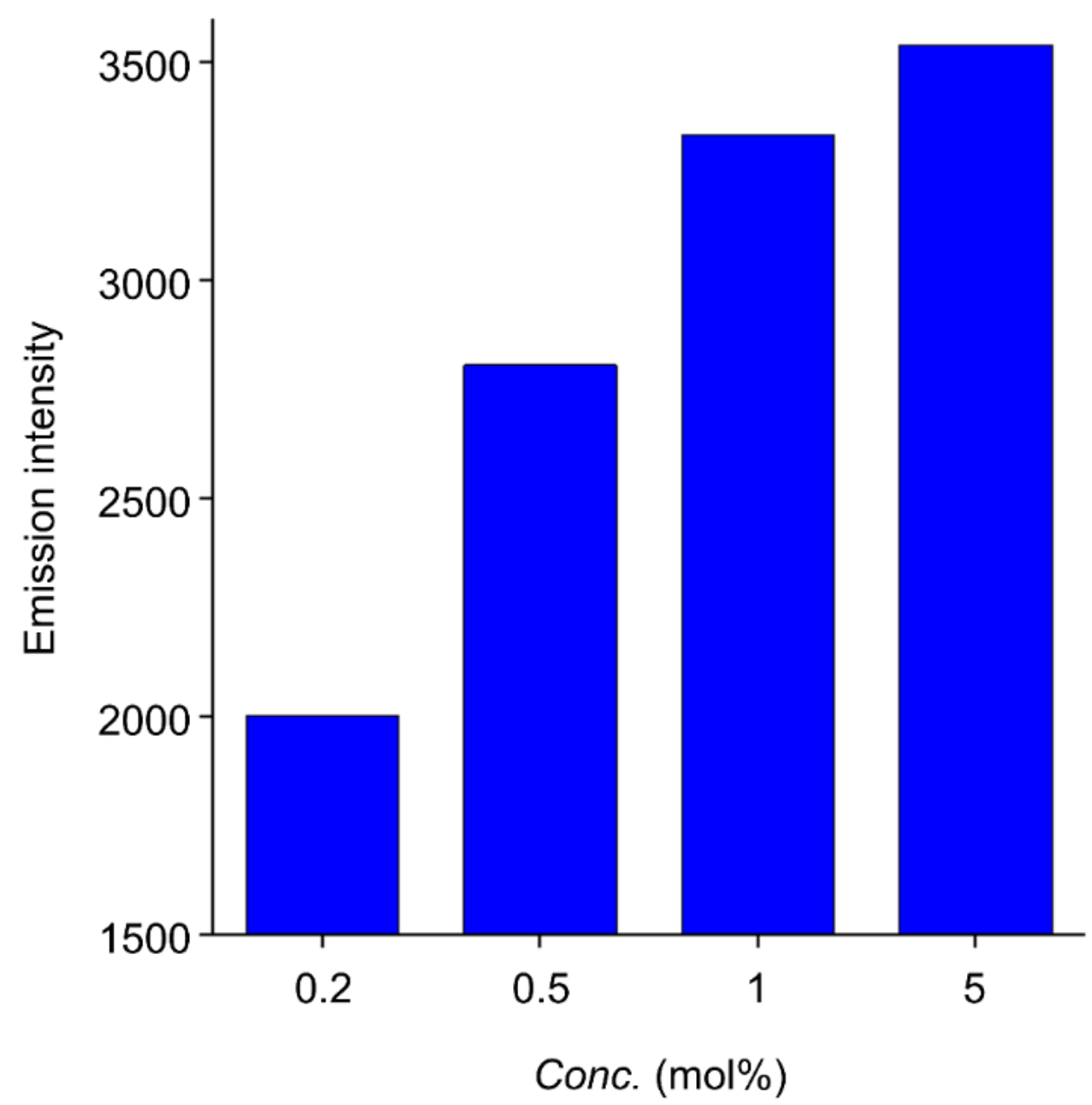

Figure S35. Emission intensity upon *in situ* irradiation at 365 nm during the HPTS transport assay using liposomes containing compound **2** at increasing loadings. The gradual increase in emission with elevated transporter concentration demonstrates that the observed marked increases in fluorescence intensity during the irradiation periods is dominated by the intrinsic fluorescence of compound **2** itself (Figure S36).

Of note, the HPTS assay was not performed for compound **1** because of its strong absorption at both 403 nm and 450 nm (Figure S31), directly overlapping with the excitation wavelengths used for the HPTS dye (405/454 nm), making this assay unsuitable to study this compound.

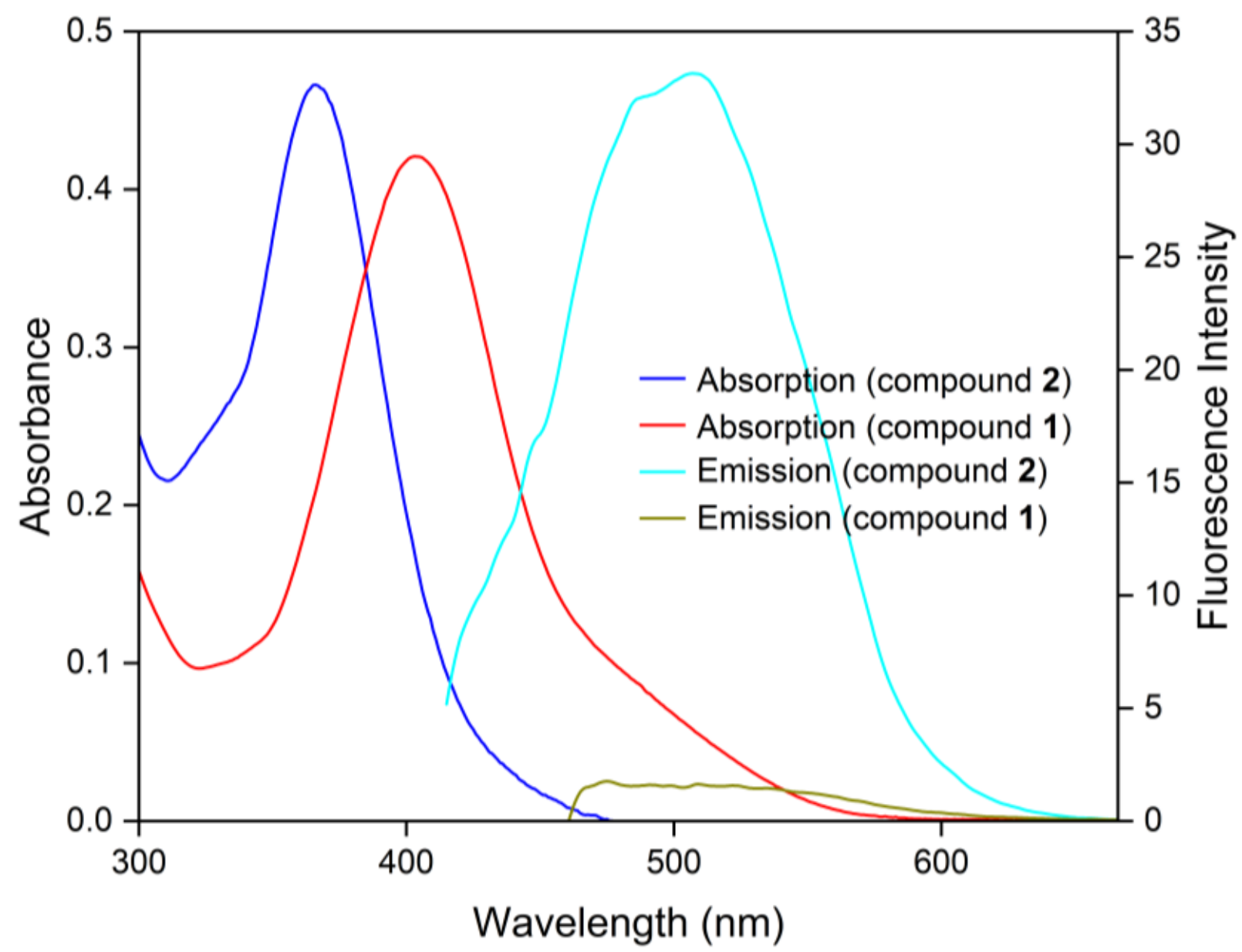

Figure S36. UV–vis absorption and steady-state fluorescence spectra of compound **1** (slow motor transporter) and compound **2** (fast motor transporter) recorded in acetonitrile. Absorption spectra were measured at a concentration of 2.50×10^{-5} M, showing maxima at 403 nm for compound **1** and 365 nm for compound **2**. Steady-state fluorescence emission spectra were recorded at a concentration of 3.00×10^{-6} M upon excitation at 405 nm for compound **1** and 365 nm for compound **2**. The spectra demonstrate that compound **2** (thioxanthene-derived motor) exhibits intense fluorescence with a maximum centered around 500–520 nm, while compound **1** (fluorenyl-derived motor) shows weaker but still detectable emission in the same spectral region.

The absorption and emission wavelengths of these molecular motors coincide with the excitation and emission wavelengths used in the HPTS-based ion transport assay (λ_{ex} = 403 nm and 454 nm, λ_{em} = 510 nm), leading to potential photophysical cross-interference between the molecular motor and the encapsulated dye. Upon instrumental excitation at 403 nm, both the motor and the HPTS dye can be simultaneously excited, resulting in combined emissions in the 500–520 nm region. Consequently, two main effects can distort the fluorescence ratio (I_{454}/I_{403}): (i) direct fluorescence

contribution from the motor adds to the overall emission intensity attributed to HPTS, and (ii) *in situ* irradiation to isomerize the motor during the measurement also excites the HPTS dye, leading to an increase in the detected emission. As a result, the observed changes in the I_{454}/I_{403} ratio may not accurately represent the actual ion transport process, but rather a convolution of photophysical effects from both species. To minimize this interference, for compound **2**, using only the 454 nm excitation wavelength provides a more reliable indication of the transport process.

Table S1. Comparison of Feringa-type molecular motor-based transmembrane transport systems, summarizing the type of motor scaffold used, assays used, ions transported and increase in efficiency observed under light irradiation.

Report	Motor type	Assay used	Ions	Enhancement
Barboiu and Giuseppone (2021)[4]	Thioxanthene-based (fast)	Fluorescence-based (HPTS), Patch-clamp (BLM)	Na^+, K^+, and Rb^+	Upto ~4 fold
Qu and Bao (2022)[5]	Thioxanthene-based (fast)	Fluorescence-based (HPTS), Patch-clamp (BLM)	K^+	~3 fold
Zeng and coworkers (2024)[6]	Thioxanthene-based (fast)	Fluorescence-based (HPTS)	Li^+, Na^+, K^+, Rb^+, and Cs^+	1.7-2.9 fold
This work	Fluorene-based (slow)	Ion-selective electrode (ISE), Osmotic response	Cl^-	1.85 fold
This work	Thioxanthene-based (fast)	Fluorescence-based (HPTS), Osmotic response	Cl^-	None

References

[1] a) J. Kaleta, J. Chen, G. Bastien, M. Dračínský, M. Mašát, C. T. Rogers, B. L. Feringa, J. Michl, *J. Am. Chem. Soc.* **2017**, *139*, 10486–10498; b) J. Sheng, C. L. F. van Beek, C. N. Stindt, W. Danowski, J. Jankowska, S. Crespi, D. R. S. Pooler, M. F. Hilbers, W. J. Buma, B. L. Feringa, *Sci. Adv.* **2025**, *11*, eadr9326.

[2] C. Feldmeier, H. Bartling, E. Riedle, R. M. Gschwind, *J. Magn. Reson.* **2013**, *232*, 39-44.

[3] C. Frassineti, S. Ghelli, P. Gans, A. Sabatini, M. S. Moruzzi, A. Vacca, *Anal. Biochem.* **1995**, *231*, 374–382.

[4] W.-Z. Wang, L.-B. Huang, S.-P. Zheng, E. Moulin, O. Gavat, M. Barboiu, N. Giuseppone, *J. Am. Chem. Soc.* **2021**, *143*, 15653–15660.

[5] H. Yang, J. Yi, S. Pang, K. Ye, Z. Ye, Q. Duan, Z. Yan, C. Lian, Y. Yang, L. Zhu, D.-H. Qu, C. Bao, *Angew. Chem. Int. Ed.* **2022**, *61*, e202204605.

[6] S. Deng, Z. Li, L. Yuan, J. Shen, H. Zeng, *Nano Lett.* **2024**, *24*, 10750–10758.
